# High-dimensional CRISPR-SERS interactomics tracks the topological evolution of plant viral pathogenesis and therapeutic response

**DOI:** 10.1039/d6sc03368g

**Published:** 2026-06-04

**Authors:** You Dou, Gang Wu, Jun Li, Yuting Wang, Nanke Chen, Tianbing Xu, Xiaobo Cai, Tuotuo Zhang, Xinwei Zhang, Xiangyang Li, Junrong Li, Yao Sun

**Affiliations:** a College of Chemistry, Central China Normal University Wuhan 430079 China junrong.li@ccnu.edu.cn; b The Department of Radiology, Tongji Hospital of Tongji Medical College of Huazhong University of Science and Technology China; c National Key Laboratory of Agricultural Microbiology, College of Biomedicine and Health, Huazhong Agricultural University Wuhan 430070 China sunyaogbasp@ccnu.edu.cn; d State Key Laboratory of Green Pesticide, Key Laboratory of Green Pesticide and Agricultural Bioengineering, Ministry of Education, Guizhou University Guiyang 550025 China xyli1@gzu.edu.cn; e College of Chemistry, Huazhong Agricultural University Wuhan 430070 China; f Hubei Jiangxia Laboratory Wuhan 430020 China

## Abstract

Precision agriculture is currently limited by a reliance on viral load quantification, a static metric that obscures the dynamic molecular arms race between invading pathogens and host immunity. To overcome this limitation, we present a multidimensional surface-enhanced Raman spectroscopy (SERS) platform designed to map the real-time topology of the host–virus interactome. Overcoming the spectral constraints of conventional assays, we engineered a *de novo* library of isomeric Raman reporters *via* rational positional and electronic tuning, enabling high-density spectral coding. Furthermore, by integrating CRISPR-dCas9 as an isothermal recognition module, we ensure multiplexed signals accurately reflect the stoichiometric integrity of viral and host gene expression due to the elimination of thermodynamic biases of traditional DNA hybridization. Applying this platform to the *Nicotiana benthamiana*–Potato virus Y (PVY) pathosystem, we dissect the longitudinal evolution of infection under ningnanmycin (NNM) treatment. Beyond enabling presymptomatic diagnosis at Day 1, our interactome analysis reveals that chronic infection stabilizes into a regulatory triangle clamped by the Vpg–PUB4 interface, whereas therapeutic relapse manifests as a chaotic, Vpg-centralized network. This work establishes interactome topology as a superior diagnostic metric to viral load, providing a blueprint for identifying drug resistance mechanisms and targeting the specific protein–protein interactions that drive viral persistence.

## Abstract 2

Integrating a rationally designed isomeric Raman library with CRISPR-dCas9 recognition, this multiplexed SERS platform maps the topological evolution of host–virus interactomes instead of the static viral load detection.

## Graphical abstract

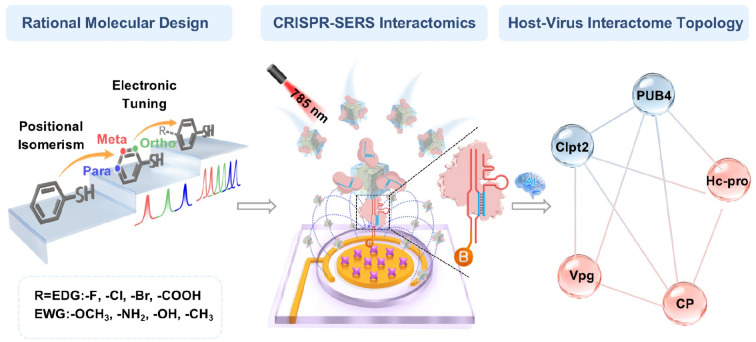

## Introduction

Plant viral pathogenesis is not a unilateral event but a dynamic molecular arms race, defined by the intricate and rapidly evolving interactome between invading viral factors and the host immune surveillance system.^1–4^ The ultimate outcome of plant viral infection is dictated by how effectively the virus can co-opt host cellular machinery^5^ while suppressing RNA silencing^6–8^ and ubiquitin-mediated degradation pathways.^9,10^ However, conventional diagnostic modalities, such as qRT-PCR or ELISA,^11–16^ predominantly focus on detecting viral load. This single metric approach fails to capture the reciprocal dynamics of the host–pathogen battlefield, often obscuring the mechanistic underpinnings of therapeutic success or failure. To bridge this gap, it is essential to move beyond simple pathogen detection toward the simultaneous, multiplexed monitoring of both viral replication complexes and host defense regulators in real-time. By deciphering the real-time topology of this interaction network, we can gain critical insights into viral escape mechanisms and inform the rational selection or development of antiviral therapeutics.

Surface-enhanced Raman spectroscopy (SERS) has emerged as a powerful multiplexed analytical tool due to its single-molecule level sensitivity and inherently narrow spectral linewidths.^17–25^ However, unleashing the full potential of SERS for systems biology is currently impeded by two fundamental bottlenecks. First, conventional Raman reporters are randomly selected through empirical screening of commercially available molecular dyes without clear structure–property relationships,^26,27^ thus restricting the spectral coding capacity. Second, the conventional hybridization-based approach using multiple single-stranded DNA (ssDNA) for respective target identification is inherently biased by sequence-dependent thermodynamics, melting temperature variations, and secondary structures, which prevents uniform identification efficiency and results in an inaccurate reflection of the true stoichiometric ratios of the vial and host genes.^28–30^ In contrast, CRISPR/dCas9-mediated recognition enables PAM-dependent interrogation and sgRNA-guided formation of stable R-loops on native double-stranded DNA, allowing highly sequence-specific target recognition without thermal denaturation.^31,32^ This protein-assisted recognition mechanism provides improved structural tolerance, kinetically uniform target engagement, and enhanced robustness against matrix interference, thereby offering more reliable and quantitative signal readouts for multiplexed biosensing. As such, establishing a precise interactome requires a SERS platform that integrates *ab initio* rational Raman reporter design with a kinetically uniform CRISPR-based nucleic acid recognition system.

**Fig. 1:**
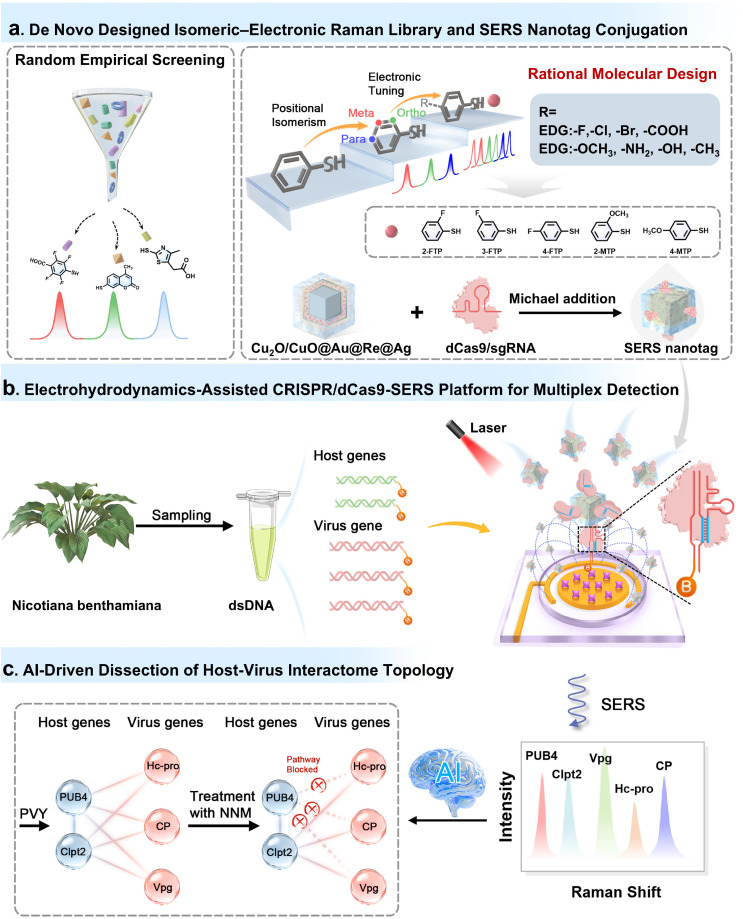
Development of the SERS platform to investigate the interactome of plant and viral infection. (a) The molecular engineering of an isomeric Raman reporter library with tunable Raman spectral channels enhanced by Cu_2_O/CuO@Au@Ag, as well as the integration with dCas9/sgRNA for multiplexed targeting and labeling. (b) EHD-assisted SERS platform for simultaneous detection of both plant and viral nucleic acids. (c) The AI-driven analysis for revealing the interactome network of the plant and virus.

Herein, we introduce a multidimensional SERS platform to decipher the complex interplay within the *Nicotiana benthamiana* (*N. benthamiana*) and potato virus Y (PVY) pathosystem, decoding the interaction network that drives the transition from viral invasion to host recovery (Fig. 1). To overcome spectral limitations, we develop a *de novo* isomeric Raman reporter library (24 molecules) with programmable signal modulation through rational positional isomerism and electronic substituent tuning (Fig. 1a). These Raman reporters were enhanced by cubic Cu_2_O/CuO@Au@Ag nanostructures to maximize electromagnetic and chemical enhancement. Crucially, we replaced thermodynamic hybridization with a CRISPR-dCas9 recognition module. By using dCas9/sgRNA as the targeting unit, we ensure that the binding of all viral and host nucleic acids is driven by a uniform enzymatic mechanism rather than variable hybridization kinetics, thereby preserving the accurate stoichiometric integrity of the biological sample. With a further inclusion of electrohydrodynamics (EHD) for rapid target capture and identification, the SERS platform enables the high-throughput construction of host–virus interactomes (Fig. 1b). Integrated with AI-driven topology analysis (Fig. 1c), this SERS platform goes beyond early diagnosis to reveal the network dynamics of therapeutic efficacy, identifying the topological signatures of viral escape and guiding the development of resilient antiviral strategies.

## Results and discussion

### Molecular engineering of an isomeric Raman reporter library

**Fig. 2:**
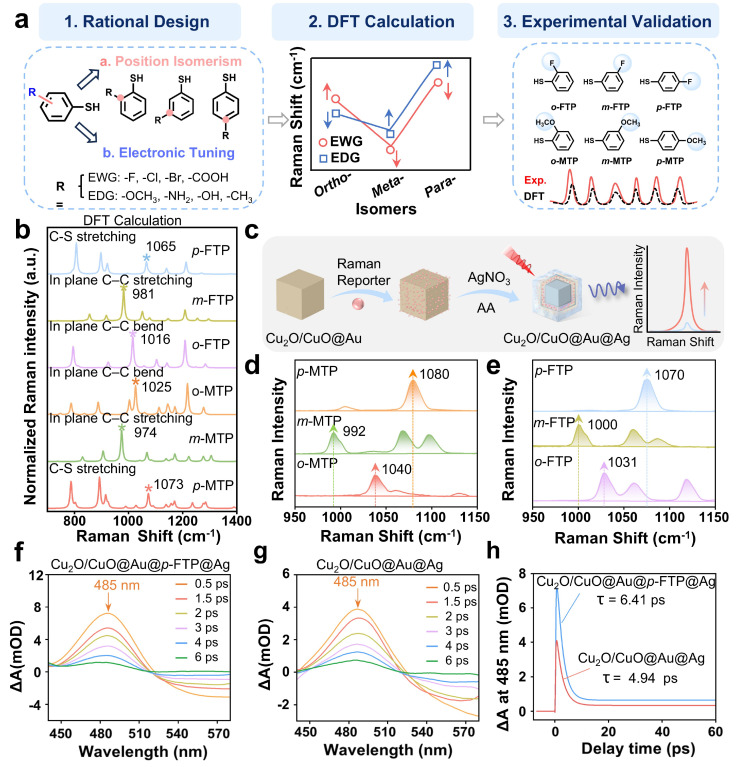
Molecular engineering of an isomeric Raman reporter library and its involvement in CM enhancement for signal amplification. (a) The design strategy of the isomeric Raman reporters. (b) DFT-calculated Raman spectra of the isomeric reporters with assignments of the dominant vibrational modes. (c) Schematic representation of signal amplification for Raman reporters using Cu_2_O/CuO@Au@Ag nanostructures. (d and e) Corresponding SERS spectra enhanced by Cu_2_O/CuO@Au@Ag nanostructures. (f and g) TA spectra of Cu_2_O/CuO@Au@*p*-FTP@Ag and Cu_2_O/CuO@Au@Ag nanostructures. (h) TA dynamics of Cu_2_O/CuO@Au@*p*-FTP@Ag and Cu_2_O/CuO@Au@Ag at 485 nm.

To achieve precise and programmable modulation of Raman signals, we engineered a library of isomeric Raman reporters (SI, Fig. S1) by exploiting rational positional isomerism and electronic substituent effects. This design strategy was validated through density functional theory (DFT) simulations and experimental characterization (Fig. 2a). The Raman reporters were synthesized from substituted iodobenzene precursors *via* a copper-catalyzed C–S coupling reaction (Fig. S2), with chemical structures confirmed by ^1^H and ^13^C NMR. To achieve precise and programmable modulation of Raman signals, we utilized thiophenol as a core scaffold, employing a positional-isomer engineering approach coupled with electronic substituent effects to rationally guide the design of Raman reporters. DFT calculations on six representative molecules were first carried out to validate the design principles and elucidate how symmetry perturbation and electronic fine-tuning control vibrational mode coupling and force constants (Fig. 2a). These molecules were subsequently synthesized and fully characterized by ^1^H and ^13^C NMR (SI for full spectra), while Cu_2_O/CuO@Au@Ag nanostructures served as SERS-active substrates to enhance the signal. The experimental spectra closely matched theoretical predictions. Guided by these insights, the approach was extended to a library of 24 compounds (Fig. S3), confirming the generality and predictive power of the design strategy.

Using six representative reporters, DFT calculations elucidated how molecular symmetry and electronic effects hierarchically dictate the spectral fingerprint. First, positional isomerism selectively activates specific vibrational modes (Fig. 2b). For *para*-isomers, the preserved structural symmetry and resonance donation generate a dominant, high-frequency C–S stretching mode (∼1065–1073 cm^−1^). In contrast, *meta*-substitution disrupts this conjugative communication, activating a lower-energy in-plane C–C stretching mode (∼974–981 cm^−1^). Meanwhile, the *ortho*-isomer introduces steric distortion, enhancing benzene ring coupling and generating a characteristic intermediate peak associated with the in-plane C–C bending mode (∼1016–1025 cm^−1^).

Superimposed on these symmetry rules, electronic effects further fine-tune the Raman shifts by altering the force constants of the vibrational bonds. Comparing the electron-donating methoxy group (–OCH_3_, MTP series) with the electron-withdrawing fluoro group (–F, FTP series) reveals divergent trends (Fig. 2b). For *ortho*- and *para*-isomers, the electron-donating capability of –OCH_3_ strengthens the C–C and C–S bonds, inducing a blue-shift relative to the fluoro-analogs (*e.g.*, *p*-MTP at 1073 cm^−1^*vs.p*-FTP at 1065 cm^−1^). Conversely, in the *meta*-position, the introduction of –OCH_3_ softens the ring-breathing mode compared to –F, resulting in a red-shift (*e.g.*, *m*-MTP at 974 cm^−1^*vs.m*-FTP at 981 cm^−1^). We next validated these theoretical designs by measuring the SERS signals of these reporters on Cu_2_O/CuO@Au@Ag nanostructures (Fig. 2c). The experimental SERS spectra exhibited excellent fidelity to the DFT predictions, with the characteristic peaks matching the simulated trends for both the MTP (Fig. 2d) and FTP (Fig. 2e) series.

Beyond their spectral tunability, the isomeric reporters were designed to actively participate in chemical enhancement (CM) mechanisms to maximize signal output. Taking *p*-FTP as a representative model, we utilized femtosecond transient absorption (TA) spectroscopy to probe how the reporter molecule modulates ultrafast charge carrier dynamics (Fig. 2f and S4). Upon anchoring *p*-FTP (Fig. S5) to the Cu_2_O/CuO@Au@Ag surface, the system exhibited a marked shift in decay kinetics compared to the bare nanostructure (Fig. 2g). Specifically, the presence of the *p*-FTP reporter extended the electron relaxation lifetime (*τ*) from 4.94 ps to 6.41 ps (Fig. 2h). This prolonged lifetime indicated that the reporter molecule did not merely scatter light but acted as an effective charge acceptor, facilitating energetic charge transfer (CT) from the excited nanostructure to the molecular orbitals of the Raman reporter. This confirmed that the isomeric reporters served as active components in the enhancement process, leveraging the chemical mechanism to boost detection sensitivity.^33,34^

### Synthesis and characterization of Cu_2_O/CuO@Au@Ag hybrid nanostructures

**Fig. 3:**
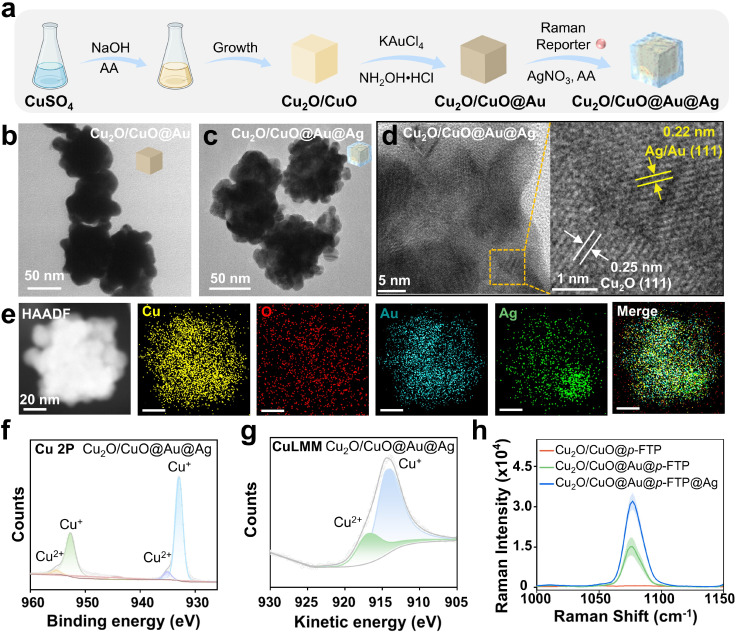
Synthesis and characterization of Cu_2_O/CuO@Au@Ag nanostructures. (a) Synthesis diagram for the stepwise formation process of Cu_2_O/CuO@Au@Ag nanostructures. (b and c) TEM images of Cu_2_O/CuO@Au and Cu_2_O/CuO@Au@Ag, respectively. (d) HR-TEM image and lattice spacing of Cu_2_O/CuO@Au@Ag nanostructures. (e) HAADF-STEM image and elemental distribution of the Cu_2_O/CuO@Au@Ag nanostructures. Scale bar: 20 nm. (f) XPS scan of Cu in Cu_2_O/CuO@Au@Ag nanostructures. (g) High-resolution Cu LMM Auger spectra of Cu_2_O/CuO@Au@Ag nanostructures. (h) SERS signals of *p*-MTP enhanced by Cu_2_O/CuO, Cu_2_O/CuO@Au and Cu_2_O/CuO@Au@Ag nanostructures.

To engineer a substrate capable of ultra-sensitive signal amplification, we rationally designed a semiconductor–noble metal hybrid heterostructure (*i.e.*, Cu_2_O/CuO@Au@Ag nanostructures). This architecture was selected to maximize plasmonic coupling efficiency and electromagnetic field confinement through the synergistic contributions of each component. Specifically, the Cu_2_O/CuO core serves as a low-cost and structurally tunable scaffold, while the Au interlayer ensures excellent chemical stability and facilitates robust thiol-based immobilization of Raman reporters. Furthermore, the outer Ag shell further enhances electromagnetic field confinement and generates abundant interparticle and intraparticle “hot spots” within the Au–Ag nanogaps, which is critical for achieving ultrasensitive detection. The synthesis began with the preparation of cost-effective, cubic Cu_2_O/CuO cores *via* a facile aqueous-phase reaction. Subsequently, a gold shell was deposited onto these semiconductor cores through a synergistic mechanism combining *in situ* galvanic replacement with hydroxylamine (NH_2_OH)-mediated reduction. Crucially, the galvanic replacement between the Cu_2_O surface and AuCl_4_^−^ initiated the formation of Au seeds, preventing self-nucleation and ensuring uniform shell growth. Following the functionalization of the Au layer with isomeric Raman reporters (*via* Au–S bonding), a final silver shell was grown. Due to this design, the Raman reporters were confined between the Au and Ag layers, making them less prone to detachment while enabling them to experience a strongly coupled electromagnetic field (Fig. 3a).

The morphological and structural evolution of the nanostructures was rigorously confirmed through comprehensive microscopic analysis. Transmission electron microscopy (TEM) images illustrated the progressive transformation from the smooth, cubic Cu_2_O/CuO cores (Fig. S6) to the intermediate Cu_2_O/CuO@Au and finally to the rougher, slightly larger Cu_2_O/CuO@Au@Ag nanostructures (Fig. 3b and c). High-resolution TEM (HR-TEM) further revealed the high crystallinity of the hybrid system, displaying distinct lattice fringes with interplanar spacings of 0.22 nm and 0.25 nm, corresponding to the Ag/Au (111) and Cu_2_O (111) planes, respectively (Fig. 3d). This confirmed the successful formation of a tightly interfaced heterojunction. Energy-dispersive X-ray spectroscopy (EDS) elemental mapping validated the intended core–shell–shell architecture, visualizing a copper–oxygen rich core encapsulated by concentric distributions of gold and silver (Fig. 3e). Consistent with these observations, dynamic light scattering (DLS) tracked the stepwise increase in hydrodynamic diameter from 37.8 nm (bare core) to 68.7 nm (@Au) and finally 79.9 nm (@Au@Ag) (Fig. S7a).

To elucidate the surface chemistry and oxidation states, we performed X-ray photoelectron spectroscopy (XPS). The high-resolution Cu 2p spectrum (Fig. 3f) and the Cu LMM Auger spectrum (Fig. 3g) exhibited characteristic features of both Cu^+^ and Cu^2+^ species. Notably, relative to the bare semiconductor cores, the metallized nanostructures showed an increased proportion of Cu^2+^, chemically corroborating the galvanic replacement mechanism where Cu^+^ is oxidized during Au deposition. Meanwhile, the Au 4f and Ag 3d spectra (Fig. S7b and S7c) displayed distinct doublets characteristic of their metallic states (Au^0^ and Ag^0^). This evolution was further mirrored in UV-Vis absorption spectroscopy, which showed the emergence and redshift of localized surface plasmon resonance (LSPR) bands corresponding to the sequential coating of Au and Ag shells (Fig. S7d).

Finally, we evaluated the SERS efficiency of the hybrid nanostructures using *p*-FTP as the Raman reporters. As shown in Fig. 3h, the stepwise construction of the heterostructure resulted in dramatic signal amplification. While the semiconductor core and the intermediate Au shell yielded negligible or moderate signals, the final Cu_2_O/CuO@Au@Ag nanostructures exhibited a remarkable Raman enhancement. This performance was attributed to the synergistic combination of the semiconductor–metal chemical enhancement and the intense electromagnetic fields generated within the embedded reporter nanogaps. In addition, the Cu_2_O/CuO@Au@Ag nanostructures enhanced stronger Raman signals than conventional Au and Ag nanoparticles (Fig. S7e). The enhancement factor (EF) of the Cu_2_O/CuO@Au@Ag nanostructures was calculated to be 6.3 × 10^6^ (Fig. S8), which was higher than those of previously reported noble metal and semiconductor systems, underpinning the high-sensitivity DNA profiling. Furthermore, the Cu_2_O/CuO@Au@Ag nanostructures exhibited excellent signal consistency (RSD = 8.3%) and high longitudinal stability over 7 days (RSD = 6.4%), allowing for reliable and ultrasensitive DNA analysis (Fig. S9 and S10).

### Bioinformatic analyses reveal rational gene profiles

**Fig. 4:**
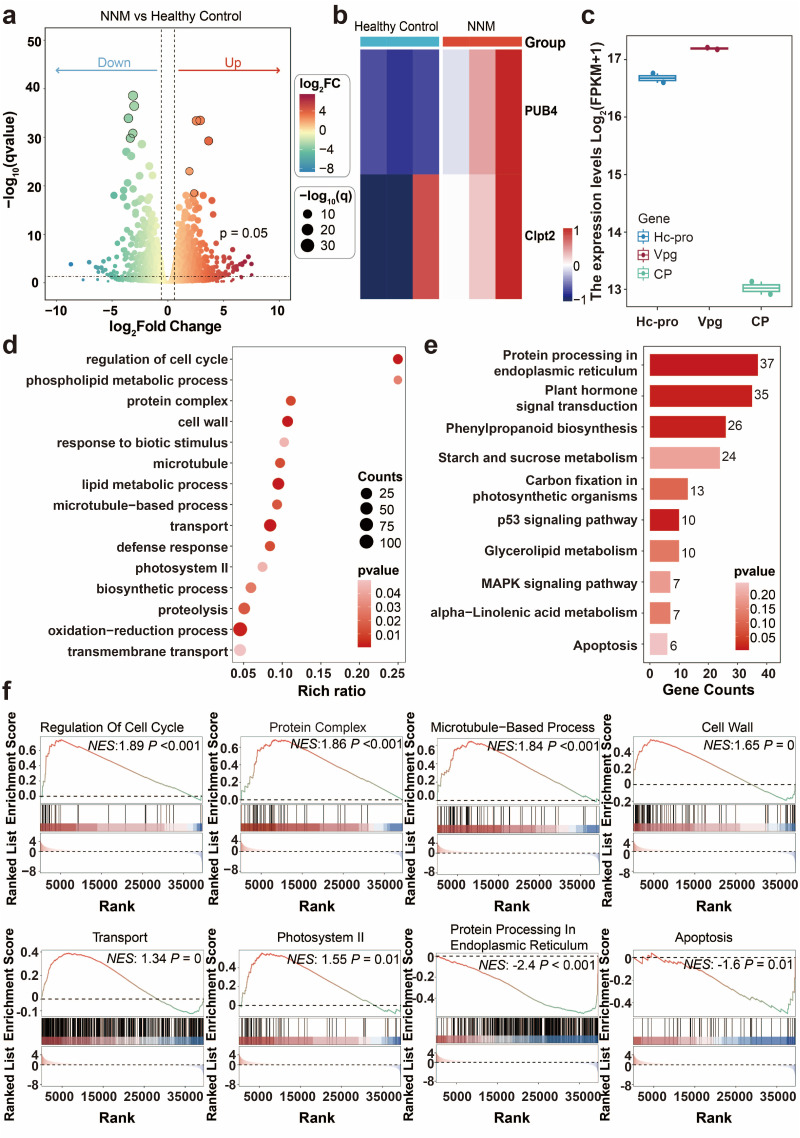
Bioinformatic mining and rational selection of host–virus interaction biomarkers based on transcriptomic profiling. (a) Volcano plot illustrating the global gene expression differences between NNM and healthy controls. (b) Heatmap visualization of the expression patterns of PUB4 and Clpt2. (c) Expression abundance of the prioritized viral targets (HC-pro, Vpg, and CP). (d) GO enrichment analysis of the identified DEGs. (e) KEGG pathway enrichment analysis showing the top enriched pathways. (f) GSEA plots revealing the directionality of pathway regulation. Pathways with a Normalized Enrichment Score (NES) > 1 indicate positive regulation, whereas those with an NES < −1 are identified as suppression.

To construct a biologically meaningful interactome, we moved beyond empirical marker selection and employed a data-driven transcriptomic strategy to prioritize gene targets critical for the host–pathogen interaction. Leveraging the transcriptomics data and a public RNA-seq dataset of *N. benthamiana* under NNM treatment *versus* healthy controls, we first mapped the global landscape of transcriptional reprogramming. Differential expression analysis (Fig. S9a) identified 1826 dysregulated genes (1203 upregulated and 623 downregulated), reflecting a profound host response to antiviral intervention. The volcano plot (Fig. 4a) visualizes this broad transcriptomic shift, applying a stringent filtering criterion (*P*-value < 0.05 and |log_2_ FC| > 1). From this high-dimensional dataset (Table S1), we prioritized host factors based on two criteria: significant differential expression under treatment and functional relevance to immune or metabolic homeostasis.^35,36^ Heatmap analysis (Fig. 4b) revealed the distinct upregulation of Plant U-box protein 4 (PUB4) and Caseinolytic protease T2 (Clpt2) in the NNM-treated group.

Consequently, PUB4 was selected as a representative immune hub due to its pivotal role in the ubiquitin–proteasome system (UPS),^37,38^ while Clpt2 was chosen as a metabolic sentinel reflecting plastid proteostasis and stress adaptation.^39^ In parallel, we scrutinized the viral transcriptome to select optimal pathogen markers (Fig. S11b). Transcriptomic profiling of the viral genome revealed distinct expression tiers. Specifically, viral genome-linked protein (VPg) and Helper component-proteinase (HC-pro) exhibited the highest expression stability, rendering them as well-suited targets for high-sensitivity detection compared to fluctuating downstream genes. Although Coat protein (CP) displayed lower relative abundance, it was retained as a critical node representing structural assembly and late-stage viral load (Fig. 4c). Therefore, we further selected Vpg (replication),^40^ Hc-pro (silencing suppression)^41^ and CP (assembly)^40^ as the targets to ensure the viral panel can capture the complete functional lifecycle of the pathogen.

To validate that these five selected markers are not isolated actors but proxies for broader systemic shifts, we performed functional enrichment analysis. Gene Ontology (GO) analysis (Fig. 4d) revealed that the differentially expressed genes are heavily clustered in cell cycle regulation, phospholipid metabolism, and defense responses. Consistently, Kyoto Encyclopedia of Genes and Genomes (KEGG) pathway analysis (Fig. 4e) identified significant enrichment in “Protein processing in the endoplasmic reticulum” and “Plant hormone signal transduction”, underscoring the centrality of protein quality control and signaling in the recovery process. Furthermore, Gene Set Enrichment Analysis (GSEA) resolved the directionality of these shifts (Fig. 4f). Revealing a coordinated activation of cell wall and microtubule-based processes (NES > 1.6) and a concomitant suppression of apoptosis and ER stress pathways (NES < −1.6) under treatment.

Importantly, the selected host biomarkers were functionally embedded within these key pathways: PUB4 serves as a gatekeeper for cell cycle and protein turnover, while Clpt2 regulates the metabolic flux essential for cell wall and plastid maintenance. By combining these system-embedded host markers with the functional viral triad (Vpg/Hc-pro/CP), we established a rationally designed, five-gene panel capable of mapping the topological evolution of the host–virus interactome with high biological fidelity.

### Highly specific and sensitive profiling of multiple DNA targets

**Fig. 5:**
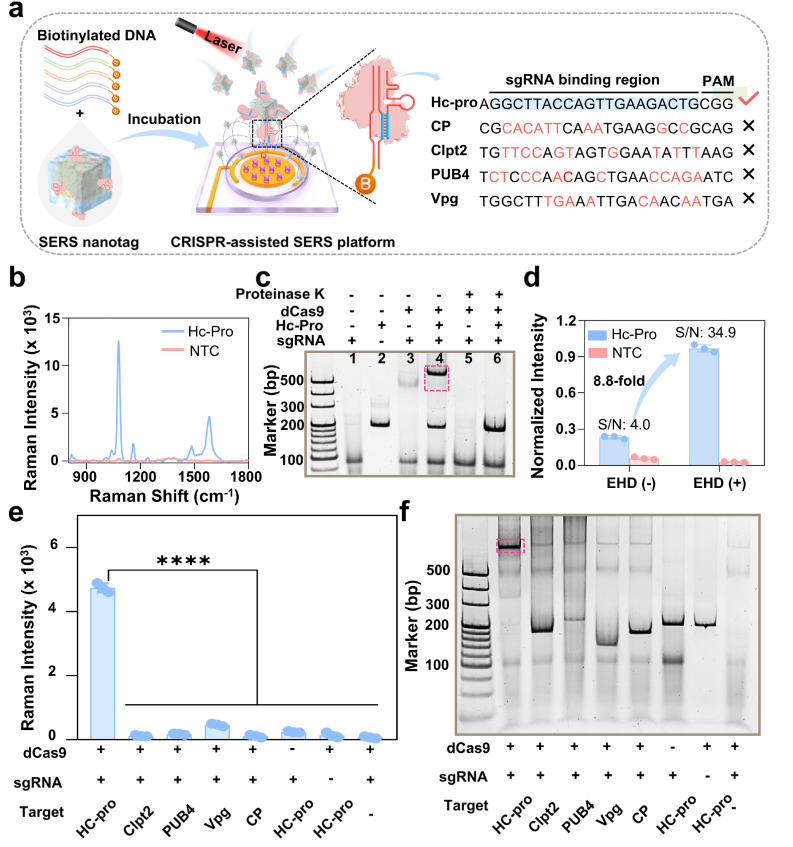
Performance evaluation of the multiplexed CRISPR-assisted SERS platform. (a) Schematic illustration of the Hc-Pro recognition through designed sgRNA within the CRISPR-assisted SERS platform. (b) Feasibility assessment of the CRISPR-assisted SERS platform for the detection of HC-Pro. (c) PAGE analysis for the recognition of Hc-Pro under different controls. (d) SERS signal intensity for Hc-Pro detection in the presence and absence of EHD. (e) Evaluation of the specificity of the CRISPR-assisted SERS platform for detecting Hc-Pro under various control conditions. Error bars represent mean ± sd (*n* = 3). (f) The corresponding PAGE analysis validating the specificity results shown in (e).

The working principle of our CRISPR-assisted SERS platform for multiplexed DNA profiling is schematically illustrated in Fig. 5a. To ensure high-fidelity target identification, we employed a rigorous computational strategy to engineer single-guide RNAs (sgRNAs) with single-nucleotide discrimination power (Table S2). This design maximized the thermodynamic penalty for off-target binding, particularly in the “seed region” proximal to the PAM site (Fig. S12–S15), ensuring that dCas9 binding occurred strictly in the presence of the correct target sequence.^31,42^ These optimized sgRNAs were complexed with dCas9 to form ribonucleoproteins (RNPs) and then conjugated to the isomeric Raman reporter-labeled Cu_2_O/CuO@Au@Ag nanostructures, which produced the functional SERS nanotags. Upon capture of biotinylated target DNAs on the gold microelectrodes, these specific SERS nanotags identified the targets and generated distinct spectral fingerprints. Specifically, the five targets were assigned to the following unique Raman channels: PUB4 (*m*-MTP at 992 cm^−1^), Clpt2 (*o*-FTP at 1031 cm^−1^), Vpg (*o*-MTP at 1040 cm^−1^), Hc-Pro (*p*-FTP at 1071 cm^−1^), and CP (*p*-MTP at 1080 cm^−1^).

We first validated the feasibility of this sgRNA-guided recognition strategy using Hc-Pro as a model target, given its pivotal role in viral replication. As shown in Fig. 5b, the presence of the Hc-Pro gene triggered a sharp, intense SERS signal at 1071 cm^−1^, whereas the no-template control (NTC) yielded a negligible background, confirming the successful formation of the SERS-active sandwich complex. This binding interaction was further corroborated by an electrophoretic mobility shift assay (EMSA) (Fig. 5c). The co-incubation of target DNA with the dCas9/sgRNA complex resulted in a distinct, high-molecular-weight band (Lane 4) compared to the free components. Crucially, proteinase K digestion of this complex released the DNA and sgRNA, causing the reappearance of their respective lower-molecular-weight bands (Lane 6). This reversible shift conclusively proved that the detection mechanism was driven by the specific, protein-mediated binding of the dCas9–RNP complex. We successfully verified this mechanism for all five targets (Fig. S16).

To maximize the analytical performance of the CRISPR-assisted SERS platform, we systematically optimized key experimental parameters, including the elemental composition of the Cu_2_O/CuO@Au@Ag nanostructures (Fig. S17), sgRNA recognition efficiency (Fig. S18), RNP complex, streptavidin concentration (Fig. S19), and the EHD parameters (*i.e.*, amplitude and frequency) (Fig. S20). In addition, a critical innovation in our platform was the integration of EHD to overcome diffusion limits. As quantified in Fig. 5d, the application of an EHD created a convective force that efficiently concentrated targets onto the sensor surface and selectively removed nonspecific binding events. This resulted in an 8.8-fold enhancement of the normalized Raman intensity compared to passive diffusion, boosting the signal-to-noise (S/N) ratio from 4.0 to 34.9.

We next demonstrated the multiplexing capability and orthogonality of the CRISPR-assisted SERS platform. When detecting five target mixtures, the SERS spectrum clearly resolved five distinct peaks corresponding to five DNA targets (Fig. S21). Furthermore, we rigorously evaluated detection specificity by challenging the Hc-Pro-targeting SERS nanotags with non-target genes (Vpg, CP, PUB4, and Clpt2). As shown in Fig. 5e, a robust signal was generated only in the presence of the complementary Hc-pro target, while mismatch targets produced signals indistinguishable from the blank. This high specificity was further confirmed by native PAGE analysis (Fig. 5f), where a clear band shift was observed exclusively in the lane containing Hc-Pro and its corresponding RNP. This confirmed that the dCas9-mediated recognition effectively eliminated the cross-reactivity often seen in traditional hybridization assays. We successfully verified this specificity for Vpg, CP, PUB4, and Clpt2 targets (Fig. S22).

Finally, to validate the utility of the CRISPR-assisted SERS platform for early-stage infection monitoring, we evaluated its sensitivity by measuring serial dilutions of the five targets. The platform exhibited a wide dynamic range from 10 pM to 10 nM, with clear concentration-dependent signal increases (Fig. S23).

Linear regression analysis yielded excellent correlation coefficients with *R*^2^ > 0.98. Based on three times the standard deviation of blank signals, the limits of detection (LODs) were calculated to be 2.5 pM (Hc-pro), 0.43 pM (Vpg), 6.4 pM (CP), 2.0 pM (PUB4), and 3.6 pM (Clpt2). These LOD values were highly competitive with previously reported CRISPR-based diagnostic methods (Table S3), underscoring the potential of this CRISPR-assisted SERS platform for the precise, quantitative profiling of low-abundance viral and host markers in complex plant lysates.

### Longitudinal dissection of the *N. benthamiana*–PVY interactome

**Fig. 6:**
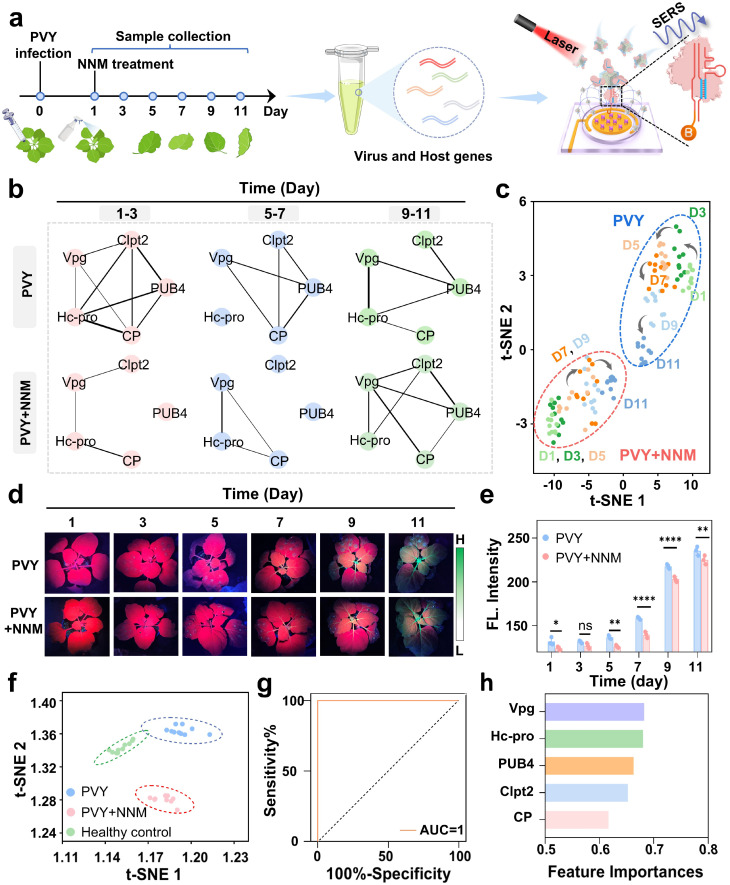
Integrated CRISPR-assisted SERS platform profiling viral–host gene dynamics in PVY-infected *N. benthamiana* under NNM treatment. (a) Schematic illustration of the SERS assay for dynamic monitoring of PVY infection and NNM treatment. (b) Dynamic correlation networks of five genes in PVY and PVY + NNM groups at different time points (Days 1–3, 5–7, and 9–11), with edge thickness proportional to the correlation weight. (c) t-SNE plots illustrating the temporal evolution trajectories of the infection process in PVY and PVY + NNM groups from Day 1 to Day 11. (d) Fluorescence images of *N. benthamiana* after inoculation with PVY-GFP for 1–11 days. (e) Semiquantitative fluorescence analysis of green fluorescence images in (d). (f) t-SNE scatter plot of Control, PVY, and PVY + NNM samples on Day 1 based on the five target genes. (g) ROC curve for the PVY-infected group and the healthy control group on Day 1. (h) Contribution rank of each gene in order of importance, as determined by the Logistic Regression algorithm.

Having validated the analytical performance of the CRISPR-assisted SERS platform, we deployed it to decipher the real-time molecular dialogue between *N. benthamiana* and PVY (Fig. 6a and S24). Rather than merely tracking viral load, our objective was to map the topological evolution of the host–pathogen interactome, thereby elucidating the distinct mechanisms of viral invasion, stabilization, and therapeutic escape.

We first constructed time-resolved interaction networks for both untreated (PVY) and treated (PVY + NNM) groups and divided into three critical phases (Days 1–3, 5–7, and 9–11) (Fig. 6b). In the untreated PVY group, the network evolved from chaotic invasion to streamlined dominance. The early phase (Day 1–3) exhibited a hyper-connected mesh where viral factors (Vpg, Hc-pro, CP) indiscriminately engaged host markers, reflecting the metabolic storm of initial host hijacking. By the late phase (Day 9–11), the network crystallized into a specific regulatory triangle comprising Vpg, Hc-pro, and PUB4, with CP and Clpt2 relegated to terminal nodes. This topology suggested that chronic, stable infection relied on a highly regulated architecture where viral replication (Vpg) and silencing suppression (Hc-pro) dually clamped the host immune hub (PUB4), while structural assembly (CP) was topologically compartmentalized to maximize efficiency.

In sharp contrast, the NNM-treated group showed a trajectory of blockade followed by chaotic relapse. During the therapeutic window (Day 1–7), the network remained sparse, characterized by the topological isolation of PUB4 and Clpt2 from viral nodes. This confirmed that NNM functioned as a molecular circuit breaker, preventing the virus from co-opting the host immune machinery. However, the late phase (Day 9–11) witnessed a dramatic topological explosion. Unlike the streamlined triangle seen in the stable untreated infection, the treated group relapsed into a complex, disorganized mesh where Vpg acted as a super-hub connecting directly to all nodes. Biologically, this signified that the high viral load at Day 11 was not a continuation of chronic infection, but an acute viral escape event where the virus frantically re-engaged all available host factors to overcome the drug blockade.

These topological insights offered a new paradigm for antiviral drug development. Although conventional screening targets viral enzymes, our data suggested that preventing resistance required targeting network topology. Specifically, drugs should be designed to disrupt the persistent Vpg–PUB4 interface, which appeared to be the key to stable infection. Furthermore, to prevent the chaotic relapse observed here, combinatorial therapies could be employed to specifically block the Vpg–CP interaction, a connection that was unique to the acute escape phase and absent in stable infection.

We further visualized these distinct evolutionary trajectories using t-distributed Stochastic Neighbor Embedding (t-SNE) (Fig. 6c). The analysis revealed a clear spatial divergence. The PVY group followed a linear progression from Day 1 to 11, consistent with a maturing infection. Conversely, the PVY + NNM group formed a tight cluster during the effective treatment window (Day 1–5), spatially separated from the infection trajectory. However, from Day 7 to 11, the treated samples migrated toward a distinct region, corroborating the network finding that relapse is a biological state distinct from both health and chronic infection.

This molecular monitoring was cross-validated by phenotypic analysis using Green Fluorescent Protein (GFP)-tagged PVY (Fig. 6d and e).^43–47^ While fluorescence imaging confirmed that NNM delayed viral accumulation, it showed a resurgence of signal by Day 11 that was statistically indistinguishable from the untreated group. Crucially, while fluorescence suggested the two groups were identical at Day 11, our SERS interactome analysis revealed they were mechanistically distinct (stable *vs.* chaotic). This underscores the limitation of simple viral load monitoring and the necessity of network profiling to assess the true durability of therapeutic response.

Finally, we explored the clinical utility of this five-gene panel for precise early diagnosis and stratification. Beyond simple detection, a critical challenge in agriculture is distinguishing between healthy plants, infected plants, and those undergoing effective treatment. As shown in Fig. 6f, the t-SNE analysis of the Day 1 spectral data successfully resolved the samples into three distinct, non-overlapping clusters corresponding to the healthy control, PVY-infected, and PVY + NNM groups. This proved that the multi-gene fingerprint captured the subtle molecular shifts induced by drug treatment immediately upon administration, a nuance invisible to standard methods. Leveraging this high-dimensional resolution, we trained three supervised machine learning algorithms (Logistic Regression (LR), Random Forest (RF), and Support Vector Machine (SVM)) to classify infection status (Fig. 6g and S25). Remarkably, all algorithms achieved an Area Under the Curve (AUC) of 1.0 as early as Day 1 post-infection. Feature importance analysis (Fig. 6h) identified Vpg and Hc-pro as the primary discriminators in this early phase, validating their roles as the drivers of initial invasion. These results, corroborated by qRT-PCR (Fig. S26), confirm that our platform enabled not just early detection, but the immediate stratification of therapeutic response, allowing for proactive and precision-guided agricultural management well before the appearance of phenotypic symptoms.

## Conclusions

Current paradigms in plant health management rely predominantly on the binary detection of pathogen presence, typically quantified by viral load. However, our results demonstrated that viral load was a lagging indicator that often failed to reflect the true physiological status of the host. A striking example from our study was the comparison at Day 11. For the *N. benthamiana* infected with PVY, both untreated and treated groups exhibited statistically identical high viral loads (fluorescence/PCR), yet they were biologically distinct. The untreated group had stabilized into a regulatory triangle topology, whereas the treated group exhibited a chaotic Vpg-centralized network. Relying on viral load alone would misclassify these distinct states (chronic stability *versus* acute failure), which potentially led to ineffective overtreatment of reservoir plants or the neglect of active super-spreaders. This underscored the necessity of transitioning from pathogen detection to interactome profiling, where the topology of the host–virus network served as the primary diagnostic metric.

To enable the high-dimensional data acquisition required for such network construction, we overcame the spectral limitations of traditional SERS through the *ab initio* design of an isomeric Raman reporter library. Unlike the random screening of commercial dyes, our molecular engineering approach utilized positional isomerism and electronic tuning to create a palette of 24 distinct reporters with predictable spectral shifts. This rational design strategy offered a powerful tool to resolve five targets simultaneously. Importantly, this coding capacity was scalable due to the narrow linewidths of our isomeric library, which theoretically supported even higher-order multiplexing and thus paved the way for comprehensive transcriptomic profiling on a single plasmonic chip.

Furthermore, the reliability of a biological network depended entirely on the stoichiometric accuracy of the input data. Traditional DNA hybridization was prone to thermodynamic bias, where GC-rich targets capture more efficiently than AT-rich ones, distorting the calculated correlation coefficients. By integrating CRISPR-dCas9, we replaced variable thermodynamic binding with uniform enzymatic recognition. The dCas9 system functions as an isothermal molecular search engine, resolving secondary structures and binding all five targets with kinetically comparable efficiency. This ensured that the signal intensity ratios in our SERS spectra were true reflections of biological abundance, not artifacts of probe folding. This integration of CRISPR specificity with SERS sensitivity represented a methodological evolution, offering a robust alternative to PCR for multiplexed quantification in complex plant lysates.

Biologically, our longitudinal interactome analysis offered specific, actionable insights for antiviral therapy. We identified that the Vpg–PUB4 interaction was the key to the infection process. In stable infection, the virus relied on Vpg to clamp the host immune regulator PUB4. In successful treatment, this link however was severed. Consequently, future drug discovery efforts should prioritize small molecules or peptides that specifically disrupt the Vpg–PUB4 protein–protein interface. Additionally, the emergence of the Vpg Super-Hub (where Vpg connected directly to CP and Clpt2) indicated drug resistance and viral escape. Therefore, screening for this topological signature could allow for the rapid identification of resistant strains weeks before phenotypic failure became apparent.

In summary, this work established a new framework for precision agriculture through network-based diagnostics. Our CRISPR-assisted SERS platform progressed from the paradigm of static pathogen detection to elucidate the dynamic functional state of the viral infection. By deciphering the dynamic interactome between *N. benthamiana* and PVY, we have provided a blueprint for differentiating between invasion, suppression, and relapse based on network topology. This approach not only can facilitate earlier and more accurate diagnosis but also guide the rational design of next-generation antivirals that function by dismantling the molecular infrastructure of viral pathogenesis. As climate change accelerates the spread of plant vectors, such systems-level monitoring tools will be indispensable for ensuring global food security.

With ongoing advances in plasmonic nanomaterials, molecular coding strategies, and CRISPR-assisted recognition technologies, SERS platforms are expected to evolve toward real-time, high-dimensional analysis of plant transcriptomes, proteomes, metabolomes, and signaling networks under both biotic and abiotic stresses. In particular, the integration of portable SERS devices with artificial intelligence and network biology may enable precision in-field diagnostics, early stress forecasting, and dynamic surveillance of plant immune responses throughout the course of infection. We envision SERS technology can aid in next-generation precision agriculture and sustainable crop protection.

## Author contributions

Y. S., J. L. and X. L. designed the research. Y. D. and N. C. synthesized and characterized the nanostructures, performed the SERS measurements, and analyzed the data. G. W. synthesized the Raman reporter. X. C. and T. Z. collected the plant samples. J. L. and T. X. analyzed the RNA sequencing. J. L., Y. D. and X. Z. performed the data analysis of the SERS spectra using the AI system. All authors wrote and revised the manuscript.

## Conflicts of interest

The authors declare no competing interests.

## Supplementary Material

SC-OLF-D6SC03368G-s001

## Data Availability

The data that support the findings of this work are available within the manuscript and its additional files, this paper's supplementary information (SI), and on GitHub (https://github.com/Xutainbing/RNA-seq). Supplementary information (SI) is available. See DOI: https://doi.org/10.1039/d6sc03368g.

## References

[cit1] Yang X., Li Y., Wang A. (2021). Annu. Rev. Phytopathol..

[cit2] Ge L., Zhou X., Li F. (2024). Trends Plant Sci..

[cit3] Jones J. D., Dangl J. L. (2006). Nature.

[cit4] Nagy P. D., Pogany J. (2011). Nat. Rev. Microbiol..

[cit5] Carthew R. W., Sontheimer E. J. (2009). Cell.

[cit6] Tan E. X., Leong Y. X., Lim S. H., Chng M. W. K., Phang I. Y., Ling X. Y. (2025). Nat. Commun..

[cit7] Pumplin N., Voinnet O. (2013). Nat. Rev. Microbiol..

[cit8] Ling Q., Broad W., Trösch R., Töpel M., Demiral Sert T., Lymperopoulos P., Baldwin A., Jarvis R. P. (2019). Science.

[cit9] Hao Z., Xu X., Wang G. L., Wang R., Ning Y. (2025). Nat. Plants.

[cit10] Zhang Y., Gao Y., He W., Wang Y., Qian Y., Zhou X., Wu J. (2020). J. Integr. Agric..

[cit11] Ahsan H. (2022). Comp. Clin. Pathol..

[cit12] He X., Zheng Y., Tian C., Wen T., Yang T., Yu J., Fang X., Fan C., Liu J., Yu L. (2023). Phytomedicine.

[cit13] Raza M., Hussain Z., Abbas F., Ashraf M. A., Imene H. H., Riaz T. (2024). Hosts Viruses.

[cit14] Huang Y.-Y., Li W.-H., Lee K.-Y., Tsai W.-S., Tsai C.-W. (2025). Agriculture.

[cit15] Rashid S., Wani S., Iralu N., Ali G., Hamid A. (2025). Indian. J. Microbiol..

[cit16] Bi X., Qian X., Xue B., Zhang M., Liu S., Chen H., Jin C., Tang H., Ye J. (2025). Chem.

[cit17] Zhao W., Ye H., Li X., Liu J., Zhou X., Chen X., Xue Z., Yang Z., Wang T. (2023). Chem.

[cit18] Fabris L. (2023). Chem.

[cit19] Hu P., Yang H., Si R., Wei B., Wang X., Xu Z., Yang X., Guo T., Gebauer R., Teobaldi G., Liu L.-M., Wang Z., Guo L. (2024). Chem.

[cit20] Han X. X., Rodriguez R. S., Haynes C. L., Ozaki Y., Zhao B. (2022). Nat. Rev. Methods Primers.

[cit21] Mu M., Chen J., Xue X., Yang Y., Qi R., Wang Y., Liu D., Shang L., Jiang W., Shao X., Chen Z.-j., Zhao B., Song W. (2025). Chem. Sci..

[cit22] Jo Y., Kim Y., Kang R., Lee S., Nguyen D. D., Park S., Lee D., Han J. W., Mook-Jung I., Lee L. P., Park J. C., Kim I. (2025). Adv. Sci..

[cit23] Zhang Y., Li C., Carland R., Ye Z., Bell S. E. J., Xu Y. (2025). Adv. Sci..

[cit24] Zhang X., Li C., Yan M., Wang Y., Dou Y., Zhang T., Zheng S., Wang H., Li J., Sun Y. (2026). J. Am. Chem. Soc..

[cit25] Dwivedi A., Dellith J., Makarova A., El-Mashtoly S. F., Popp J., Sivakov V., Cialla-May D. (2026). Adv. Sci..

[cit26] Tan E. X., Leong S. X., Liew W. A., Phang I. Y., Ng J. Y., Tan N. S., Lee Y. H., Ling X. Y. (2024). Nat. Commun..

[cit27] Fu G., Li J., Zhang Q., Lv C., Zhang Z., Wang X., Wu R., Chen L. (2025). Chem. Sci..

[cit28] Koo K. M., Wee E. J. H., Mainwaring P. N., Wang Y., Trau M. (2016). Small.

[cit29] Iaccarino N., Cheng M., Qiu D., Pagano B., Amato J., Di Porzio A., Zhou J., Randazzo A., Mergny J. L. (2021). Angew. Chem., Int. Ed..

[cit30] Xu G., Bao Y., Zhang Y., Xiang X., Luo H., Guo X. (2024). Anal. Chem..

[cit31] Zhang B., Zhang P., Wang H., Wang X., Hu Z., Wang F., Li Z. (2025). ACS Nano.

[cit32] Su A., Liu Y., Cao X., Zhao J., Xu W., Liang C., Li P., Xu S. (2023). Anal. Chem..

[cit33] Feng E., Zheng T., He X., Chen J., Gu Q., He X., Hu F., Li J., Tian Y. (2023). Angew. Chem., Int. Ed..

[cit34] Liu Q., Feng E., Li S., Zhu Y., He X., Xu X., Zheng T., Tian Y. (2026). Angew. Chem., Int. Ed..

[cit35] Bu F., Shen X., Zhan H., Wang D., Min L., Song Y., Wang S. (2025). J. Am. Chem. Soc..

[cit36] Zhang D., Peng K., Xu H., Chen Y., Wang J. (2025). Adv. Sci..

[cit37] Wu Z., Huang M., Jiang J., Zhang C., Hao G., Chen M., Li Q. X., Jia M., Liu J., Li X. (2024). J. Agric. Food Chem..

[cit38] Wang Y., Wu Y., Zhong H., Chen S., Wong K. B., Xia Y. (2022). New Phytol..

[cit39] Kim J., Kimber M. S., Nishimura K., Friso G., Schultz L., Ponnala L., van Wijk K. J. (2015). Plant Cell.

[cit40] Andreja Kežar L. K., Polák M., Nováček J., Gutiérrez-Aguirre I., Tušek Žnidarič M., Coll A., Stare K., Gruden K., Ravnikar M., Pahovnik D., Žagar E., Merzel F., Anderluh G., Podobnik M. (2019). Sci. Adv..

[cit41] Valli A., Gallo A., Calvo M., Pérez J. d. J., García J. A., Simon A. (2014). J. Virol..

[cit42] Yang W., Restrepo-Perez L., Bengtson M., Heerema S. J., Birnie A., van der Torre J., Dekker C. (2018). Nano Lett..

[cit43] Gao S., Zhang Y., Cui K., Zhang S., Qiu Y., Liao Y., Wang H., Yu S., Ma L., Chen H., Ji M., Fang X., Lu W., Xiao Z. (2025). Nat. Biotechnol..

[cit44] Li C., Tu L., Xu Y., Li M., Du J., Stang P. J., Sun Y., Sun Y. (2024). Angew. Chem., Int. Ed..

[cit45] Pang Y., Li Q., Wang J., Wang S., Sharma A., Xu Y., Hu H., Li J., Liu S., Sun Y. (2024). Angew. Chem., Int. Ed..

[cit46] Tu L., Li C., Ding Q., Sharma A., Li M., Li J., Kim J. S., Sun Y. (2024). J. Am. Chem. Soc..

[cit47] Xu Y., Pang Y., Luo L., Sharma A., Yang J., Li C., Liu S., Zhan J., Sun Y. (2024). Angew. Chem., Int. Ed..

