# Supplementary material for: High-dimensional CRISPR-SERS interactomics tracks the topological evolution of plant viral pathogenesis and therapeutic response
Source: Chem Sci. 2026 Jun 4;17(28):13976–87. doi: 10.1039/d6sc03368g (PMC13251604; doi:10.1039/d6sc03368g)
Supplement: SC-017-D6SC03368G-s001 [file SC-017-D6SC03368G-s001.pdf]

Supplementary Information (SI) for Chemical Science.
This journal is © The Royal Society of Chemistry 2026

Supporting Information

High-Dimensional CRISPR-SERS Interactomics Tracks the Topological Evolution of Plant Viral Pathogenesis and Therapeutic Response

You Dou,[a†] Gang Wu,[b†] Jun Li,[e†] Yuting Wang,[c] Nanke Chen,[a] Tianbing Xu,[c] Xiaobo Cai,[d] Tuotuo Zhang,[a] Xinwei Zhang,[a] Xiangyang Li[d]*, Junrong Li[a]* and Yao Sun[a,c,f]*

[a]*College of Chemistry, Central China Normal University, Wuhan 430079, China*

[b]*The Department of Radiology, Tongji Hospital of Tongji Medical College of Huazhong University of Science and Technology*

[c]*National Key Laboratory of Agricultural Microbiology, College of Biomedicine and Health, Huazhong Agricultural University, Wuhan 430070, China*

[d]*State Key Laboratory of Green Pesticide, Key Laboratory of Green Pesticide and Agricultural Bioengineering, Ministry of Education, Guizhou University, Guiyang 550025, China*

[e]*College of Chemistry, Huazhong Agricultural University, Wuhan, 430070, China*

[f]*Hubei Jiangxia Laboratory, Wuhan 430020, China*

†These authors contributed equally to this work.

*Corresponding authors (email: sunyaogbasp@ccnu.edu.cn; junrong.li@ccnu.edu.cn; xyli1@gzu.edu.cn)

Table of Contents

Experimental Section……………………………………………………………2

Supplementary Tables……………………………………………………………23

Supplementary Figures…………..…………………………………………….…27

1. Experimental Section

1.1. Materials

dCas9 enzyme, proteinase K and RNase inhibitors used in this study were purchased from Integrated DNA Technologies, Inc. (Shanghai, China). Biotin-primers, plasmid DNA and sgRNA were ordered from Sangon Biotech Co., Ltd. (Shanghai, China). FastPure Gel DNA Extraction Mini Kit, 2 × Taq Plus Master Mix II (Dye Plus) and HiScript® III RT SuperMix for qPCR (+ gDNA wiper) were purchased from Vazyme Biotech Co., Ltd. (Nanjing, China). RNAprep Pure Plant Plus Kit was supplied by Tiangen Biotech Co., Ltd. (Beijing, China). Realsafe, 4S Red Plus Nucleic Acid Stain and 20-500 bp dsDNA Marker were purchased by Real-Times Biotechnology Co., Ltd. (Beijing, China). Ammonium hydroxide ($NH_3•H_2O$) was ordered from Sinopharm Chemical Reagent Co., Ltd. (Tianjin, China). Copper II) sulfate pentahydrate ($CuSO_4•5H_2O$), streptavidin (SA), dopamine hydrochloride, silver nitrate ($AgNO_3$), chitosan and hydroxylamine hydrochloride ($NH_2OH•HCl$) were obtained from Aladdin Biochemical Technology (Shanghai, China). Bovine serum albumin (BSA), hydrogen tetrachloroaurate (III) trihydrate ($HAuCl_4•3H_2O$) and Tris(hydroxymethyl)aminomethane ascorbic acid (AA) were acquired from Sigma-Aldrich (St. Louis, MO). 30% PAA was bought from Servicebio Technology Co., Ltd. (Wuhan, China). N, N, N', N'-Tetramethylethylenediamine (TEMED) was purchased from Beyotime Biotechnology (Shanghai, China). β-Mercaptothanol was provided by Macklin Biochemical Co., Ltd. (Shanghai, China). All of the solutions used in this work were prepared in RNase-free water (Thermo Fisher Scientific).

1.2. Preparation of the $Cu_2O/CuO@Au@Ag$

The $Cu_2O/CuO@Au@Ag$ nanostructures were prepared through a sequential coating strategy in which Cu_2O/CuO nanocubes were first coated with a gold shell, subsequently modified with Raman reporter molecules, and finally encapsulated within a silver layer. The Cu_2O/CuO nanocubes were obtained via a kinetically controlled liquid-phase reduction route optimized for cubic morphology. Briefly, 35 mL of deionized water was transferred into a conical flask and equilibrated in a thermostatic water bath at 35 °C under continuous stirring (770 rpm) for 10 min. Thereafter, 400 μL of 0.1 M $CuSO_4$ solution was rapidly injected. After exactly 1 min, 2.6 mL of freshly prepared 1.0 M NaOH solution was introduced, resulting in the immediate formation of a turbid blue suspension due to $Cu(OH)_2$ precipitation. Subsequently, 2 mL of 0.2 M ascorbic acid was added as the reductant, during which the solution color changed rapidly from turbid blue to lemon yellow. The reaction was allowed to proceed for an additional 10 min at 35 °C under stirring to complete the reduction. For the growth of the gold shell, 1 mL of the as-prepared Cu_2O/CuO nanocubes was concentrated to 200 μL by centrifugation (800 × g, 10 min) and then incubated with 20 mM $KAuCl_4$ for 3 min to enable electrostatic adsorption of Au^{3+} onto the nanocube surface. Subsequently, 15 μL of 20 mM hydroxylamine hydrochloride ($NH_2OH·HCl$) was added as a reducing agent under gentle agitation (650 rpm, 25 °C, 10 min), leading to the formation of a conformal Au shell ($Cu_2O/CuO@Au$). For surface functionalization, a premixed solution containing Raman reporter molecules (5 μL) and chitosan (30 μL, 1 mg mL^{-1} in 5% acetic acid) was added to the Au-coated nanocubes (35 μL mixture per 200 μL

nanostructures). After incubation for 3 h, unbound reporters were removed by centrifugation (400 × g, 10 min), resulting in monolayer coverage conducive to high surface-enhanced Raman scattering (SERS) efficiency. The obtained Cu_2O/CuO@Au@Reporter nanostructures were further concentrated to 100 μL (400 × g, 10 min) and subjected to ammonia-mediated silver deposition. Sequential addition of 5 μL 10 mM $AgNO_3$, 3 μL $NH_3 \cdot H_2O$ to generate $[Ag(NH_3)_2]^+$ complexes, followed by 3 μL 50 mM AA under agitation (650 rpm, 25 °C, 20 min), yielded a compact silver shell, affording the final Cu_2O/CuO@Au@Reporter@Ag nanostructures.

1.3. Synthesis of Raman reporters

The Raman reporter molecules were synthesized according to a nucleophilic substitution protocol. In a dry 100 mL round-bottom flask, 4-iodoanisole (5.0 mmol, ~1.34 g), 1,2-ethanedithiol (10.0 mmol, ~0.94 mL), copper (II) sulfate pentahydrate (0.5 mmol, ~0.125 g), and a DMSO/H_2O mixed solvent (4:1, v/v; total volume 20 mL) were successively introduced under ambient atmosphere. After magnetic stirring was initiated, sodium hydroxide (10.0 mmol, ~0.4 g) was added portionwise, ensuring that the reaction temperature did not exceed 40 °C. The flask was then fitted with a reflux condenser, and the reaction mixture was heated in an oil bath at 80–90 °C with vigorous stirring for 12–24 h. The reaction progress was periodically monitored by thin-layer chromatography (TLC). Upon completion, the reaction mixture was allowed to cool to room temperature, diluted with deionized water (50 mL), and the pH was adjusted to neutrality using 1 M HCl. The aqueous phase was subsequently extracted with ethyl acetate (3 × 30 mL). The combined organic layers were dried over anhydrous sodium

sulfate, followed by solvent removal under reduced pressure using rotary evaporation to afford the crude product. Purification was achieved by silica-gel column chromatography employing a petroleum ether/ethyl acetate gradient, yielding p-iodothiophenol as a pale-yellow liquid.

1.4. DFT simulation of isomeric Raman reporters

The theoretical Raman spectra were calculated using the quantum chemistry program Gaussian 16. Geometry optimization, vibrational frequency analysis, and Raman spectral calculations were performed employing the B3LYP functional combined with the 6-311G(d) basis set. To facilitate direct comparison with experimental Raman spectra, the calculated Raman shifts were scaled by a frequency correction factor of 0.9614 for further calibration. The Raman activities obtained from Gaussian 16 calculations were converted to Raman intensities using the Multiwfn program, which was subsequently employed to plot the theoretical Raman spectra of the compound.

1.5. Preparation of dCas9–sgRNA RNP complexes

All sgRNA sequences employed in this work were designed using the Benchling platform and are fully provided in Table S3 of the Supporting Information. Prior to complex assembly, the synthetic sgRNA samples were subjected to thermal denaturation at 95 °C for 5 min to disrupt potential secondary structures and duplex formation. The dCas9–sgRNA RNP complexes were generated by stoichiometric assembly. Specifically, recombinant dCas9 protein (1.0 μL, 6.2 μM; catalytically inactive variant lacking both RuvC and HNH nuclease activities) was mixed with synthetic sgRNA (0.6 μL, 10 μM) in 8.4 μL of 1 × NEB Buffer 3.1 (10 mM Tris-HCl,

50 mM NaCl, 1 mM DTT, pH 7.9). The resulting mixture was incubated at 37 °C for 30 min to allow formation of the RNP complex.

1.6. Immobilization of dCas9-sgRNA on the Cu_2O/CuO@Au@Reporter@Ag surface

The Cu_2O/CuO @Au@Reporter@Ag NPs were first dispersed in 100 μL of deionized water by sequential ultrasonication for 30 s and vortex mixing for 30 s, thereby ensuring colloidal uniformity. Dopamine solution (0.2 mg mL^{-1} in H_2O) together with 50 mM Tris–HCl buffer (pH 8.5) was then added to initiate oxidative polymerization, resulting in the formation of a polydopamine (PDA) layer that adhered strongly to the NP surface via covalent interactions and π–π stacking. The PDA-coated nanostructures (denoted as Cu_2O/CuO@Au@Reporter@Ag@DA) were collected by centrifugation (400 × g, 10 min) and subsequently incubated with 3 μL of CRISPR/dCas9 RNP complex in 10 mM Tris–HCl buffer (pH 8.5) under shaking conditions (650 rpm, 25 °C, 1 h) to achieve surface conjugation. To suppress nonspecific binding, streptavidin solution (1 mg mL^{-1} in PBS) was applied for 30 min under shaking incubation. After centrifugation (400 × g, 10 min), the resulting conjugates were resuspended in 20 μL of 0.1 × NEB Buffer. Artificial protein coronas targeting different genes were obtained using the same procedure, with the corresponding sgRNA sequences replaced accordingly.

1.7. Collection of biotinylated DNA

Biotinylated double-stranded DNA (dsDNA) targets were obtained by polymerase chain reaction (PCR) amplification. The reactions were performed in 50 μL systems containing 25 μL of 2 × Taq PCR Master Mix, 2.0 μL each of 10 μM forward and

reverse primers, 1.0 μL of plasmid DNA template (10 ng μL^{-1}), and 20 μL of nuclease-free water. Thermal cycling was carried out using the following program: an initial denaturation step at 95 °C for 3 min; 34 amplification cycles consisting of 95 °C for 30 s, 55 °C for 20 s, and 72 °C for 10 s; followed by a final extension at 72 °C for 10 min and subsequent holding at 4 °C. The resulting PCR products were separated by electrophoresis on a 1% agarose gel containing 4S Green Plus nucleic acid stain and purified using the FastPure Gel DNA Extraction Mini Kit (Vazyme) according to the manufacturer's instructions. After purification, the amplicons were analyzed again by agarose gel electrophoresis to assess product quality and integrity.

1.8. CRISPR-assisted SERS detection of multiple target genes

Gold microelectrode surfaces were first functionalized by depositing 20 μL of SA (1 mg mL^{-1} in PBS, pH 7.4) at 25 °C for 1 h to generate biotin-binding interfaces. Excess SA was removed through three washing steps with PBS (20 μL each). The electrodes were subsequently passivated with 1% BSA in PBS at 25 °C for 1 h to minimize nonspecific adsorption. Biotinylated double-stranded DNA targets were then immobilized by incubation with the SA-modified electrodes in nuclease-free water for 40 min. Surface-bound DNA was probed using the SERS nanotags (Cu_2O/CuO@Au@Reporter@Ag@DA/RNP). A 20 μL suspension of the nanotags was applied to the electrodes and incubated for 30 min under an alternating electric field with a frequency of 1 kHz and an amplitude of 200 mV to promote electrohydrodynamic localization. After incubation, unbound SERS probes were removed by three rinses with 1% BSA in PBS, and Raman spectra were subsequently

recorded.

1.9. PAGE analysis of RNP

For PAGE analysis of the RNP complexes, reactions were prepared in 10 μL volumes. Each mixture contained 50 nM sample together with 1.0 μL of dCas9 protein (6.2 μM), 0.6 μL of sgRNA (10 μM), 1 μL of NEBuffer 3.1 (NEB), and nuclease-free water to the final volume. The assembled mixtures were incubated at 37 °C for 30 min to allow RNP formation. Subsequently, 1.5 μL of proteinase K (NEB) was added, and the samples were further incubated at 60 °C for 10 min to digest and remove the dCas9 protein. Following enzymatic treatment, 6 × DNA loading buffer was added, and the resulting products were subjected to electrophoresis on 6% native polyacrylamide gels for separation and analysis.

1.10. PVY infection assay

An Agrobacterium tumefaciens strain carrying the PVY–GFP expression vector was cultured in Luria–Bertani (LB) medium supplemented with kanamycin (50 μg mL^{-1}) and rifampicin (25 μg mL^{-1}) at 28 °C for 48 h. The bacterial cultures were then harvested by centrifugation at 5000 rpm for 5 min and washed three times with PBS. The optical density at 600 nm of the bacterial suspension in PBS was determined using a UV–Vis spectrophotometer. Bacteria were resuspended in infiltration buffer (10 mM $MgCl_2$, 10 mM MES, 100 μM acetosyringone, pH 5.6) to achieve a final OD_{600} of 1.5 and incubated at room temperature for 3 h before infiltration. The prepared suspension was subsequently introduced into *N.* benthamiana plants at the three-leaf stage via a 1 mL syringe. Twenty-four hours after infiltration, leaves were treated with

Ningnanmycin (NNM) or a mock solution (1% DMSO with Tween), with three biological replicates assigned per treatment group. GFP fluorescence images were recorded under 365 nm excitation at 1, 3, 5, 7, 9, and 11 days post-treatment. Concurrently, leaf samples from both PVY-infected control and treated plants were collected and stored at −80 °C for subsequent SERS analysis

1.11. Plant DNA extraction and Real-Time quantitative PCR

Total RNA was extracted from PVY-infected leaves using the RNAprep Pure Plant Plus Kit, and its purity was evaluated by measuring the OD_{260}/OD_{280} ratio. Reverse transcription was subsequently performed at 37 °C for 15 min, followed by brief heating at 80 °C for 5 s to terminate the reaction. The resulting cDNA served as the template for PCR amplification to generate the target double-stranded DNA (dsDNA). For PCR, a 50 μL reaction mixture was assembled containing 25 μL of 2 × Taq PCR Master Mix, 2.0 μL each of forward and reverse primers (10 μM), 2.0 μL of cDNA, and 19 μL of nuclease-free water. Thermal cycling was conducted with an initial denaturation at 95 °C for 3 min, followed by 34 amplification cycles comprising 95 °C for 30 s, 55 °C for 20 s, and 72 °C for 10 s. A final extension at 72 °C for 10 min was applied, after which the reactions were maintained at 4 °C. Amplified products were purified using the FastPure Gel DNA Extraction Mini Kit (Vazyme). In addition, quantitative real-time PCR (qPCR) was performed using a portion of the cDNA. Each 12.5 μL reaction contained 10 μL of PCR Master Mix and 2.5 μL of cDNA. The thermal program consisted of 50 °C for 15 min, 95 °C for 30 s, followed by 40 cycles of 95 °C for 3 s and 60 °C for 40 s. Fluorescence signals were recorded at the end of each cycle using a

CFX96 Touch Deep Well Real-Time PCR Detection System.

1.12. RNA sequencing

RNA sequencing was performed by Shanghai OE BioTech Co., Ltd. on the Illumina HiSeq X Ten platform. Raw sequencing data underwent quality control and adapter sequence removal using Trimmomatic[1] software (version 0.36) to obtain high-quality clean reads. Subsequently, the clean reads were aligned to the reference genome of the corresponding species using hisat2[2] software (version 2.2.1.0) under default parameters. Gene expression levels were quantified using Cufflinks[3] software (version 2.2.1) and expressed as fragment per kilobase of transcript per million mapped reads (FPKM) values. The reference genome version for Potato virus Y (PVY) gene quantification was GCF_002987625.1_ASM298762v1[4], while the reference genome version for *N*. benthamiana gene quantification was Niben_v1.0.1[5].

1.13. Principal component analysis

Based on the gene expression matrix encompassing all samples, principal component analysis (PCA) was performed on the normalized expression data using the R package tinyarray (version 2.3.1). By visualizing the first two principal components, the overall separation between different treatment groups and the consistency within replicate samples were evaluated.

1.14. Differential expression and enrichment analysis

Differential expression analysis was performed using the R package limma (version 3.54.2) to compare gene expression differences between the NNM group and the MOCK group. Significantly differentially expressed genes (DEGs) were identified

using a threshold of $p < 0.05$ and $|\log_2 FoldChange| > 1$. Results were visualized using the ggplot2 package (version 3.5.1). Subsequently, the R package clusterProfiler[6] (version 4.15.0) was employed to perform Gene Ontology (GO) and Kyoto Encyclopedia of Genes and Genomes (KEGG) pathway enrichment analysis on all DEGs (including both up- and down-regulated genes). Based on the $\log_2$FoldChange values of each gene in the differential analysis results, gene set enrichment analysis (GSEA) was performed using the clusterProfiler package. The gene sets were sourced from the GO and KEGG databases, with a significance threshold set at an adjusted p-value (p.adj) < 0.25.

1. Trimmomatic: a flexible trimmer for Illumina sequence data

2. HISAT: a fast spliced aligner with low memory requirements

3. Transcript assembly and quantification by RNA-Seq reveals unannotated transcripts and isoform switching during cell differentiation

4. Infectious in vivo and in vitro transcripts from a full-length cDNA clone of PVY-N605, a Swiss necrotic isolate of potato virus Y

5. The Sol Genomics Network (SGN)--from genotype to phenotype to breeding

6. Using clusterProfiler to characterise Multi-Omics Data

1.15. Data processing and machine learning analysis

All analyses were conducted using Python (v3.13). Data manipulation was performed using Pandas and NumPy. Machine learning and statistical modeling were executed using Scikit-learn. Network topology analysis was performed using NetworkX.

Raw SERS spectral data were first preprocessed to ensure quantitative accuracy. To

account for potential variations in SERS nanotag concentration or surface enhancement efficiency, the intensity of each target biomarker was normalized against the intrinsic Raman intensity of its specific SERS nanotag, yielding a normalized intensity ratio. Prior to machine learning analysis, the dataset was standardized using Z-score normalization (zero mean and unit variance) to ensure equal contribution from all features.

To visualize the high-dimensional temporal evolution of the host-virus interaction, we employed t-SNE. The analysis was performed using the Scikit-learn library in Python. The perplexity parameter was dynamically adjusted based on sample size to balance local and global manifold preservation, and a fixed random state was utilized to ensure reproducibility. The resulting two-dimensional embeddings were plotted to illustrate the clustering patterns and trajectory divergence between Healthy, PVY-infected, and NNM-treated groups over time.

Host-virus interaction networks were constructed to visualize the topological evolution of infection. Pearson correlation matrices were calculated for the five target genes at each time phase. The interaction data was modeled as an undirected graph using the NetworkX library, where nodes represented specific genes and edges represented the correlation strength. To ensure robust visualization of significant interactions, edges were rendered only for correlations satisfying a significance threshold ($|r|>0.2$). Edge width was scaled proportionally to the correlation coefficient to reflect interaction strength.

To evaluate the diagnostic capability of the five-gene panel, we developed and

compared three supervised classification algorithms: LR, RF, and SVM with a linear kernel. To prevent overfitting and ensure statistical robustness, performance was evaluated using Stratified 5-Fold Cross-Validation. This ensured that the class distribution (infected vs healthy) remained consistent across all training and validation folds. Diagnostic accuracy was quantified using ROC curves and the AUC. The contribution of each biomarker to the diagnostic decision was extracted to interpret biological relevance. For LR and SVM, importance was derived from the absolute values of the model coefficients. For RF, importance was calculated based on the Gini impurity decrease.

1.16. Codes (Machine learning analysis)

```
import pandas as pd

import numpy as np

import matplotlib.pyplot as plt

import seaborn as sns

import networkx as nx

from sklearn.preprocessing import StandardScaler

from sklearn.manifold import TSNE

from sklearn.model_selection import StratifiedKFold

from sklearn.linear_model import LogisticRegression

from sklearn.ensemble import RandomForestClassifier

from sklearn.svm import SVC

from sklearn.metrics import roc_curve, auc
```

```
import warnings
# --- Global Configuration ---
warnings.filterwarnings('ignore')
sns.set_style("white")
sns.set_context("talk")
plt.rcParams['font.family'] = 'sans-serif'
plt.rcParams['savefig.dpi'] = 300
FILE_PATH = ' D:\plant\Plant data analysis.xlsx'
def load_and_normalize_data(file_path):
    """Loads data and performs I_target / I_tag normalization."""
    try:
        df_data = pd.read_excel(file_path, sheet_name='Data')
        df_tags = pd.read_excel(file_path, sheet_name='SERS nanotag')
    except Exception as e:
        print(f"Error loading file: {e}")
        return None
    # Normalization Map
    norm_map = pd.Series(df_tags.Intensity.values, index=df_tags['SERS
nanotag']).to_dict()
    biomarkers = ['PUB4', 'Clpt2', 'Vpg', 'Hc-pro', 'CP']
    # Apply Normalization
    df_norm = df_data.copy()
```

```
    for marker in biomarkers:
        if marker in norm_map:
            df_norm[marker] = df_data[marker] / norm_map[marker]
    # Rename Groups for publication quality labels
    df_norm['Group'] = df_norm['Group'].replace({
        'CK': 'Healthy Control',
        'PVY': 'PVY-Infected',
        'PVY+NNM': 'PVY+NNM'
    })
    return df_norm, biomarkers
# --- Figure 6b: Interaction Networks (Phased) ---
def plot_fig_6b_networks(df, biomarkers):
    """Generates Network Interaction graphs for PVY and PVY+NNM across 3
phases."""
    print("Generating Figure 6b: Interaction Networks...")
    # Define Phases
    phases = {
        'Day 1-3': [1, 3],
        'Day 5-7': [5, 7],
        'Day 9-11': [9, 11]
    }
    groups_to_plot = ['PVY-Infected', 'PVY+NNM']
```

```
fig, axes = plt.subplots(len(groups_to_plot), 3, figsize=(18, 12))
for row, group in enumerate(groups_to_plot):
    for col, (phase_name, days) in enumerate(phases.items()):
        ax = axes[row, col]
        # Filter Data
        subset = df[(df['Group'] == group) & (df['Day'].isin(days))]
        # Correlation Matrix
        corr_mat = subset[biomarkers].corr()
        # Build Graph
        G = nx.Graph()
        for bm in biomarkers: G.add_node(bm)
        # Add edges for correlation > 0.2 (Threshold mentioned in Exp.
Section)
        for i, b1 in enumerate(biomarkers):
            for j, b2 in enumerate(biomarkers):
                if i < j:
                    r = corr_mat.loc[b1, b2]
                    if abs(r) > 0.2:
                        G.add_edge(b1, b2, weight=abs(r))
        # Layout and Drawing
        pos = nx.circular_layout(G)
        weights = [G[u][v]['weight'] * 5 for u, v in G.edges()]
```

```
            nx.draw_networkx_nodes(G, pos, node_color='lightblue',
node_size=2000, ax=ax)
            nx.draw_networkx_labels(G, pos, font_size=10, font_weight='bold',
ax=ax)
            nx.draw_networkx_edges(G, pos, width=weights,
edge_color='#333333', ax=ax)
            ax.set_title(f"{group}\n{phase_name}")
            ax.axis('off')
    plt.tight_layout()
    plt.show()
# --- Figure 6c: t-SNE Trajectory (PVY vs PVY+NNM) ---
def plot_fig_6c_trajectory(df, biomarkers):
    """Generates t-SNE trajectory for Infected vs Treated groups."""
    print("Generating Figure 6c: t-SNE Trajectory...")
    subset = df[df['Group'].isin(['PVY-Infected', 'PVY+NNM'])].copy()
    # Z-score normalization for ML/t-SNE
    X = StandardScaler().fit_transform(subset[biomarkers]
    # t-SNE
    tsne = TSNE(n_components=2, random_state=42, perplexity=30)
    embedding = tsne.fit_transform(X)
    subset['tSNE1'] = embedding[:, 0]
    subset['tSNE2'] = embedding[:, 1]
```

```
    plt.figure(figsize=(8, 6))
    sns.scatterplot(
        data=subset, x='tSNE1', y='tSNE2',
        hue='Group', style='Day',
        palette=['#1f77b4', '#ff7f0e'], s=100, alpha=0.8
    )
    plt.title("Figure 6c: Temporal Trajectory (PVY vs PVY+NNM)")
    plt.legend(bbox_to_anchor=(1.05, 1), loc='upper left')
    plt.tight_layout()
    plt.show()
# --- Figure 6f: t-SNE Day 1 (Early Stratification) ---
def plot_fig_6f_day1_stratification(df, biomarkers):
    """Generates t-SNE for Day 1 showing 3 distinct clusters."""
    print("Generating Figure 6f: Day 1 Stratification...")
    subset = df[df['Day'] == 1].copy()
    X = StandardScaler().fit_transform(subset[biomarkers])
    # Lower perplexity for smaller sample size at Day 1
    tsne = TSNE(n_components=2, random_state=42, perplexity=10)
    embedding = tsne.fit_transform(X)
    subset['tSNE1'] = embedding[:, 0]
    subset['tSNE2'] = embedding[:, 1]
    plt.figure(figsize=(7, 6))
```

```
    sns.scatterplot(
        data=subset, x='tSNE1', y='tSNE2',
        hue='Group', palette="Set2", s=150, edgecolor='k'
    )
    plt.title("Figure 6f: Day 1 Stratification")
    plt.legend(loc='best')
    plt.tight_layout()
    plt.show(
# --- Figure 6g & 6h: ML Diagnosis (ROC & Feature Importance) ---
def run_ml_diagnosis_day1(df, biomarkers):
    """
    Generates ROC (Fig 6g) and Feature Importance (Fig 6h) for Day 1
    (Healthy vs PVY-Infected) using LR, RF, and SVM.
    """
    print("Generating Figure 6g & 6h: ML Diagnosis (Day 1)...")
    # Filter Day 1, Healthy vs PVY
    subset = df[(df['Day'] == 1) & (df['Group'].isin(['Healthy Control', 'PVY-
Infected']))].copy()
    subset['Target'] = subset['Group'].apply(lambda x: 1 if x == 'PVY-Infected' else 0)
    X = StandardScaler().fit_transform(subset[biomarkers])
    y = subset['Target'].values
    models = {
```

```
    'Logistic Regression': LogisticRegression(random_state=42),
    'Random Forest': RandomForestClassifier(random_state=42),
    'SVM': SVC(kernel='linear', probability=True, random_state=42)
}
cv = StratifiedKFold(n_splits=5, shuffle=True, random_state=42)
# Setup Plots
fig_roc, ax_roc = plt.subplots(figsize=(7, 6))
fig_imp, axes_imp = plt.subplots(1, 3, figsize=(18, 5))
colors = ['#1f77b4', '#ff7f0e', '#2ca02c']
for i, (name, model) in enumerate(models.items()):
    tprs = []
    aucs = []
    mean_fpr = np.linspace(0, 1, 100)
    importances = []
    # Cross Validation Loop
    for train, test in cv.split(X, y):
        model.fit(X[train], y[train])
        # ROC Logic
        probas_ = model.predict_proba(X[test])
        fpr, tpr, _ = roc_curve(y[test], probas_[:, 1])
        tprs.append(np.interp(mean_fpr, fpr, tpr))
        tprs[-1][0] = 0.0
```

```
        roc_auc = auc(fpr, tpr)
        aucs.append(roc_auc)
        # Feature Importance Logic
        if name == 'Logistic Regression' or name == 'SVM':
            importances.append(np.abs(model.coef_[0]))
        elif name == 'Random Forest':
            importances.append(model.feature_importances_)
    # Plot Mean ROC
    mean_tpr = np.mean(tprs, axis=0)
    mean_tpr[-1] = 1.0
    mean_auc = auc(mean_fpr, mean_tpr)
    ax_roc.plot(mean_fpr, mean_tpr, color=colors[i], lw=2,
                label=f'{name} (AUC = {mean_auc:.2f})')
    # Plot Feature Importance (Average over folds)
    mean_importance = np.mean(importances, axis=0)
    imp_df = pd.DataFrame({'Feature': biomarkers, 'Importance':
mean_importance})
    imp_df = imp_df.sort_values(by='Importance', ascending=True)
    axes_imp[i].barh(imp_df['Feature'], imp_df['Importance'], color=colors[i],
alpha=0.7)
    axes_imp[i].set_title(f"{name} Importance")
    axes_imp[i].set_xlabel("Relative Importance")
```

```
    # Finalize ROC Plot (Fig 6g)
    ax_roc.plot([0, 1], [0, 1], linestyle='--', lw=2, color='grey', alpha=.8)
    ax_roc.set_xlabel('False Positive Rate')
    ax_roc.set_ylabel('True Positive Rate')
    ax_roc.set_title('Figure 6g: ROC Curves (Day 1)')
    ax_roc.legend(loc="lower right")
    # Finalize Feature Importance Plot (Fig 6h)
    fig_imp.suptitle('Figure 6h: Feature Importance by Algorithm')
    plt.tight_layout()
    plt.show()
# --- Main Execution ---
if __name__ == "__main__":
    df_norm, biomarkers = load_and_normalize_data(FILE_PATH)
    if df_norm is not None:
        plot_fig_6b_networks(df_norm, biomarkers)
        plot_fig_6c_trajectory(df_norm, biomarkers)
        plot_fig_6f_day1_stratification(df_norm, biomarkers)
        run_ml_diagnosis_day1(df_norm, biomarkers)
        print("Analysis Complete.")
```

Supplementary Tables

Table S1: Differentially expressed genes identified by RNA sequencing.

up -regulation	down -regulation
Niben101Scf34538g00005	Niben101Scf03374g06002
Niben101Scf03328g02014	Niben101Scf01237g11010
Niben101Scf00294g01014	Niben101Scf02160g01003
Niben101Scf03460g04004	Niben101Scf04040g08010
Niben101Scf02427g03007	Niben101Scf06684g01005

Table S2. Involved oligonucleotides and the corresponding sequences.

Name	Sequence (5' - 3')

sgRNA1-Vpg	**AAAGGCAAAGGUACCACAGU**GUUUUAGAGCUAGAA AUAGCAAGUUAAAAUAAGGCUAGUCCGUUAUCAAC UUGAAAAAGUGGCACCGAGUCGGUGCUUUU
sgRNA2-Vpg	**UGACUUGCCCAUACCAACUG**GUUUUAGAGCUAGAA AUAGCAAGUUAAAAUAAGGCUAGUCCGUUAUCAAC UUGAAAAAGUGGCACCGAGUCGGUGCUUUU
sgRNA1-CP	**ACGAACACCAGTGAGGGCTA**GUUUUAGAGCUAGAA AUAGCAAGUUAAAAUAAGGCUAGUCCGUUAUCAAC UUGAAAAAGUGGCACCGAGUCGGUGCUUUU
sgRNA2-CP	**GGGCAGGUCAUCUUAUAGCA**GUUUUAGAGCUAGAA AUAGCAAGUUAAAAUAAGGCUAGUCCGUUAUCAAC UUGAAAAAGUGGCACCGAGUCGGUGCUUUU
sgRNA1-Hc-pro	**GGCUUACCAGUUGAAGACUG**GUUUUAGAGCUAGAA AUAGCAAGUUAAAAUAAGGCUAGUCCGUUAUCAAC UUGAAAAAGUGGCACCGAGUCGGUGCUUUU
sgRNA2-Hc-pro	**GGGCAGGUCAUCUUAUAGCA**GUUUUAGAGCUAGAA AUAGCAAGUUAAAAUAAGGCUAGUCCGUUAUCAAC UUGAAAAAGUGGCACCGAGUCGGUGCUUUU
sgRNA1-ClpT2	**GAACAGAAGCUCUUCUCAUG**GUUUUAGAGCUAGAA AUAGCAAGUUAAAAUAAGGCUAGUCCGUUAUCAAC UUGAAAAAGUGGCACCGAGUCGGUGCUUUU
sgRNA2-ClpT2	**UCAUGGGGAUUUUGAUUGAA**GUUUUAGAGCUAGAA AUAGCAAGUUAAAAUAAGGCUAGUCCGUUAUCAAC UUGAAAAAGUGGCACCGAGUCGGUGCUUUU
sgRNA1-PUB4	**AGACCAAAACAGCGAUGUGA**GUUUUAGAGCUAGAA AUAGCAAGUUAAAAUAAGGCUAGUCCGUUAUCAAC UUGAAAAAGUGGCACCGAGUCGGUGCUUUU
sgRNA2-PUB4	**UGCAACAUAUCUACUAAUAG**GUUUUAGAGCUAGAA AUAGCAAGUUAAAAUAAGGCUAGUCCGUUAUCAAC UUGAAAAAGUGGCACCGAGUCGGUGCUUUU
PCR Product-Vpg (GenBank:KC005988.1)	GGGCTGGCTTTGAAATTGACAACAATGATGACACAAT AGAGGAATTCTTTGGTTCTGCATACAGGAAAAAGGGA AAAGGCAAAGGTACCACAGTTGGTATGGGCAAGTCA AGCAGGAAGTTCATCA
PCR Product-CP (GenBank:MN563134.1)	AATCTGCGCGATGGAAGTTTGGCTCGCTATGCTTTTG ACTTTTATGAGGTCACATCACGAACACCAGTGAGGGC TAGGGAAGCGCACATTCAAATGAAGGCCGCAGCATT GAAATCAGCCCAACCTCGACTTTTCGGGTTGGACGGT GGCATCAGTACACAAGA
PCR Product-Hc-pro (GenBank:MG019342.1)	TACATGTGTGGCAGGCTTACCAGTTGAAGACTGCGGC AGAGTTGCAGCGATAATGACACACAGTATTTTACCGT GCTATAAGATGACCTGCCCTACCTGTGCCCAACAATA

	TGCTAACTTGCCAGCCAGTGACTTACTTAAGATATTA CACAAGCACGCAAGTGATGGTTTAAATCGATTGGGGG CAGACAAAGA
PCR Product-Clpt2 (GenBank:XM_019396954.1)	CCTAAATGGTCGTCAAAGGCGATAAAATCTTTTGCCA TGGGTGAATTGGAAGCAAGAAAACTTAAATATTCCAC TACTGGAACAGAAGCTCTTCTCATGGGGATTTTGATT GAAGGGACAAATTTTGCTTCAAAATATTTGAGGACGA ACAGCATTACTCTCT
PCR Product-PUB4 (GenBank:M_019384304.1)	CATTTGCCATCTGTTGTGCGGGATTCAGGTGCAATTCC GATTCTGGTTCAGCTGTTGGGAGACCAAAACAGCGAT GTGAGGGAGAAGGTTTCTGGAGCTATCGCCCAGTTGA GTTACAATGAAGCAGATAGAGCTGCTCTTGCAAATTC TGGAGCTATTCCGCTATTAGTAGATATGTTGCAGGAT GAGTCAGAGGAAATG
PCR primer-F -Vpg	**Biotin**-GGGCTGGCTTTGAAATTGACAAC
PCR primer-R-Vpg	TGATGAACTTCCTGCTTGACTTGC
PCR primer-F-CP	**Biotin**-AATCTGCGCGATGGAAGTTTGG
PCR primer-R-CP	TCTTGTGTACTGATGCCACCGT
PCR primer-F-Hc-pro	**Biotin**-TACATGTGTGGCAGGCTTACCA
PCR primer-R-Hc-pro	TCTTTGTCTGCCCCCAATCGAT
PCR primer-F-Clpt2	**Biotin**-CCTAAATGGTCGTCAAAGG
PCR primer-R-Clpt2	AGAGAGTAATGCTGTTCGTCC
PCR primer-F-PUB4	**Biotin**-CATTTGCCATCTGTTGTGC
PCR primer-R-PUB4	CATTTCCTCTGACTCATCCTG
RT-PCR primer-F-Vpg	GGGCTGGCTTTGAAATTGACAAC
RT-PCR primer-R-Vpg	TGATGAACTTCCTGCTTGACTTGC
RT-PCR primer-F-CP	AATCTGCGCGATGGAAGTTTGG
RT-PCR primer-R-CP	TCTTGTGTACTGATGCCACCGT
RT-PCR primer-F-Hc-pro	TACATGTGTGGCAGGCTTACCA
RT-PCR primer-R-Hc-pro	TCTTTGTCTGCCCCCAATCGAT
RT-PCR primer-F-Clpt2	CCTAAATGGTCGTCAAAGG
RT-PCR primer-R-Clpt2	AGAGAGTAATGCTGTTCGTCC
RT-PCR primer-F-PUB4	CATTTGCCATCTGTTGTGC
RT-PCR primer-R-PUB4	CATTTCCTCTGACTCATCCTG

Table S3. Comparison among different dCas9 sensors.

Strategy[a]	Method[b]	Target	Range	LOD	Ref.

dCas9-ELISA	Abs	CaMV35S	12.5 to 500 copies μL−1	12.5 copies μL^{-1}	Talanta 257 (2023) 124318
dCas9 powered impedimetric biosensor	EC	PIK3CA exon 9 mutation	2 nM-20 nM	0.65 nM	Anal Chim Acta 2020, 1121, 35-41
E-dCas9+PCR	EC	EGFR L858R, 19del, T790M	10 fM-1 nM; 10 fg/μL-1 ng/μL	2.86 fM; 6.35 fg/μL	Anal.Chem.2023,95,16305−16314
dCas9	SERS	HPV 16 DNA and HPV 18 DNA	30−190 ng	--	Anal. Chem. 2023, 95, 5927−5936
dCas9	SERS	G12C DNA G12V DNA	3.60 nM -18 nM	--	ACS Appl. Nano Mater. 2024, 7, 9800−9808
dCas9 +RPA	SERS	35S Promoter and Nos Terminator	--	--	Sensors and Actuators: B. Chemical 429 (2025) 137293
dCas9+EHD	SERS	Hc-Pro, Vpg, CP, PUB4, Clpt2	10 pM - 10000 pM	2.5 pM, 0.43 pM, 6.4 pM, 2.0 pM 3.6 pM	This work

a. ELISA: Enzyme-Linked Immunosorbent Assay; PCR: Polymerase Chain Reaction; RPA: recombinase polymerase amplification; EHD: Electrohydrodynamics

b. EC: electrochemical detection; SERS: surface-enhanced Raman scattering. Abs: Absorance

Supplementary Figures

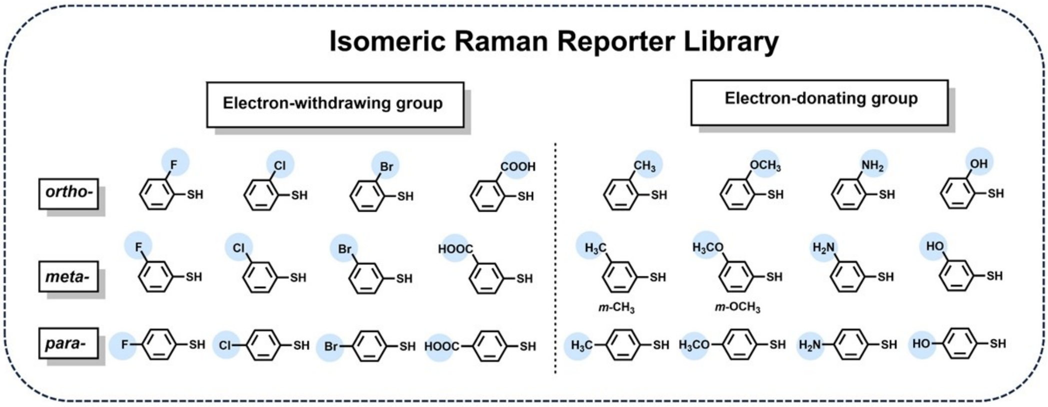

Figure S1. The molecular structure of all isomeric Raman Reporters.

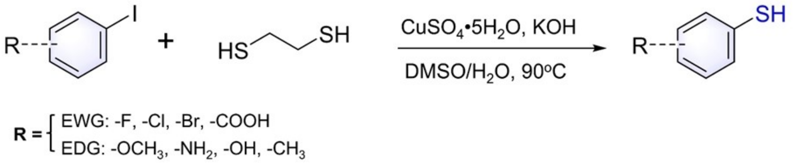

Figure S2. The synthetic route of Raman molecule.

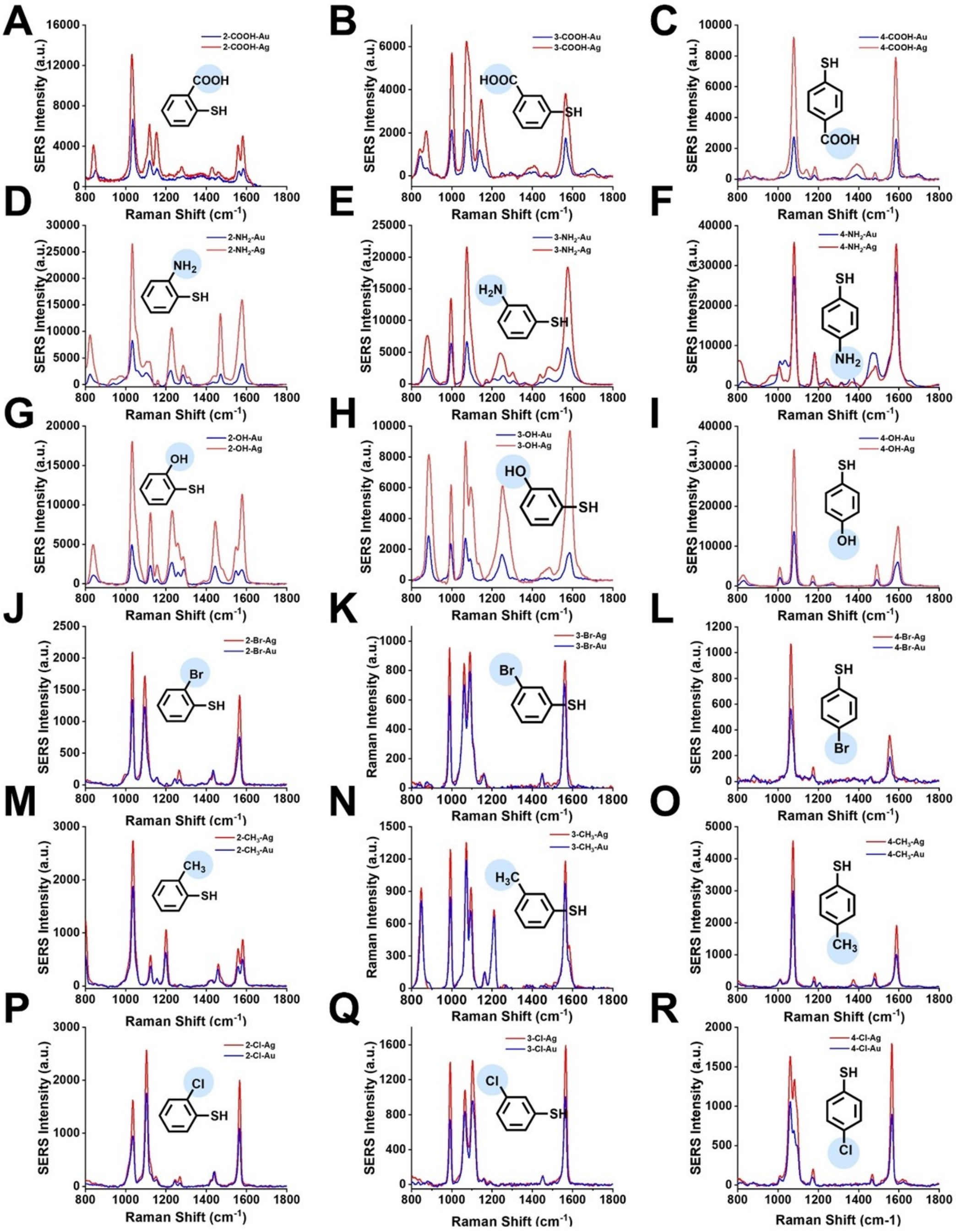

Figure S3. SERS spectra of various thiophenol derivatives**.** The spectra display thiophenols with different functional groups and substitution positions: (A-C) mercaptobenzoic acids (-COOH); (D-F) aminothiophenols (-NH_2); (G-I) hydroxythiophenols (-OH); (J-L) bromothiophenols (-Br); (M-O) methylthiophenols (-CH_3); and (P-R) chlorothiophenols (-Cl). Each row represents the *ortho*- (2-), *meta*- (3-), and *para*- (4-) isomers, respectively. Insets show the corresponding molecular structures.

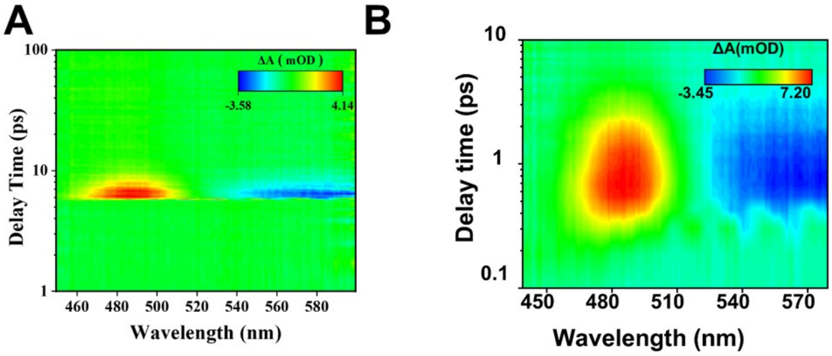

Figure S4. Two-dimensional (2D) transient absorption (TA) spectra. (A) TA spectrum of Cu_2O/CuO@Au@Ag nanoparticles. (B) TA spectrum of Cu_2O/CuO@Au@*p*-FTP@Ag nanoparticles.

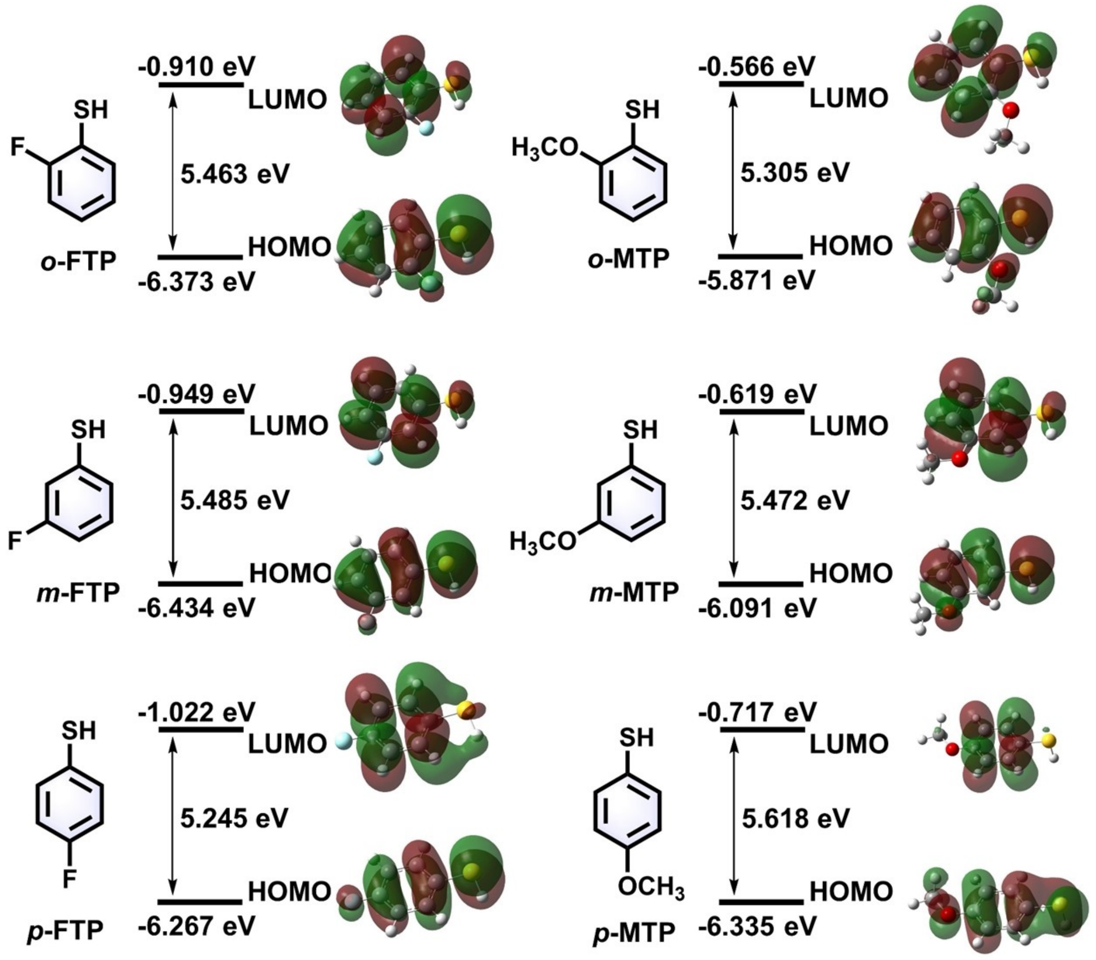

Figure S5. The Highest Occupied Molecular Orbital (HOMO) and Lowest Unoccupied Molecular Orbital (LUMO) for (A) *o*-FTP, (B) *o*-MTP, (C) *m*-FTP, (D) *m*-MTP, (E) *p*-FTP, and (F) *p*-MTP. For each compound, the orbital energies (in eV) for HOMO and LUMO are indicated, along with the corresponding HOMO-LUMO energy gap. The 3D representations of the HOMO (bottom) and LUMO (top) illustrate their spatial distribution and nodal patterns, calculated at the DFT B3LYP/6-311+G(d,p) level of theory.

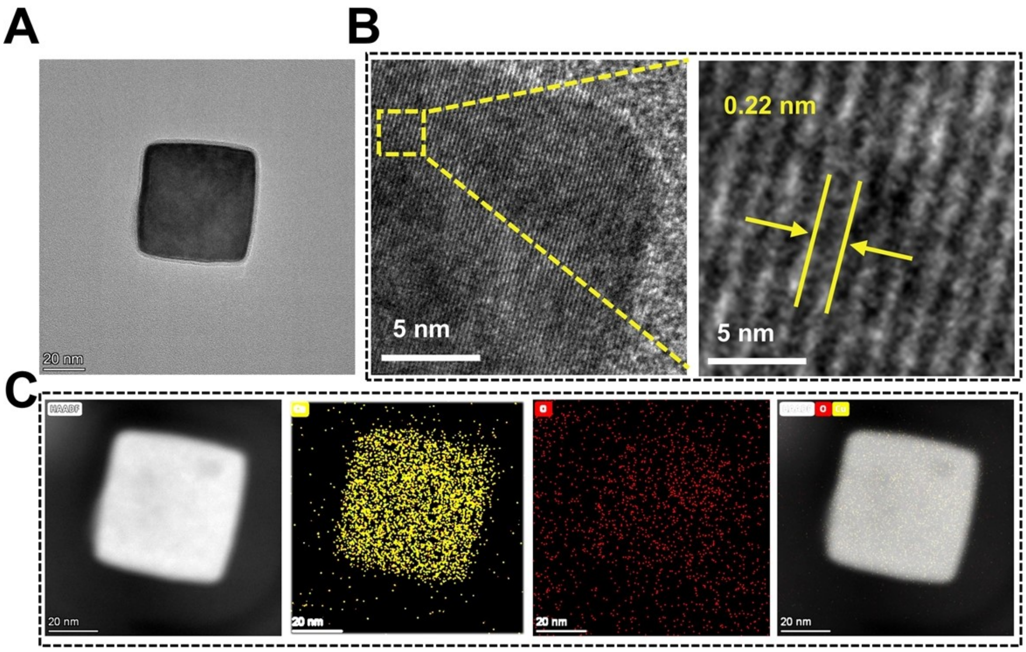

Figure S6. Morphological and elemental characterization of Cu_2O/CuO nanocubes. (A) Transmission electron microscopy (TEM) image showing the cubic morphology of the synthesized Cu_2O nanocrystals. Scale bar: 20 nm. (B) High-resolution transmission electron microscopy (HRTEM) image of Cu_2O/CuO nanocube. Scale bars: 5 nm. (C) Energy-dispersive X-ray (EDX) elemental mapping images of a single Cu_2O/CuO nanocube. Scale bars: 20 nm.

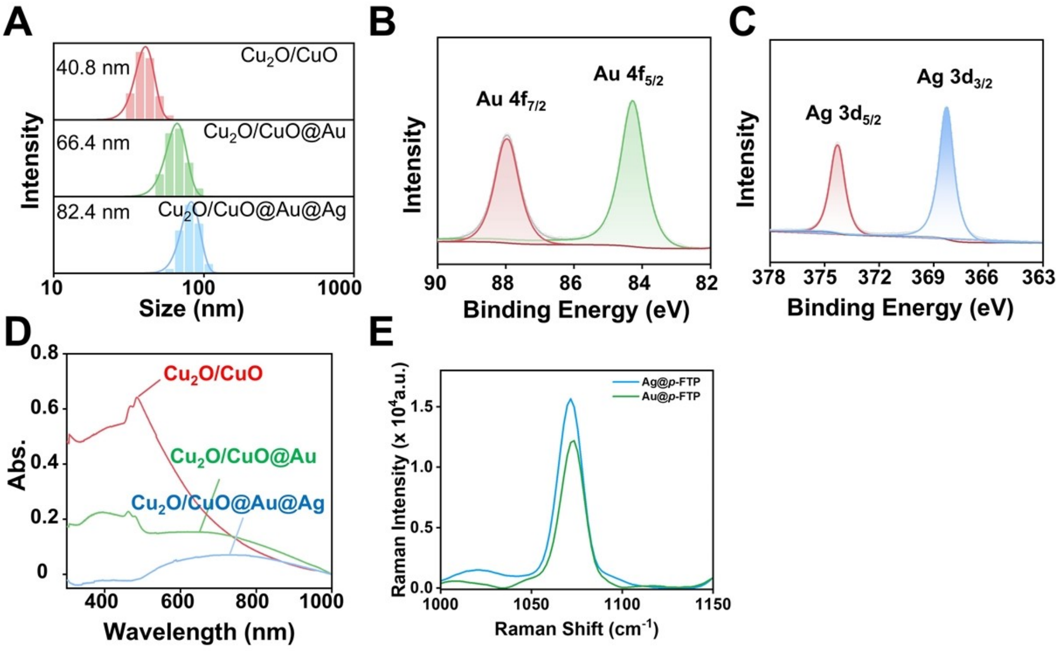

Figure S7. Surface and elemental characterization of Cu_2O/CuO@Au@Ag**.** (A) DLS size distribution profiles of Cu_2O/CuO, Cu_2O/CuO@Au and Cu_2O/CuO@Au@Ag. High-resolution X-ray photoelectron spectroscopy (XPS) spectrum of (B) Au 4f and (C) Ag 3d for Cu_2O/CuO@Au@Ag nanoparticles. (D) UV-Vis absorption spectra of Cu_2O/CuO (red line), Cu_2O/CuO@Au (green line), and Cu_2O/CuO@Au@Ag (blue line) samples. (E) Comparison of SERS signal enhancement by Ag and Au.

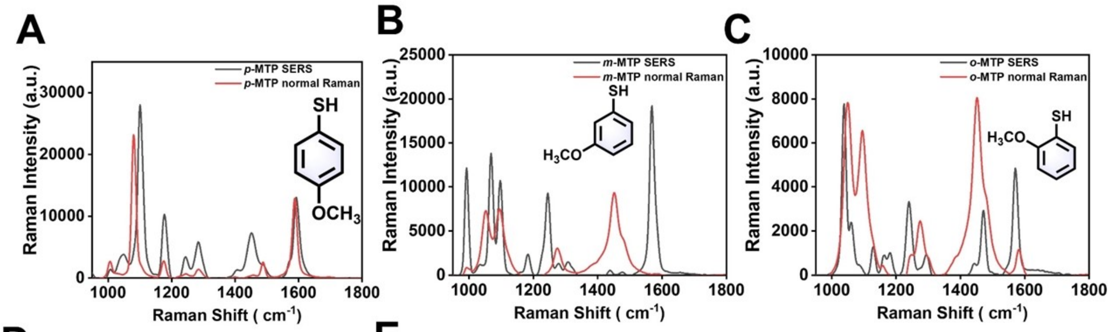

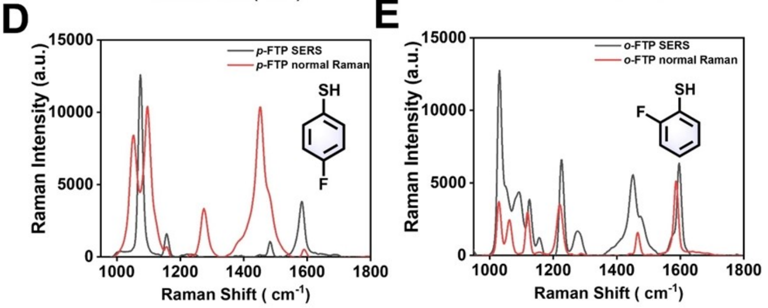

Reporter	EF
***p*-MTP**	**7.9X10^6**
***m*-MTP**	**0.3X10^6**
***o*-MTP**	**1.7X10^6**
***p*-FTP**	**6.3X10^6**
***o*-FTP**	**1.3X10^6**

Figure S8. Calculation of SERS Enhancement Factors (EFs) for five Raman reporters. The EFs based on the following formula: $EF=(I_{SERS}/N_{SERS})/(I_{RS}/N_{RS})$. The corresponding molecules are: (A) *p*-MTP; (B) *m*-MTP; (C) *o*-MTP; (D) *p*-FTP; (E) *o*-FTP.

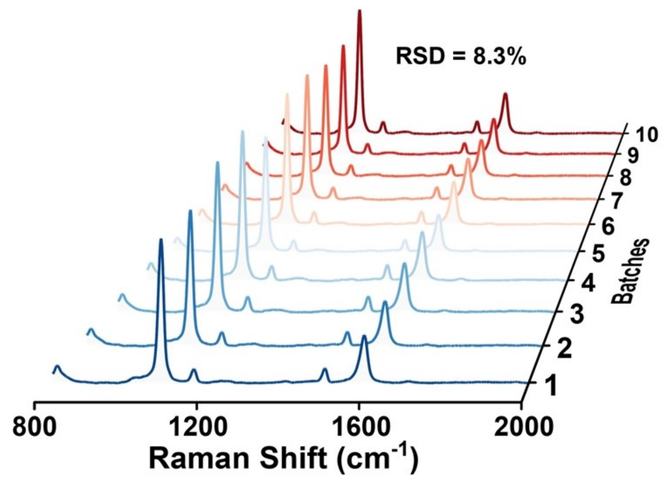

Figure S9. Evaluation of signal reproducibility of Cu_2O/CuO@Au@Ag. Raman spectrum of *p*-FTP collected on 10 batches of independently prepared Cu_2O/CuO@Au@Ag.

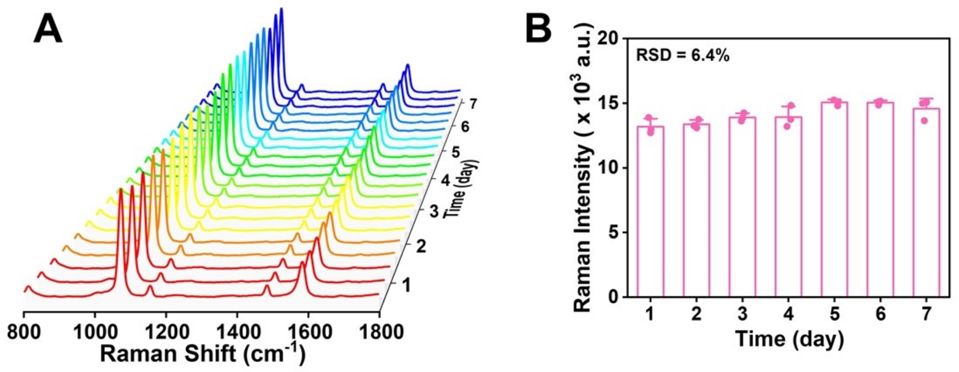

Figure S10: Evaluation of the longitudinal stability of the Cu_2O/CuO@Au@Ag. (A) Raman spectra of *p*-FTP enhanced by Cu_2O/CuO@Au@Ag on different days. (B) Tracking the signals of the SERS signal for *p*-FTP at 1073 cm^{-1} over 7 days (RSD = 6.4%). Data are presented as mean values ± standard deviation from three independent experiments.

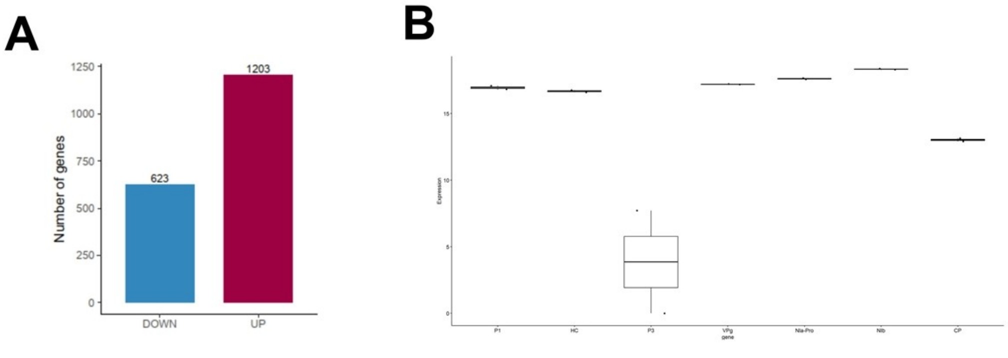

Figure S11. NNM causes distinct gene expression in Nicotiana benthamiana with PVY infection. (A) Number of genes with differential expressions that are up- (red) or down- (blue) regulated. (B) Transcriptomic profiling of the viral genome revealed distinct expression tiers.

```
Clpt2 sgRNA  -------------------------------------------UCAUGGGGAUUUUGAUUGAA-------
Clpt2        AATATTCCACTACTGGAACA-------------GAAGCTCTTCTCATGGGGATTTTGATTGAAGGGACAA
CP           -------------------------------------------TGCTGCGGCCTTCATTTGAAT------
HC-Pro       ACTTGCCAGCCAGTGACTTACTTAAGATATTACACAAGCACGCAAGTGATGGTTTAAATCGATTGGGGGC
PUB4         GTCTCCCAACAGCTGAACCA-----------------------GAATCGGAATTGCACCTGAATCCCGCA
Vpg          -------------------------------------------TGATGAACTTCCTGCTTGACTTGCCCA
```

Figure S12. Nucleotide sequence alignment of Clpt2 sgRNA with its target and non-target DNA strands. Red highlighting indicates nucleotides that are mismatched relative to the sgRNA sequence. The alignment demonstrates the high specificity of the sgRNA for the Clpt2 target.

```
CP sgRNA  ---------------ACGAACACCAGUGAGGGCUA-----------------------------------
CP        TTATGAGGTCACATCACGAACACCAGTGAGGGCTAGGGAAGCGCACATTC--------------------
Clpt2     ---AAATATTTGAGGACGAACAGCATTACTCTCT------------------------------------
HC-Pro    CTTAAGATATTACACAAGCACGCAAGTGATGGTTTAAATCGATTGGGGGCAGAC----------------
PUB4      ---CTGTTGGGAGACCAAAACAGCGATGTGAGGGAGAAGGTTTCTGGAGCTATCGCCCAGTTGAGTTACA
Vpg       --------CTTTGAAATTGACAACAATGATGACACAATAGAGGAATTCTTTGGTTCTGCATACAGGAAAA
```

Figure S13. Nucleotide sequence alignment of CP sgRNA with its target and non-target DNA strands. Red highlighting indicates nucleotides that are mismatched relative to the sgRNA sequence. The alignment demonstrates the high specificity of the sgRNA for the CP target.

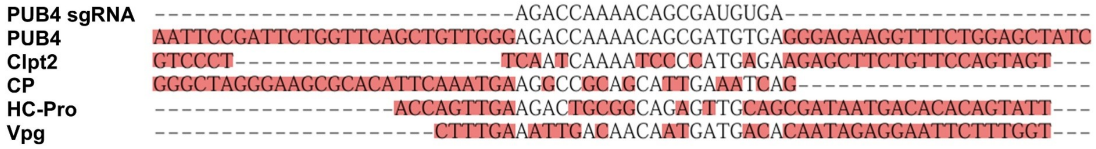

Figure S14. Nucleotide sequence alignment of PUB4 sgRNA with its target and non-target DNA strands. Red highlighting indicates nucleotides that are mismatched relative to the sgRNA sequence. The alignment demonstrates the high specificity of the sgRNA for the PUB4 target.

```
Vpg sgRNA  ----------------------------------UGACUUGCCCAUACCAACUG----------------
Vpg        ------------------TGATGAACTTCCTGCTTGACTTGCCCATACCAACTGTGGTA-----------
Clpt2      TTCCAGTAGTGGAATATTTAAGTTTTCTTGCTTCCAATTCACCCATGGCAAAAGATTTT-----------
CP         -------------------------------GTTTGGCTCGCTATGCTTTTGACTTTTATGAGGTCACAT
HC-Pro     -----GACACACAGTATTTTACCGTGCTATAAGATGACCTGCCCTACCTGTGCCCAACAATATGCTAACT
PUB4       CTCCCAACAGCTGAACCAGAATCGGAATTGCACCTGAATCCCGCACAACAGATGGCAAA-----------
```

Figure S15. Nucleotide sequence alignment of Vpg sgRNA with its target and non-target DNA strands. Red highlighting indicates nucleotides that are mismatched relative to the sgRNA sequence. The alignment demonstrates the high specificity of the sgRNA for the Vpg target.

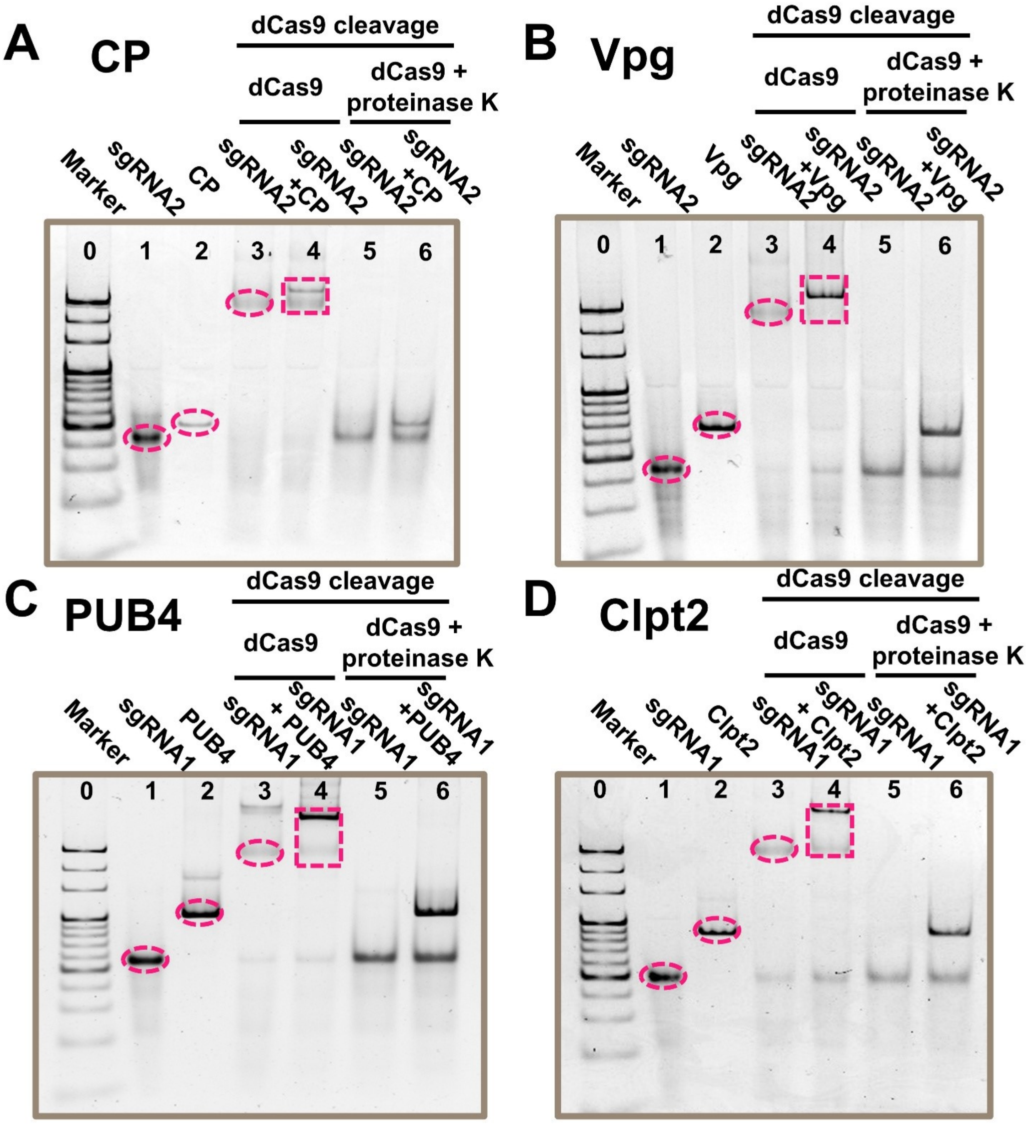

Figure S16. PAGE validation of dCas9-sgRNA (dRNP)-DNA complex. (A-D) PAGE showing the formation of dCas9-sgRNA-DNA complexes. Each panel represents a different target DNA fragment: (A) CP, (B) Vpg, (C) PUB4, and (D) Clpt2. For each panel: Lane 0: Marker. Lane 1 (sgRNA alone) shows free sgRNA (pink dashed oval). Lane 2: Target DNA fragment. Lane 3: sgRNA + dCas9. Lane 4 (sgRNA + dCas9 + DNA) demonstrates the shifted dRNP-DNA complex (pink dashed rectangle), indicating successful complex formation. Lanes 5 (sgRNA + DNA + proteinase K) and 6 (sgRNA + dCas9 + DNA + proteinase K) serve as controls, with proteinase K treatment confirming the protein-mediated nature of the shifted complex.

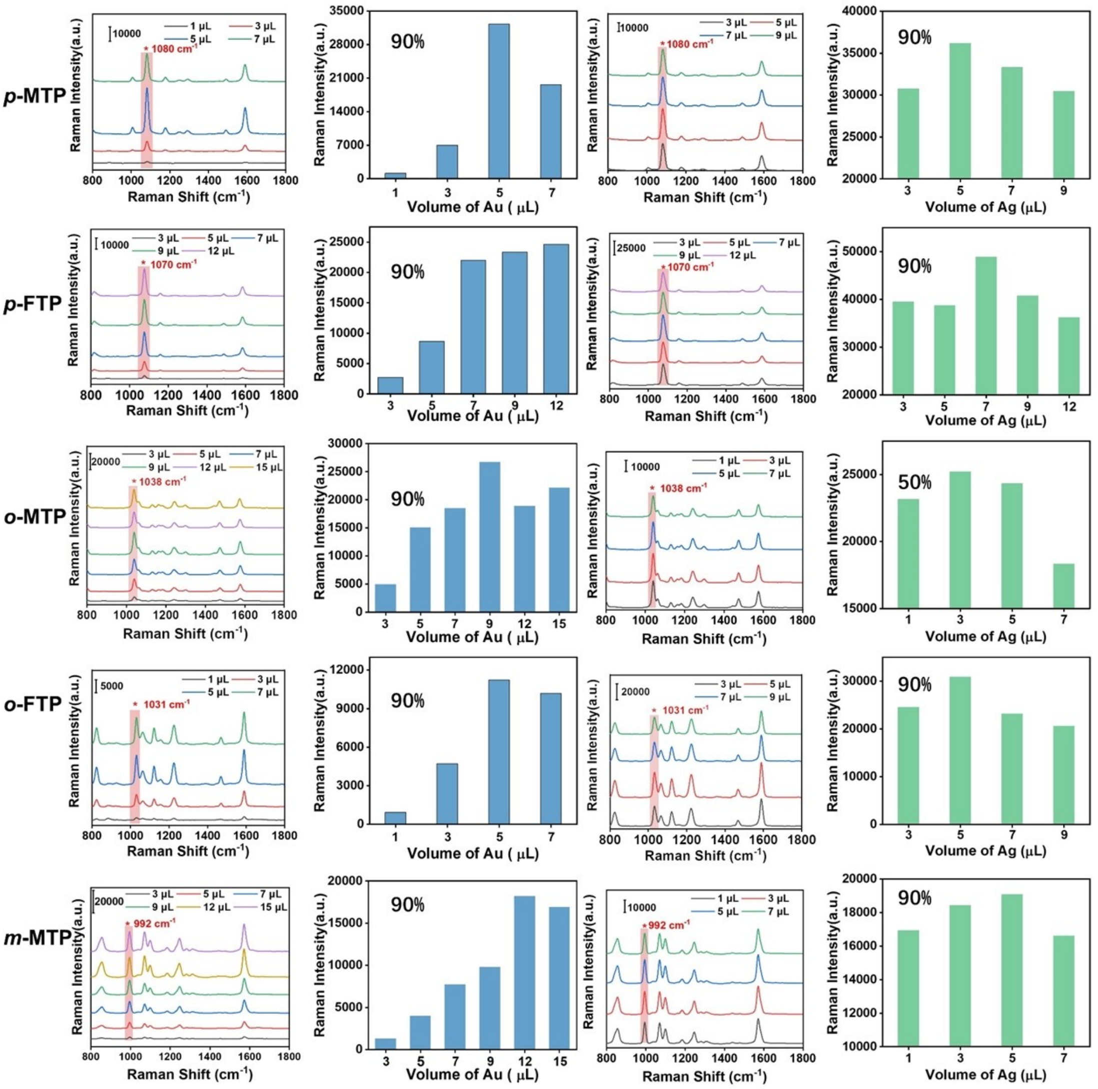

Figure S17: Optimization of Au and Ag for p-MTP, p-MTP, o-MTP, o-FTP and m-MTP. (A, C, E, G, I, K, M, O, Q, S) Raman spectra demonstrating the effect of varying gold (Au) and silver (Ag) nanoparticle dispersion volumes on the SERS signal intensity for five different thiophenol derivatives: *p*-MTP, *p*-MTP, *o*-MTP, *o*-FTP and *m*-MTP. Each row corresponds to a specific thiophenol derivative, with the left panels (A, E, I, M, Q) showing spectra with increasing Au nanoparticle volumes and the right panels (C, G, K, O, S) showing spectra with increasing Ag nanoparticle volumes. Highlighted peaks indicate the characteristic Raman shifts monitored for each compound (e.g., ~1080 cm^{-1} for *p*-MTP, ~1070 cm^{-1} for *p*-FTP, ~1038 cm^{-1} for *m*-MTP, ~1031 cm^{-1} for *o*-FTP, ~862 cm^{-1} for *m*-MTP).

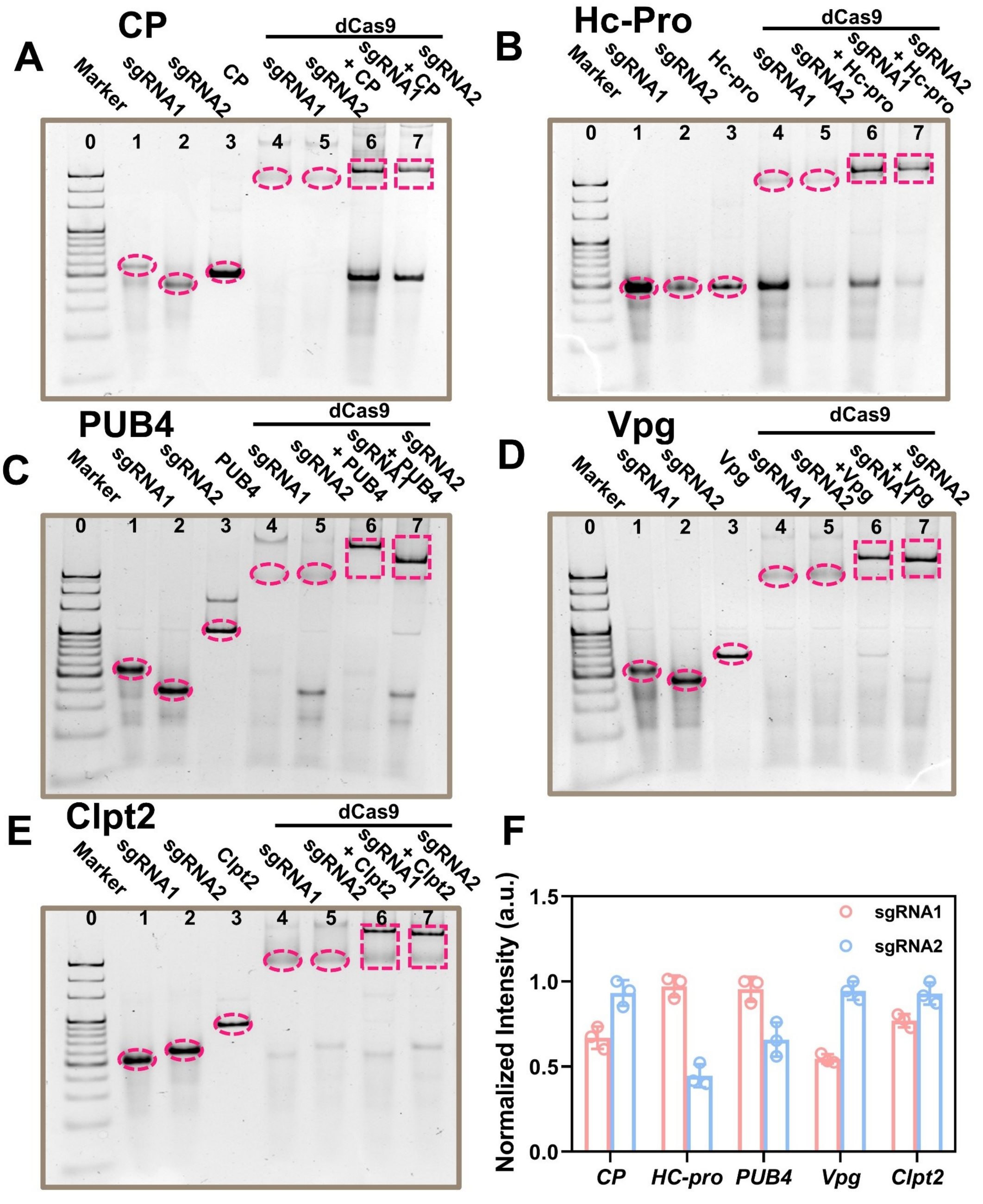

Figure S18. Optimization of dCas9-sgRNA (dRNP)-DNA complex formation specificity via PAGE and SERS signal analysis**.** (A-E) (A-E) PAGE demonstrating the specific formation of dCas9-sgRNA-DNA complexes. These panels illustrate the optimization of sgRNA selection for various target DNA fragments. (A) CP (B) Hc-Pro (C) PUB4 (D) Vpg (E) Clpt2. For each panel (A-E): Lane 0: Marker. Lanes 1 and 2 show free sgRNA1 and sgRNA2, respectively (pink dashed ovals). Lane 3: Target DNA fragment. Lanes 4 and 5 represent dCas9 pre-incubated with sgRNA1 or sgRNA2, respectively. Lanes 6 and 7 show the binding reaction of dCas9 with the target DNA in the presence of either sgRNA1 (Lane 6) or sgRNA2 (Lane 7). (F) Optimization of sgRNA selection based on SERS signal intensity, reflecting the binding efficiency of sgRNA1 (red bars) and sgRNA2 (blue bars) to their respective dCas9-DNA targets for CP, Hc-Pro, PUB4, Vpg, and Clpt2. Data are presented as mean ± SD (n=3 independent experiments).

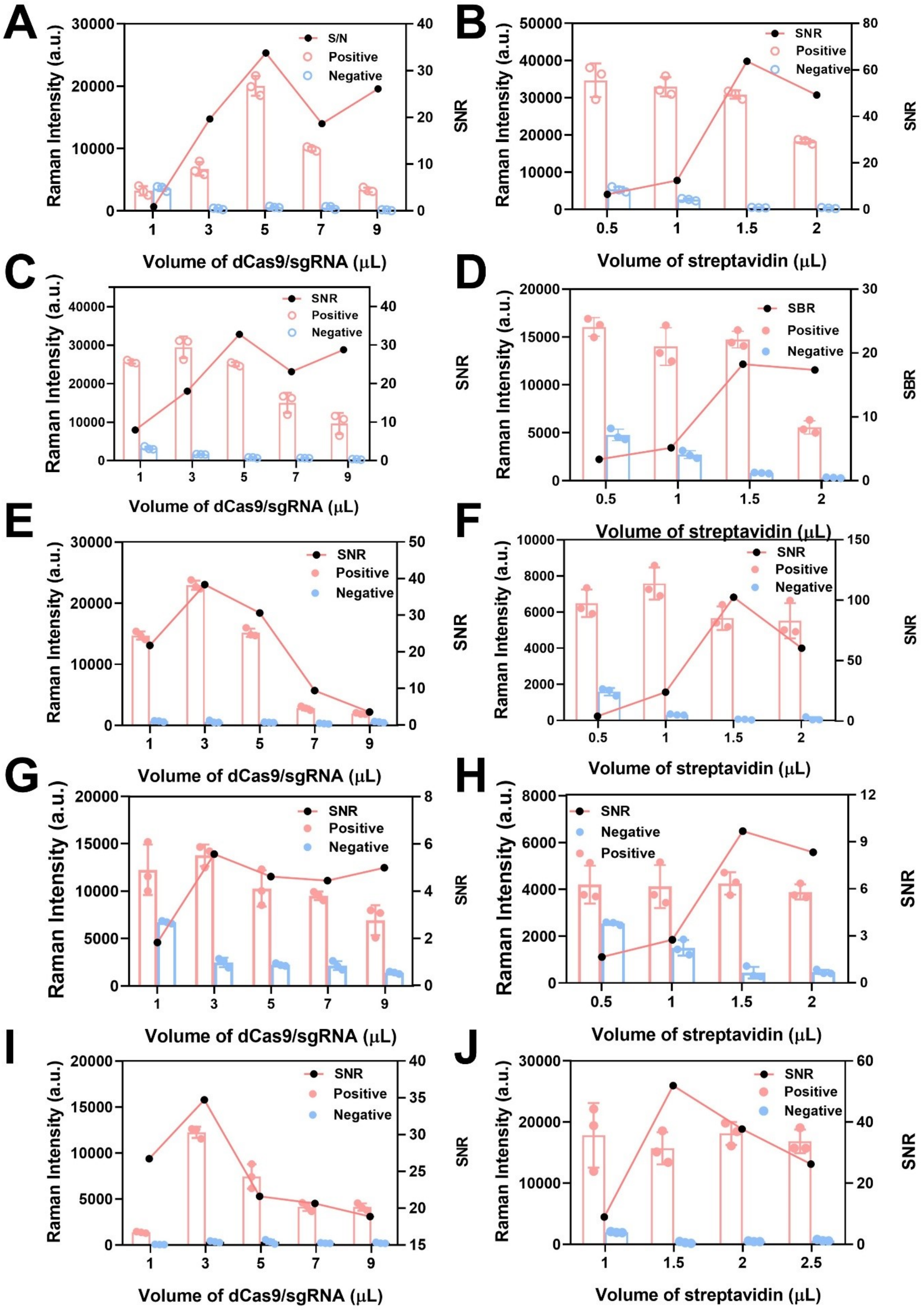

Figure S19. SERS signal optimization for dCas9/sgRNA binary complex and streptavidin volumes. Optimization of the dCas9/sgRNA binary complex volume (μL) to achieve maximal SERS signal intensity for five target genes: (A) CP, (C) Vpg, (E) PUB4, (G) Clpt2, and (I) Clpt2. Each graph displays the Raman intensity for positive (target present) and negative (target absent) samples (bar graphs, left y-axis, mean ± SD, n=3), along with the calculated Signal-to-Noise Ratio (SNR) (line graph, right y-axis). Optimization of streptavidin volume (μL) for SERS detection, corresponding to the same five target genes: (B) CP, (D) Vpg, (F) PUB4, (H) Clpt2, and (J) Clpt2. Each graph presents the Raman intensity for positive and negative samples (bar graphs, left y-axis, mean ± SD, n=3), and the calculated Signal-to-Background Ratio (SBR) (line graph, right y-axis).

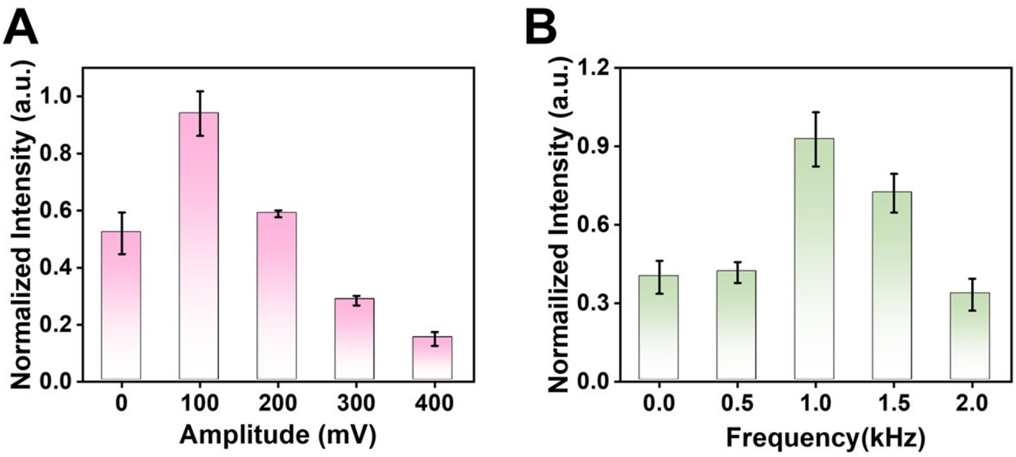

Figure S20. Optimization of EHD parameters for the measurement of the Hc-pro gene. (A) Effect of various amplitudes (0–400 mV) and (B) frequencies (0–2.0 kHz) on the normalized intensity. The error bars represent the standard deviation from three independent experiments.

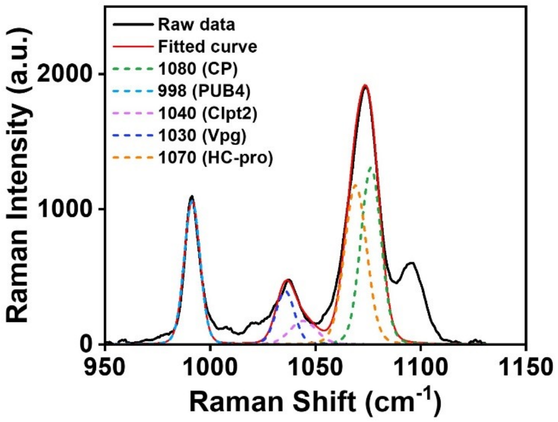

Figure S21. Demonstration of multiplexed detection of DNA in simulated buffer samples.

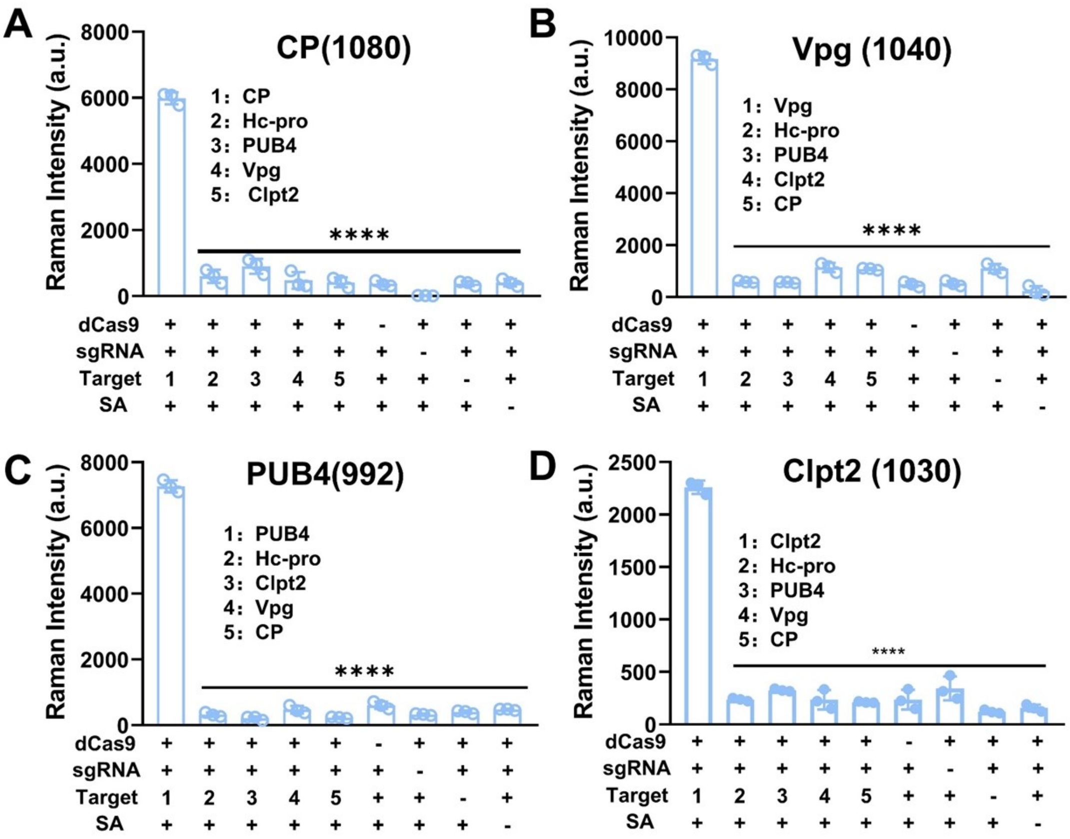

Figure S22. Specificity validation of the SERS-based detection system for viral and host genes. Raman signal intensity at the characteristic peak for (A) CP (1080 cm^{-1}), (B) Vpg (1040 cm^{-1}), (C) PUB4 (992 cm^{-1}), and (D) Clpt2 (1030 cm^{-1}) in the presence of various targets. The bars represent the Raman intensity when dCas9, sgRNA, and SA are present, with different targets (1-5) tested. Error bars represent the standard deviations (SD) of three independent experiments (n = 3). ****P < 0.0001.

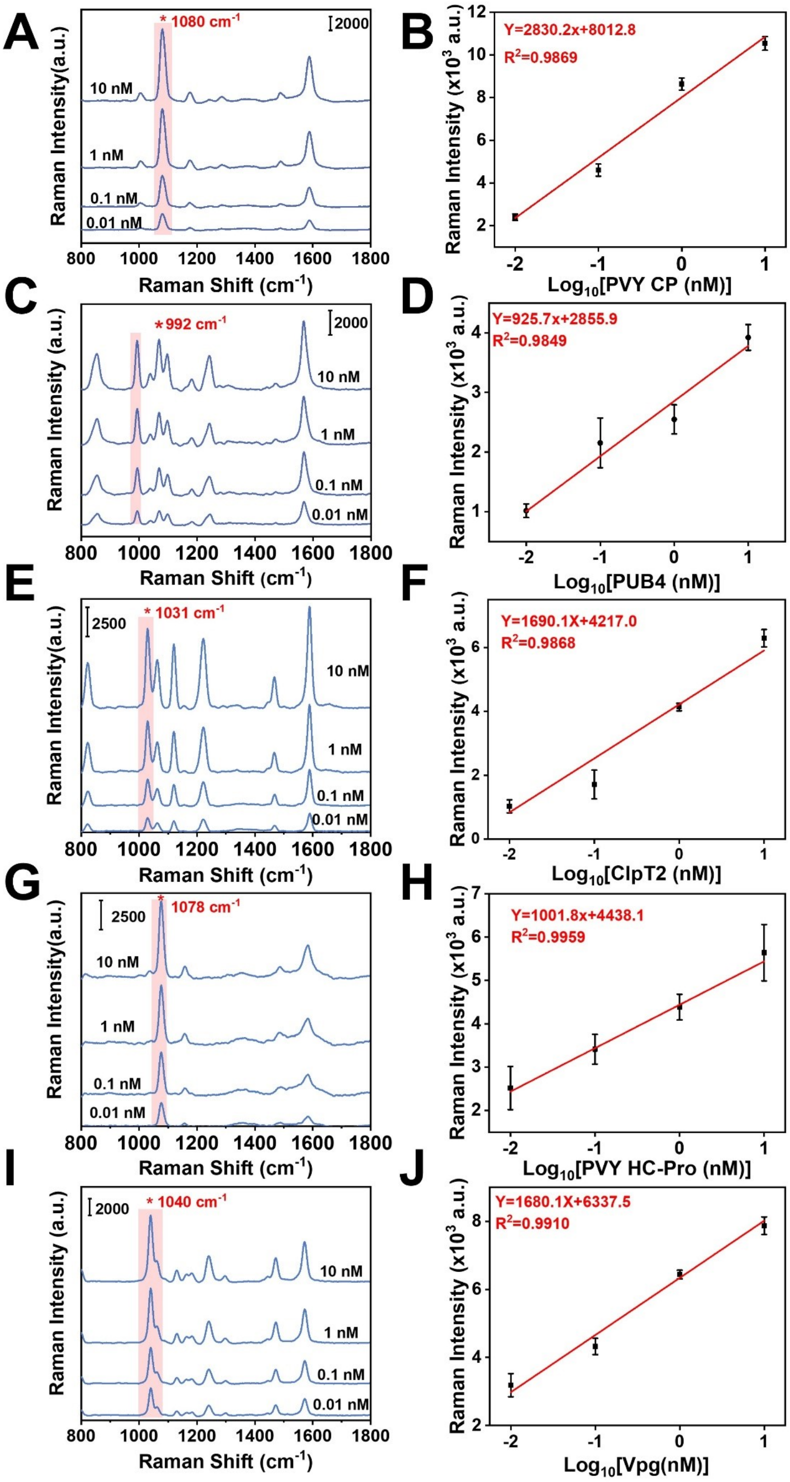

Figure S23. SERS-based detection of different viral and host genes at varying concentrations. Representative SERS spectra showing changes in signal intensity with increasing concentrations (0.01 nM, 0.1 nM, 1 nM, 10 nM) of (A) CP, (C) PUB4, (E) ClpT2, (G) HC-pro, and (I) Vpg. The characteristic Raman shift for each target is highlighted in red. Corresponding linear calibration curves demonstrating the relationship between the logarithm of target concentration and the characteristic SERS peak intensity for (B) CP, (D) PUB4, (F) Clpt2, (H) HC-pro, and (J) Vpg. Data points represent the mean ± standard deviation from three independent measurements.

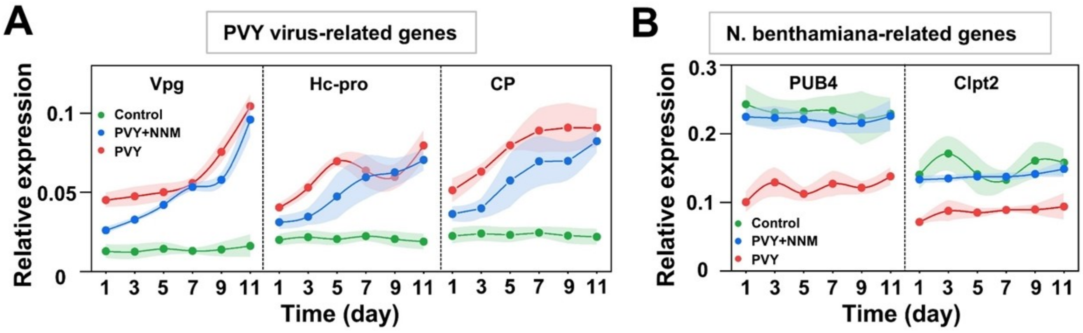

Figure S24: SERS-Based Monitoring of Gene Expression in N. benthamiana Under PVY Infection and Nanomaterial Treatment. (A) Relative Expression of PVY Virus-Related Genes: This panel presents the time-course expression profiles for three key genes associated with control, PVY and PVY+NNM: Vpg, Hc-pro and CP. (B) Relative Expression of N. benthamiana-Related Host Genes: This panel displays the time-course expression patterns of two endogenous N. benthamiana host genes: PUB4 and Clpt2. The shaded regions around each curve represent the standard error of the mean.

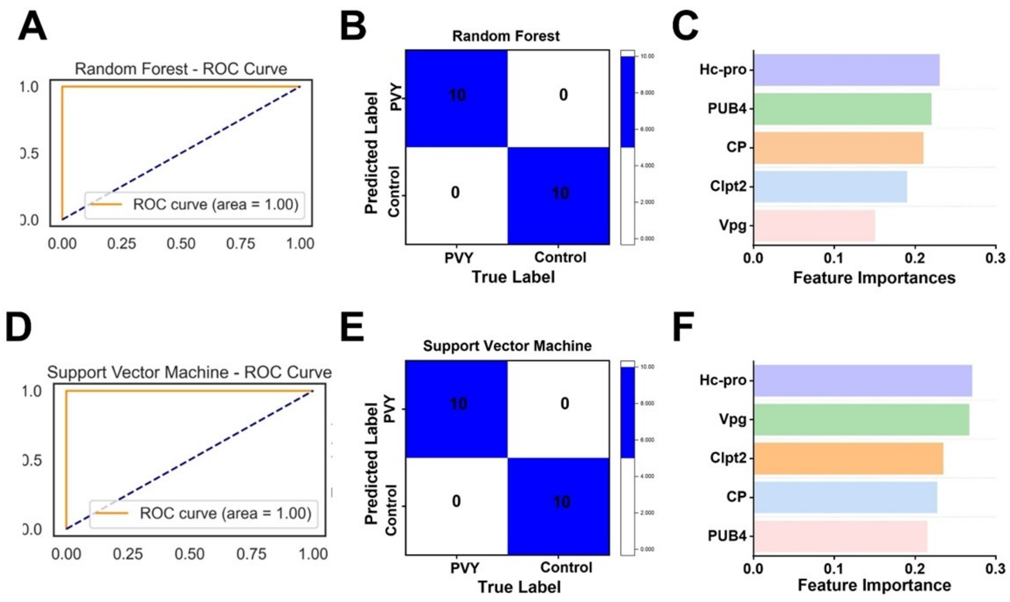

Figure S25. This figure presents the results of machine learning classification models**,** specifically Random Forest and Support Vector Machine, trained to distinguish between PVY-infected and control N. benthamiana samples based on gene expression data. (A) Receiver Operating Characteristic (ROC) curve for the Random Forest classifier. The orange line represents the ROC curve with an Area Under the Curve (AUC) of 1.00, indicating perfect discrimination between PVY and control samples. The dashed blue line represents a random classifier.(B) Confusion matrix for the Random Forest classifier. This matrix shows that the model perfectly classified 10 PVY samples as PVY and 10 control samples as control, with zero misclassifications (false positives or false negatives). (C) Bar graph illustrating the feature importances of the individual genes (Hc-pro, PUB4, CP, Clpt2, Vpg) as determined by the Random Forest model. Hc-pro and Vpg, both PVY-related genes, show the highest importance in distinguishing infection status. (D) Receiver Operating Characteristic (ROC) curve for the Support Vector Machine (SVM) classifier. Similar to the Random Forest model, the SVM also achieved an AUC of 1.00, demonstrating excellent discriminatory power. (E) Confusion matrix for the Support Vector Machine classifier. This matrix also shows perfect classification, correctly identifying 10 PVY samples and 10 control samples, with no misclassifications. (F) Bar graph depicting the feature importances of the individual genes as determined by the Support Vector Machine model. Consistent with the Random Forest results, Hc-pro and Vpg are identified as the most important features for classification.

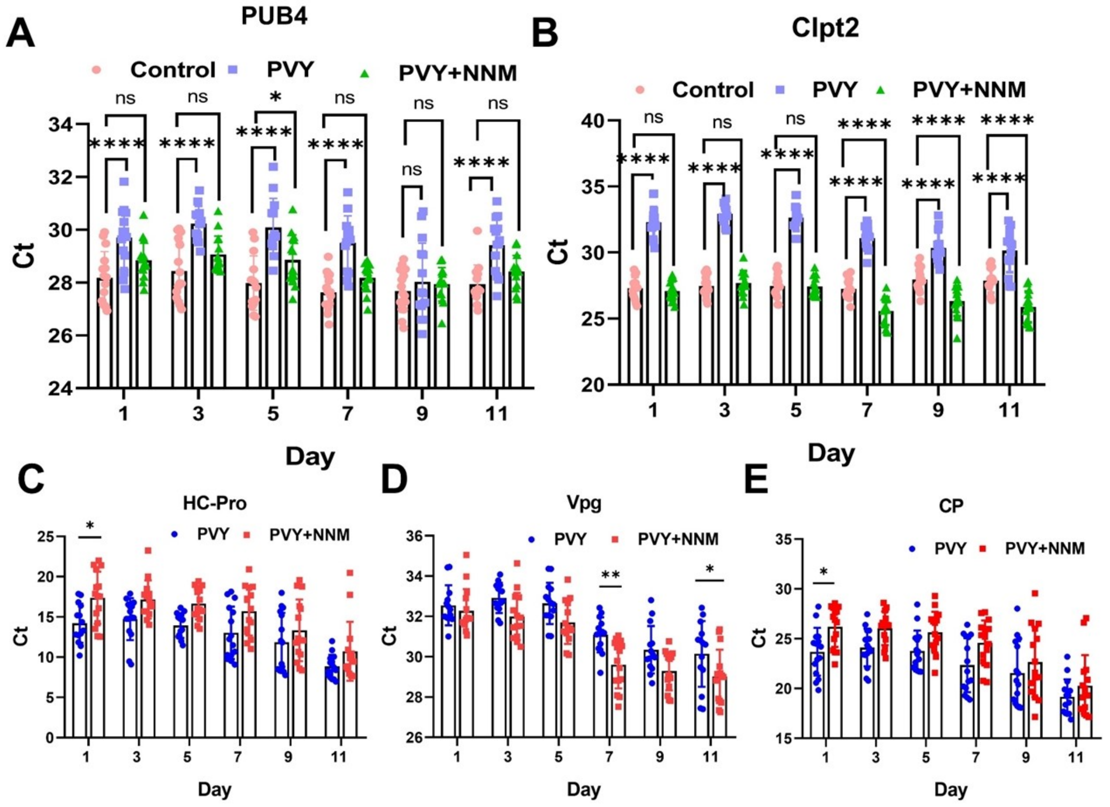

Figure S26. Relative expression levels of various genes in plants under different treatments and time points, determined by RT-PCR. Expression profiles of (A) PUB4, (B) Clpt2, (C) HC-Pro, (D) Vpg and (E) CP genes. For all panels, the y-axis represents the Ct value, and the x-axis represents the sampling day after treatment. Data are presented as individual data points overlaid on box plots (or bar graphs, if specified). Statistical significance was determined by [specify statistical test, e.g., one-way ANOVA with post-hoc Tukey's test] and is indicated as: ns (not significant), * ($p < 0.05$), ** ($p < 0.01$), *** ($p < 0.001$), **** ($p < 0.0001$). Treatments: CK (control), PVY (Potato Virus Y infection), PVY+NNM (PVY infection with NNM treatment).

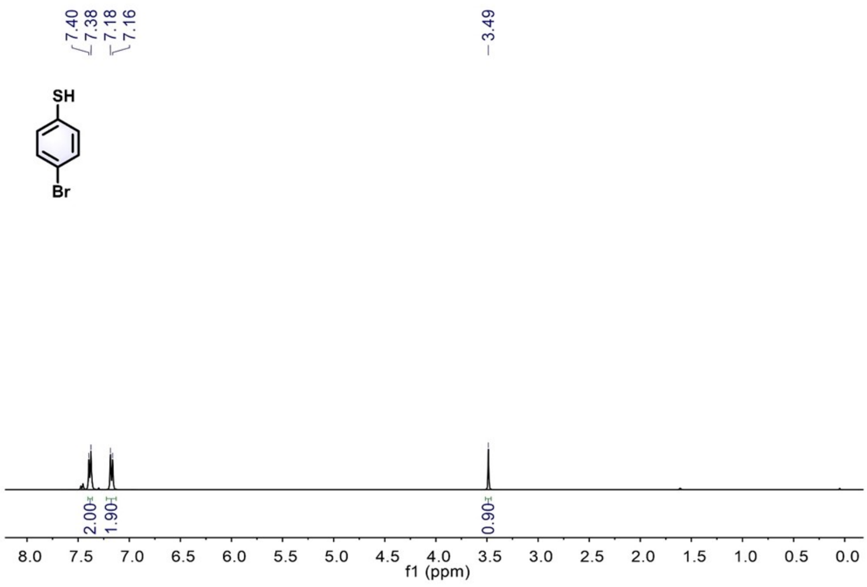

Figure S27. The ^{1}H NMR spectra of ***p*-Br** in $CDCl_3$. ^{1}H NMR (400 MHz, $CDCl_3$) δ 7.39 (d, J = 8.0 Hz, 2H), 7.17 (d, J = 8.1 Hz, 2H), 3.49 (s, 1H).

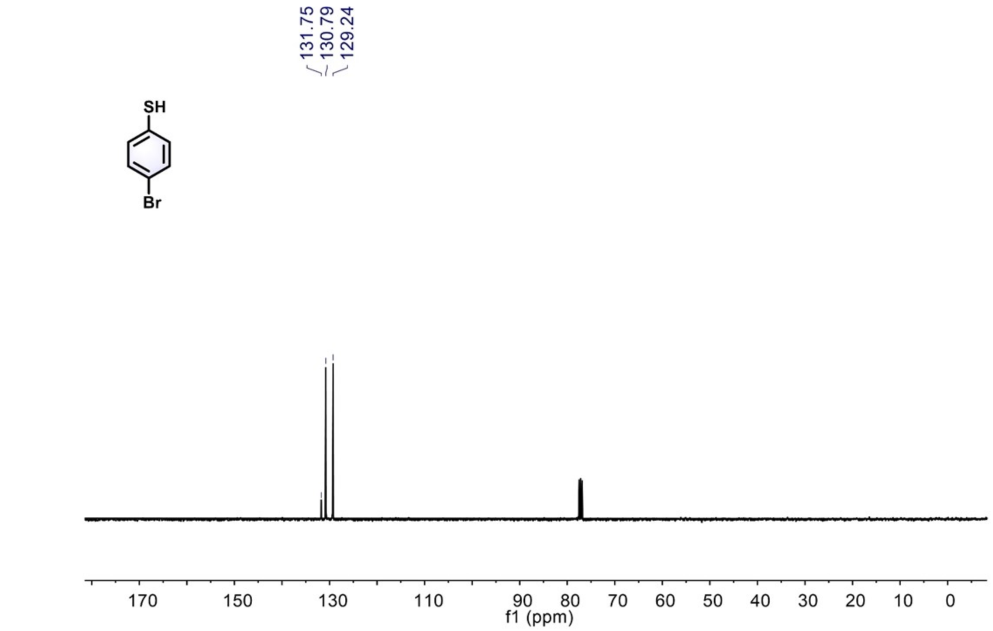

Figure S28. The ^{13}C NMR spectra of ***p*-Br** in $CDCl_3$. ^{13}C NMR (101 MHz, $CDCl_3$) δ 131.75 (s), 130.79 (s), 129.24 (s).

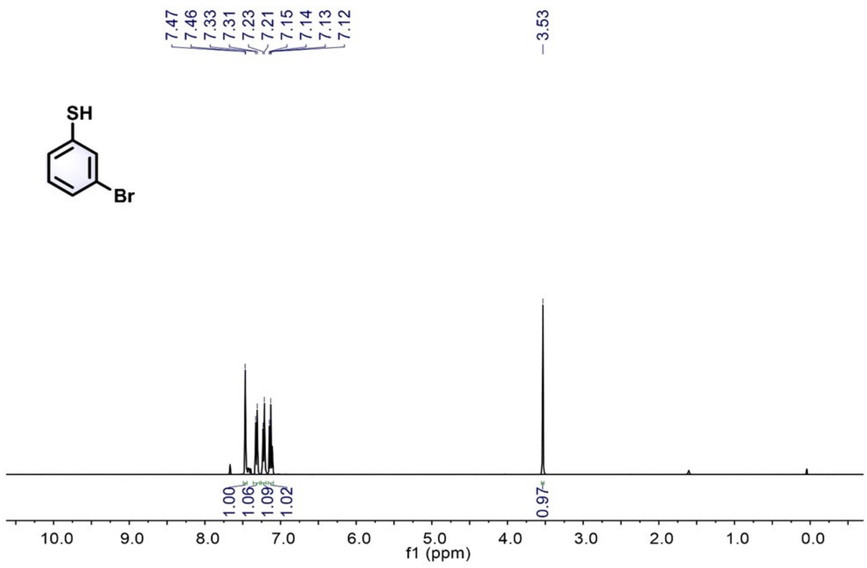

Figure S29. The ^{1}H NMR spectra of ***m*-Br** in $CDCl_3$. ^{1}H NMR (400 MHz, $CDCl_3$) δ 7.47 (d, J = 1.5 Hz, 1H), 7.32 (d, J = 7.9 Hz, 1H), 7.22 (d, J = 7.8 Hz, 1H), 7.14 (dd, J = 7.9, 2.0 Hz, 1H), 3.53 (s, 1H).

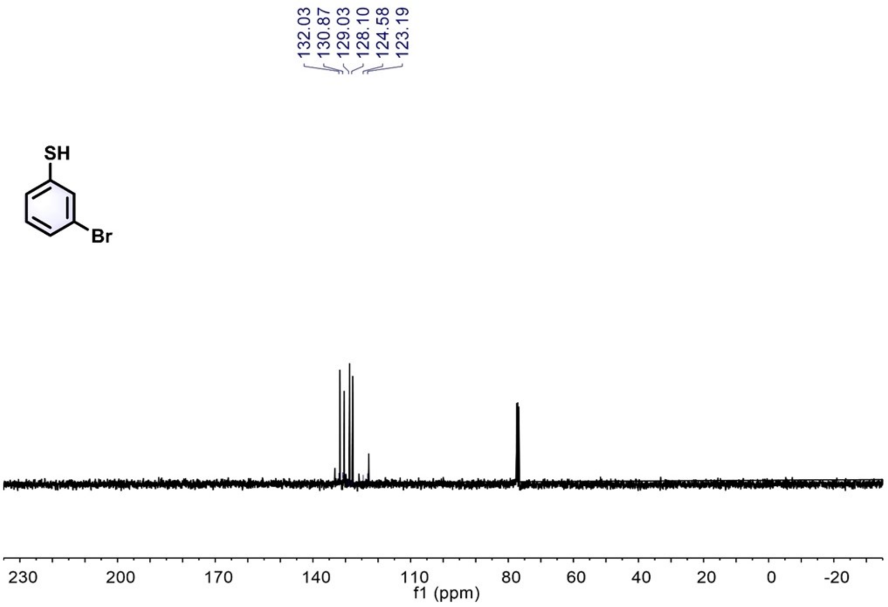

Figure S30. The ^{13}C NMR spectra of ***m*-Br** in $CDCl_3$. ^{13}C NMR (101 MHz, $CDCl_3$) δ 132.03 (s), 129.13 – 128.94 (m), 123.19 (s).

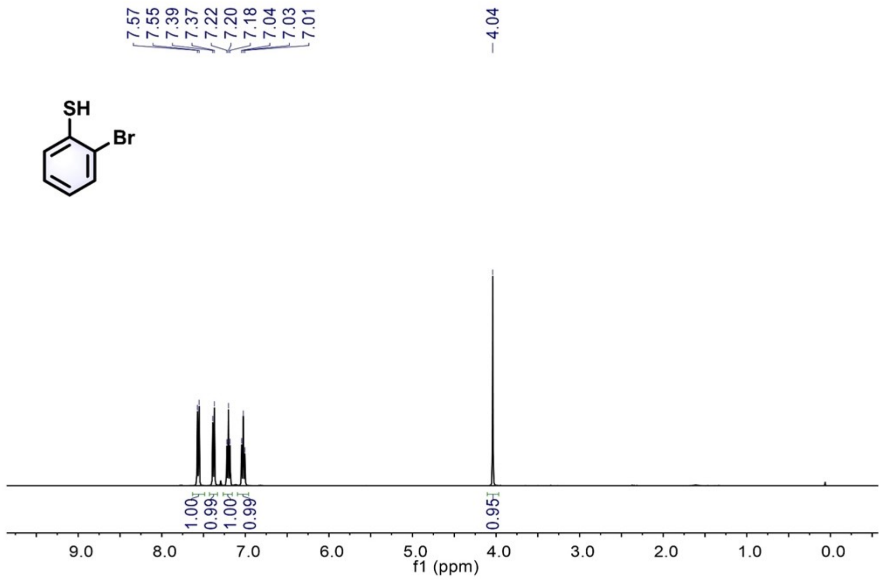

Figure S31. The ^{1}H NMR spectra of ***o*-Br** in $CDCl_3$. ^{1}H NMR (400 MHz, $CDCl_3$) δ 7.56 (d, J = 8.0 Hz, 1H), 7.38 (d, J = 7.8 Hz, 1H), 7.20 (t, J = 7.6 Hz, 1H), 7.03 (t, J = 7.7 Hz, 1H), 4.04 (s, 1H).

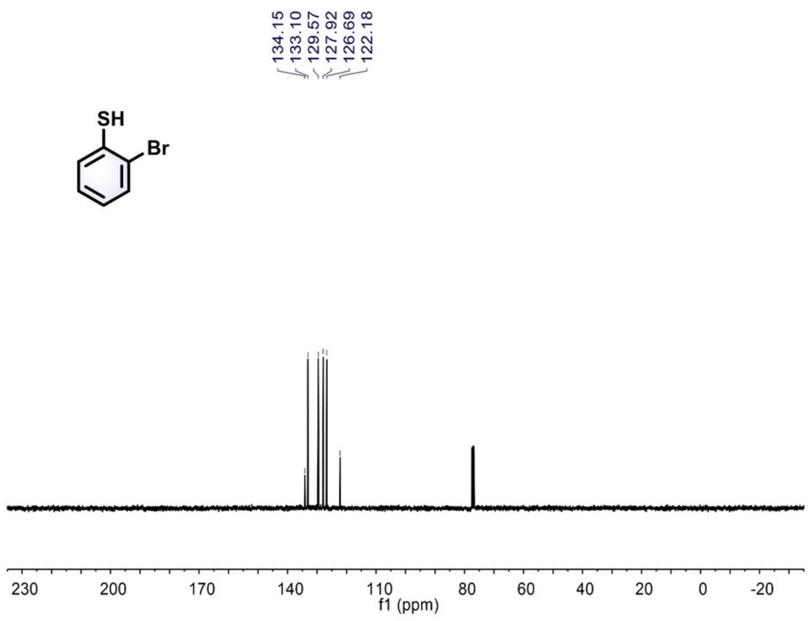

Figure S32. The ^{13}C NMR spectra of ***o*-Br** in $CDCl_3$. ^{13}C NMR (101 MHz, $CDCl_3$) δ 134.15 (s), 133.10 (s), 129.57 (s), 127.92 (s), 126.69 (s), 122.18 (s).

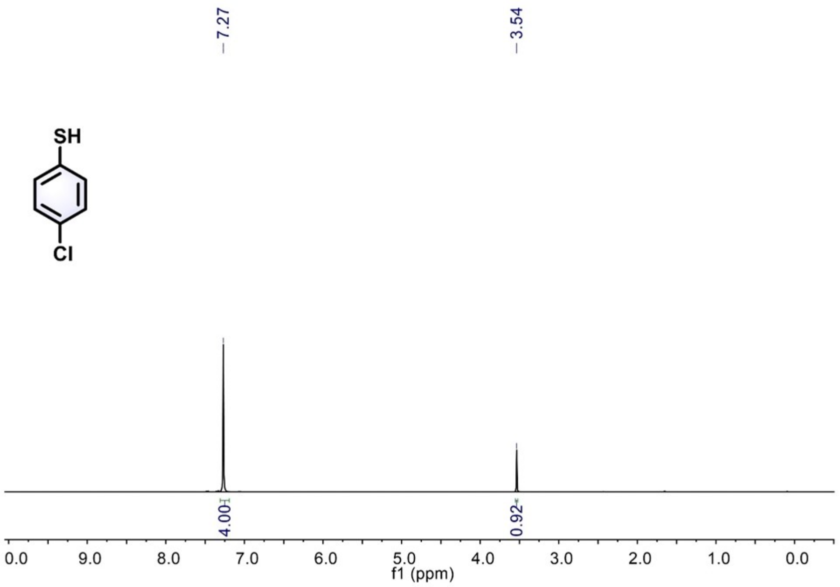

Figure S33. The ^{1}H NMR spectra of ***p*-Cl** in $CDCl_3$. ^{1}H NMR (400 MHz, $CDCl_3$) δ 7.27 (s, 4H), 3.54 (s, 1H).

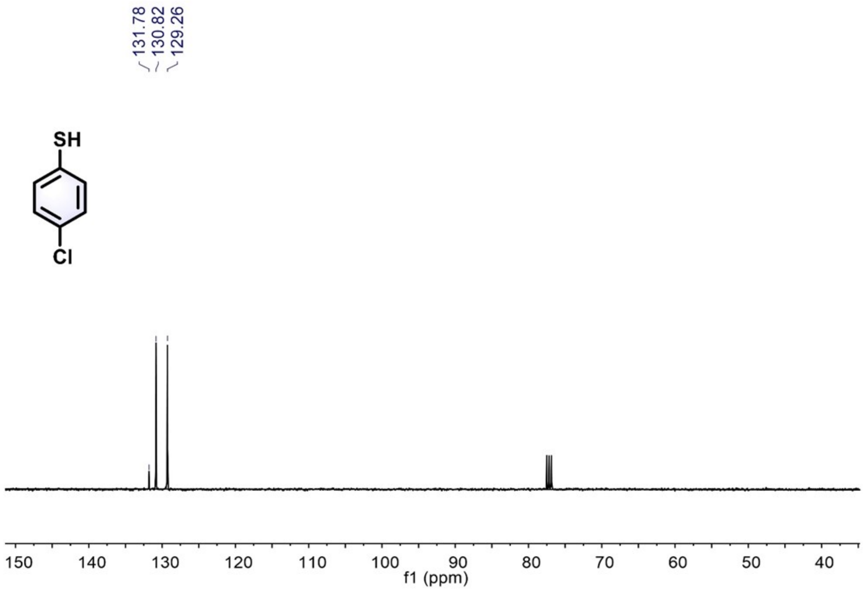

Figure S34. The ^{13}C NMR spectra of ***p*-Cl** in $CDCl_3$. ^{13}C NMR (101 MHz, $CDCl_3$) δ 131.78 (s), 130.82 (s), 129.26 (s).

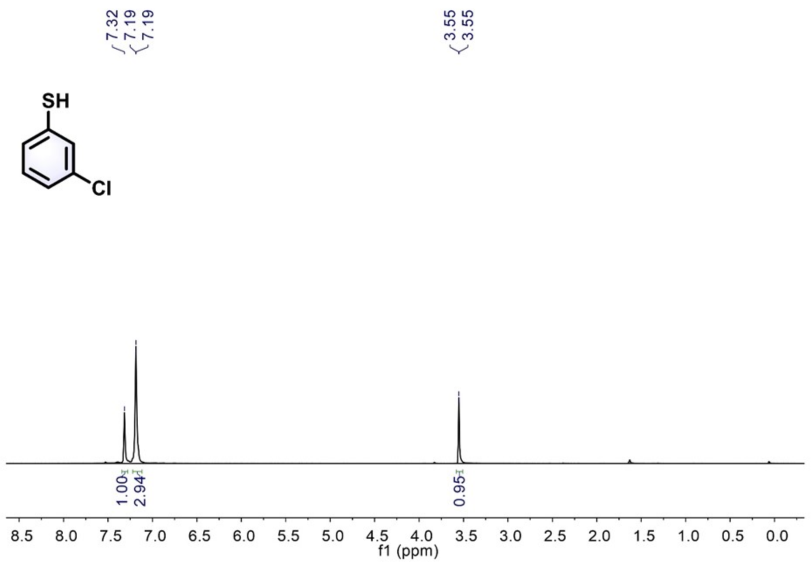

Figure S35. The ^{1}H NMR spectra of ***m*-Cl** in $CDCl_3$. ^{13}C NMR (101 MHz, $CDCl_3$) δ 134.74 (s), 132.97 (s), 130.14 (s), 128.93 (s), 127.33 (s), 125.85 (s).

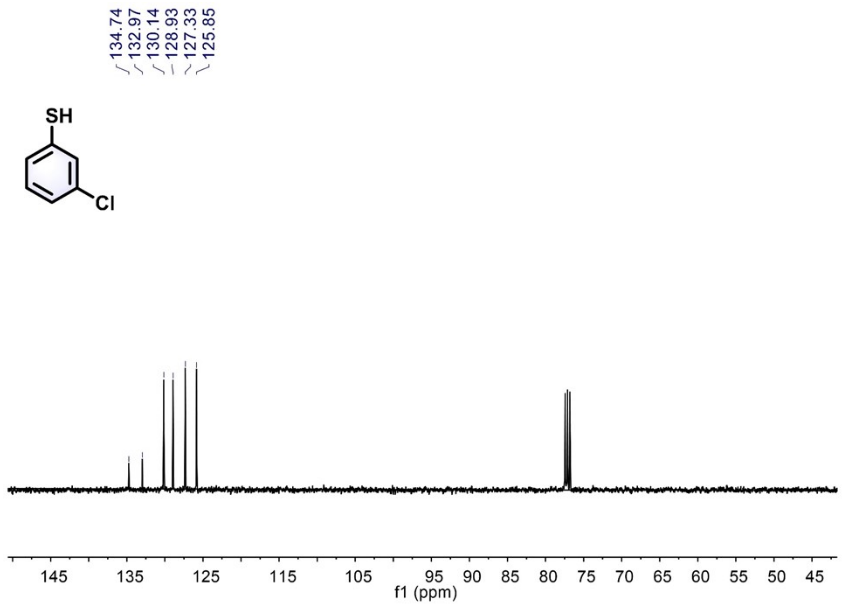

Figure S36. The ^{13}C NMR spectra of ***m*-Cl** in $CDCl_3$. ^{1}H NMR (400 MHz, $CDCl_3$) δ 7.39 (dd, J = 12.8, 7.7 Hz, 2H), 7.15 (dt, J = 18.9, 7.4 Hz, 2H), 3.95 (s, 1H).

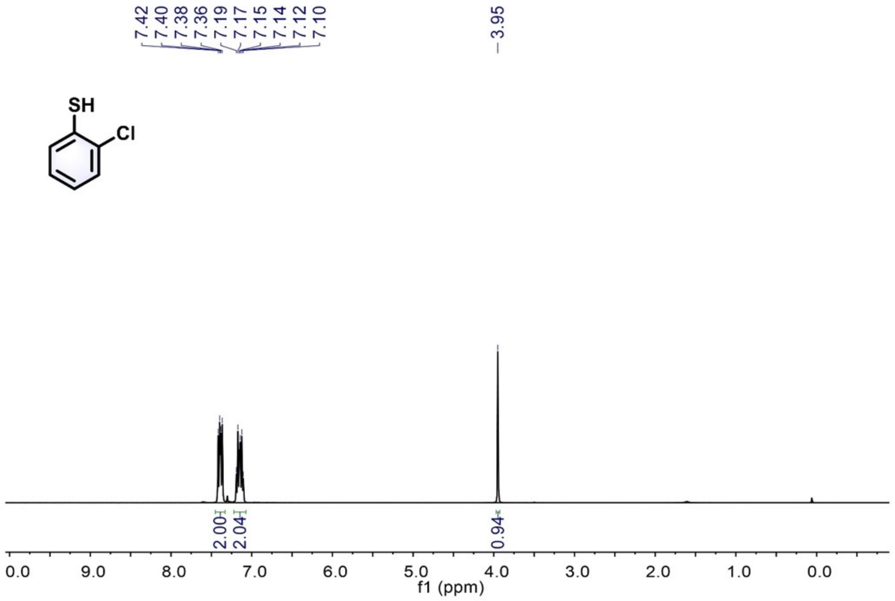

Figure S37. The ^{1}H NMR spectra of ***o*-Cl** in $CDCl_3$. ^{13}C NMR (101 MHz, $CDCl_3$) δ 131.84 (s), 129.78 (d, J = 4.0 Hz), 127.24 (s), 126.58 (s).

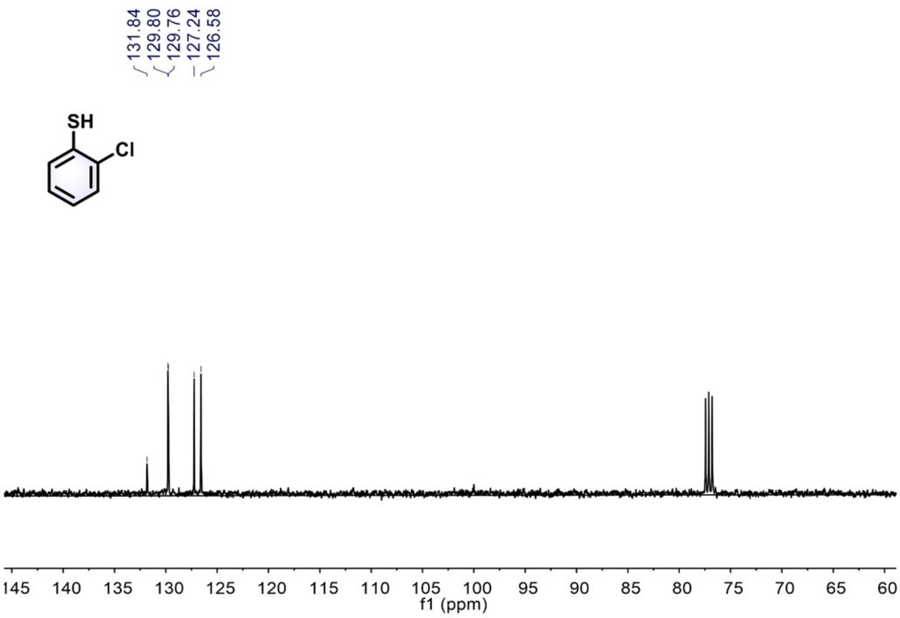

Figure S38. The ^{13}C NMR spectra of ***o*-Cl** in $CDCl_3$. ^{13}C NMR (101 MHz, $CDCl_3$) δ 131.84 (s), 129.78 (d, J = 4.0 Hz), 127.24 (s), 126.58 (s).

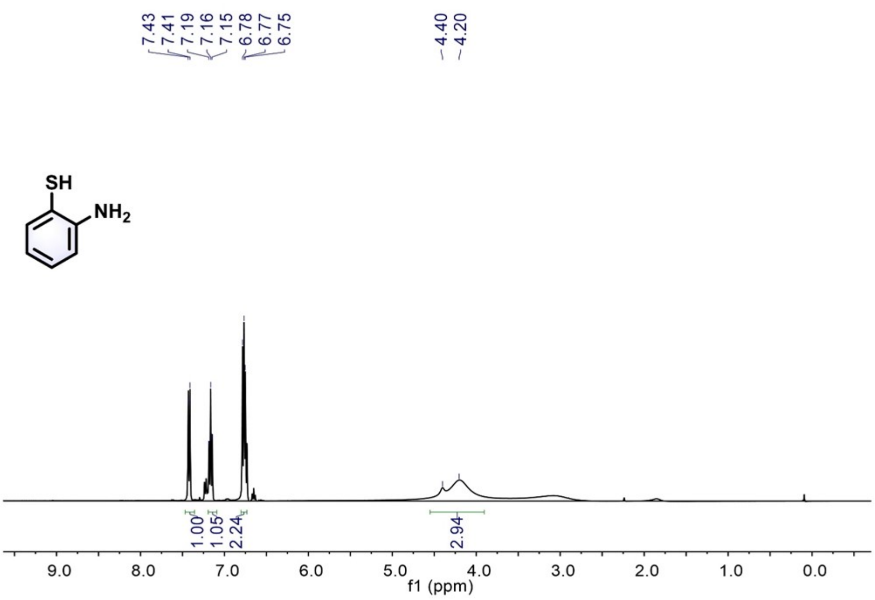

Figure S39. The ^{1}H NMR spectra of ***o*-NH₂** in $CDCl_3$. ^{1}H NMR (400 MHz, $CDCl_3$) δ 7.42 (d, J = 8.9 Hz, 1H), 7.17 (t, J = 7.7 Hz, 1H), 6.80 – 6.73 (m, 2H), 4.30 (d, J = 79.4 Hz, 3H).

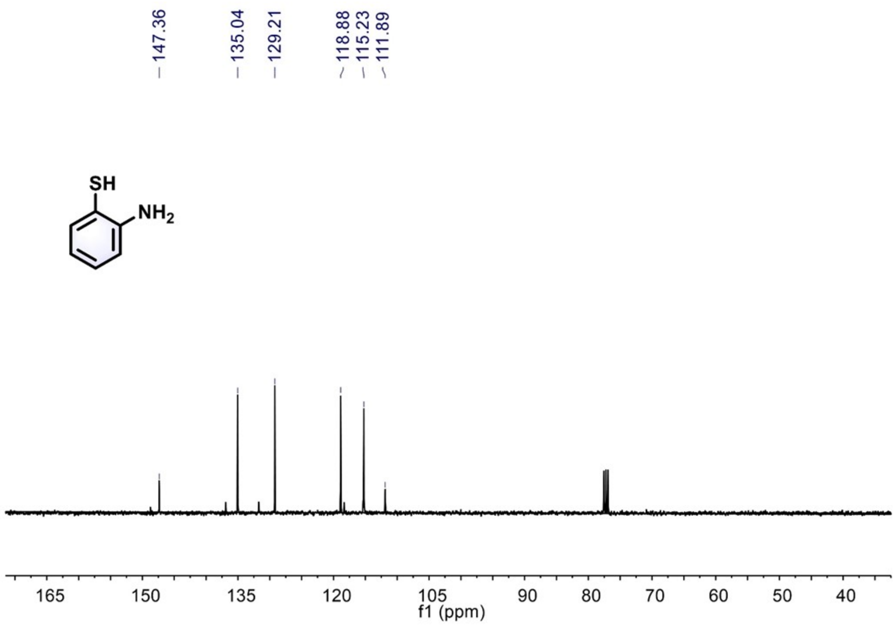

Figure S40. The ^{13}C NMR spectra of ***o*-NH$_2$** in CDCl$_3$. ^{13}C NMR (101 MHz, CDCl$_3$) δ 147.36 (s), 135.04 (s), 129.21 (s), 118.88 (s), 115.23 (s), 111.89 (s).

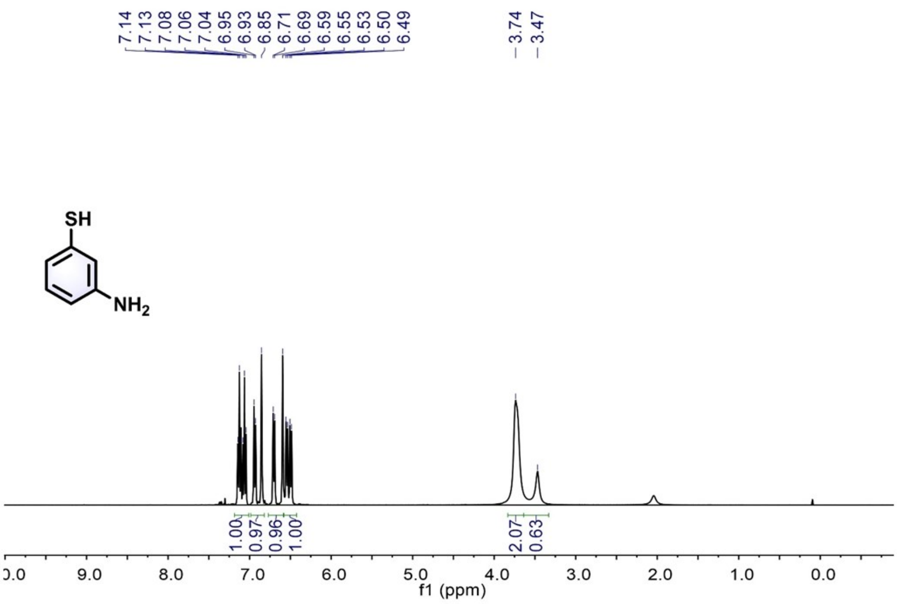

Figure S41. The ^{1}H NMR spectra of ***m*-NH$_2$** in CDCl$_3$. ^{1}H NMR (400 MHz, CDCl$_3$) δ 7.19 – 7.01 (m, 1H), 6.99 – 6.82 (m, 1H), 6.77 – 6.58 (m, 1H), 6.52 (dd, J = 19.1, 8.0 Hz, 1H), 3.74 (s, 2H), 3.47 (s, 1H).

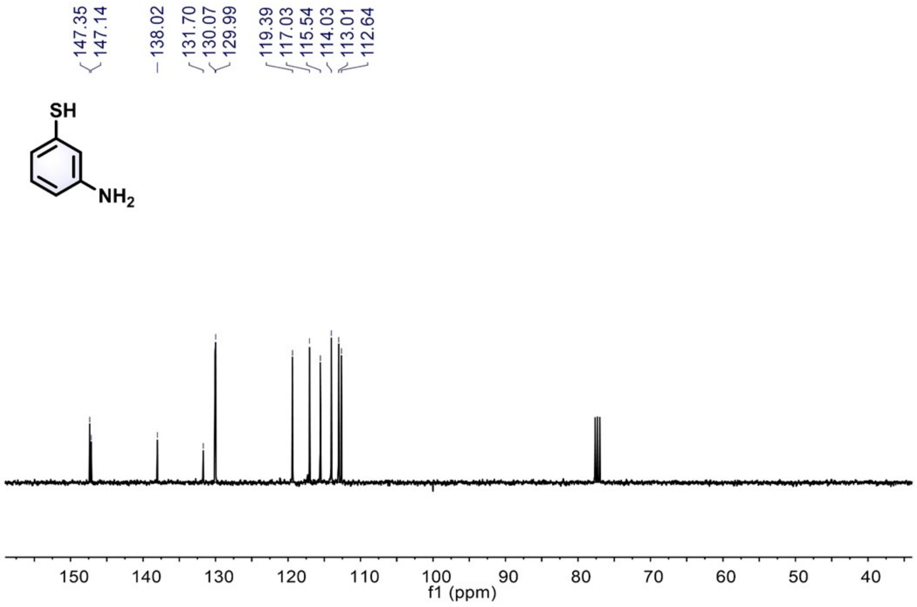

Figure S42. The ^{13}C NMR spectra of ***m*-NH$_2$** in $CDCl_3$. ^{13}C NMR (101 MHz, $CDCl_3$) δ 147.35 (s), 147.14 (s), 138.02 (s), 131.70 (s), 130.03 (d, J = 7.9 Hz), 119.39 (s), 117.03 (s), 115.54 (s), 114.03 (s), 113.01 (s), 112.64 (s).

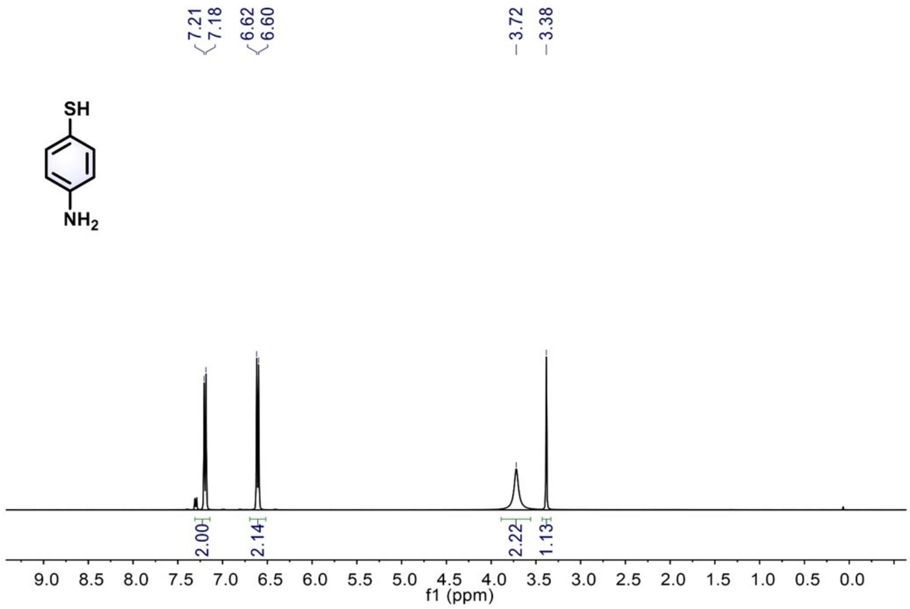

Figure S43. The ^{1}H NMR spectra of ***p*-NH₂** in $CDCl_3$. ^{1}H NMR (400 MHz, $CDCl_3$) δ 7.20 (d, J = 8.4 Hz, 2H), 6.61 (d, J = 8.4 Hz, 2H), 3.72 (s, 2H), 3.38 (s, 1H).

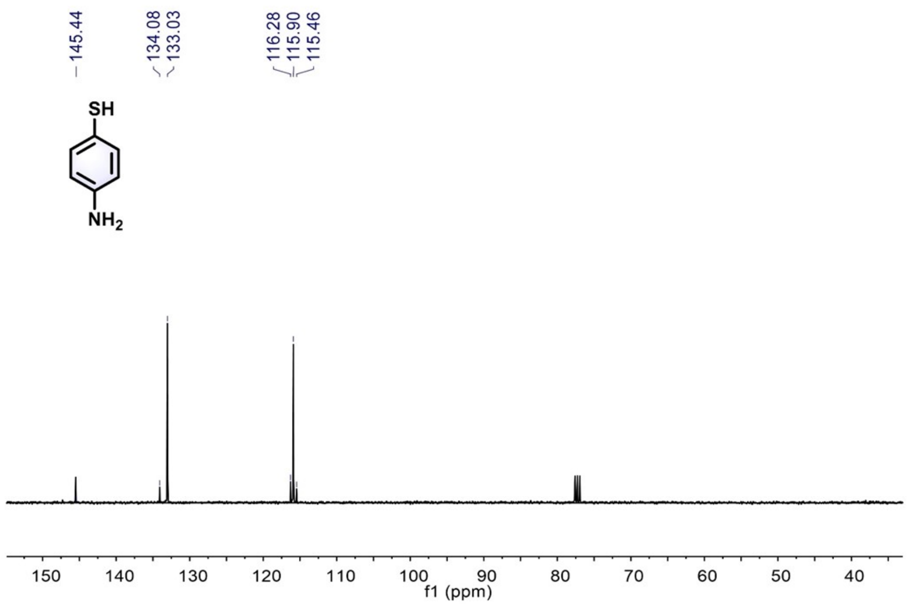

Figure S44. The ^{13}C NMR spectra of ***p*-NH₂** in $CDCl_3$. ^{13}C NMR (101 MHz, $CDCl_3$) δ 145.44 (s), 134.08 (s), 133.03 (s), 116.28 (s), 115.90 (s), 115.46 (s).

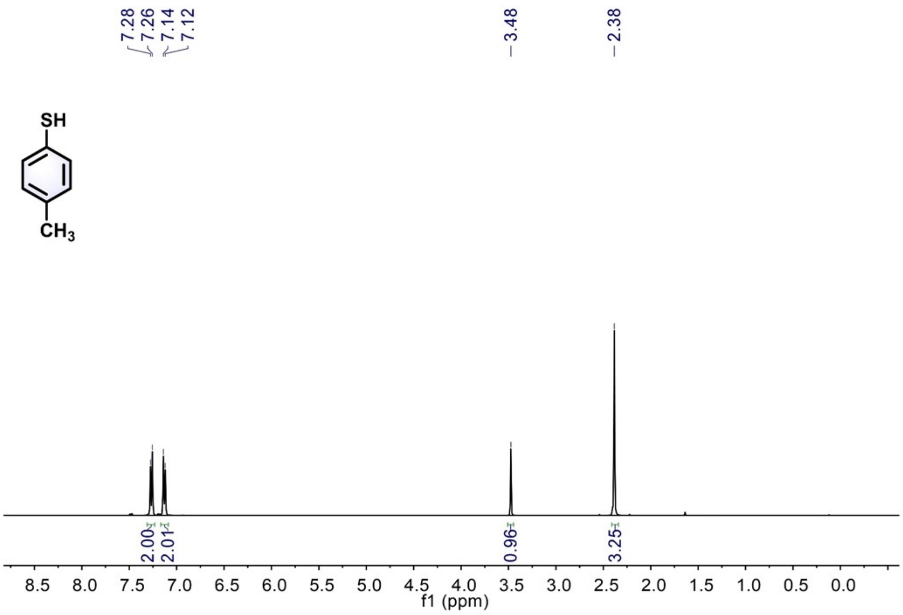

Figure S45. The ^{1}H NMR spectra of ***p*-CH$_3$** in CDCl$_3$. ^{1}H NMR (400 MHz, CDCl$_3$) δ 7.27 (d, J = 8.0 Hz, 2H), 7.13 (d, J = 7.9 Hz, 2H), 3.48 (s, 1H), 2.38 (s, 3H).

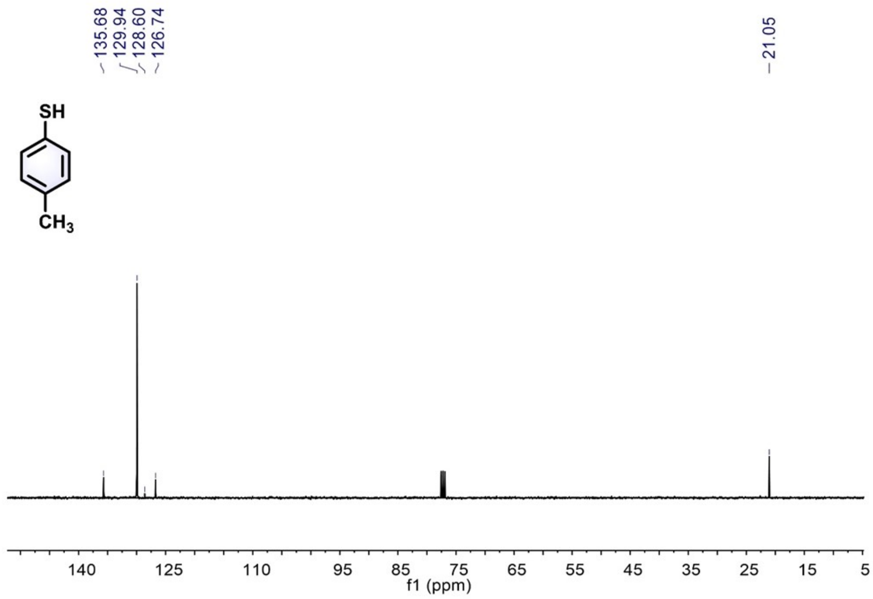

Figure S46. The ^{13}C NMR spectra of ***p*-CH₃** in $CDCl_3$. ^{13}C NMR (101 MHz, $CDCl_3$) δ 135.68 (s), 129.94 (s), 126.74 (s), 21.05 (s).

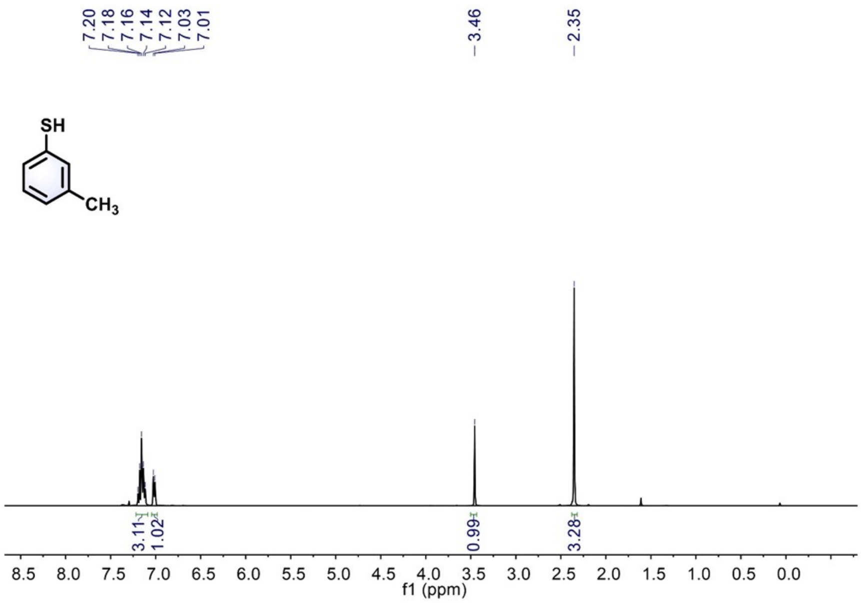

Figure S47. The ^{1}H NMR spectra of ***m*-CH$_3$** in $CDCl_3$. ^{1}H NMR (400 MHz, $CDCl_3$) δ 7.16 (p, J = 7.6 Hz, 3H), 7.02 (d, J = 7.3 Hz, 1H), 3.46 (s, 1H), 2.35 (s, 3H).

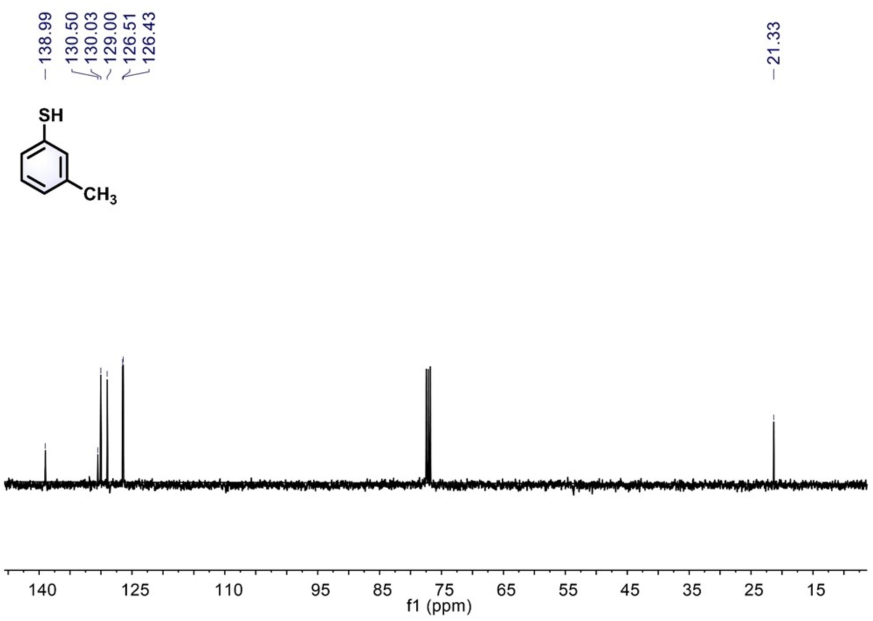

Figure S48. The ^{13}C NMR spectra of ***m*-CH$_3$** in CDCl$_3$. ^{13}C NMR (101 MHz, CDCl$_3$) δ 138.99 (s), 130.50 (s), 130.03 (s), 129.00 (s), 126.47 (d, J = 8.4 Hz), 21.33 (s).

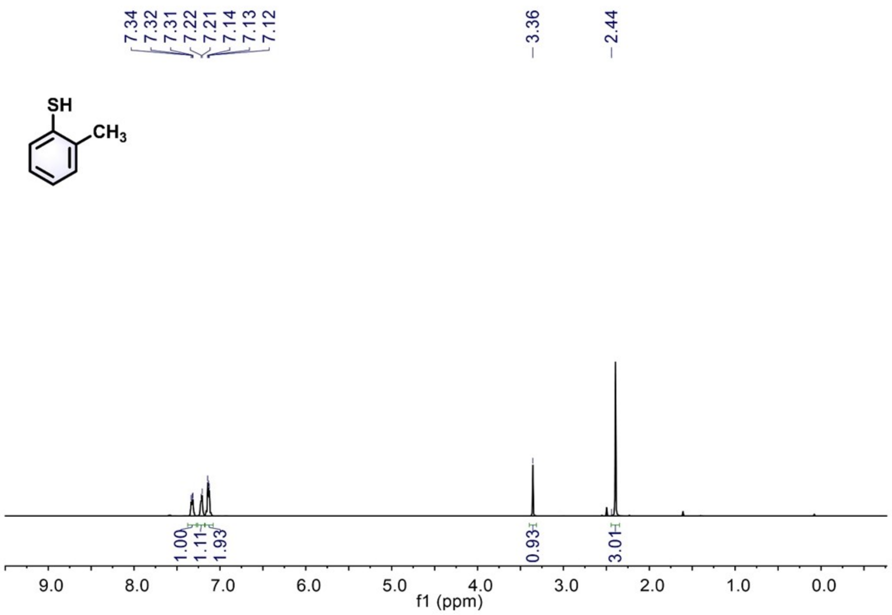

Figure S49. The ^{1}H NMR spectra of ***o*-CH$_3$** in $CDCl_3$. ^{1}H NMR (400 MHz, $CDCl_3$) δ 7.38 – 7.27 (m, 1H), 7.21 (d, J = 5.2 Hz, 1H), 7.17 – 7.08 (m, 2H), 3.36 (s, 1H), 2.44 (s, 3H).

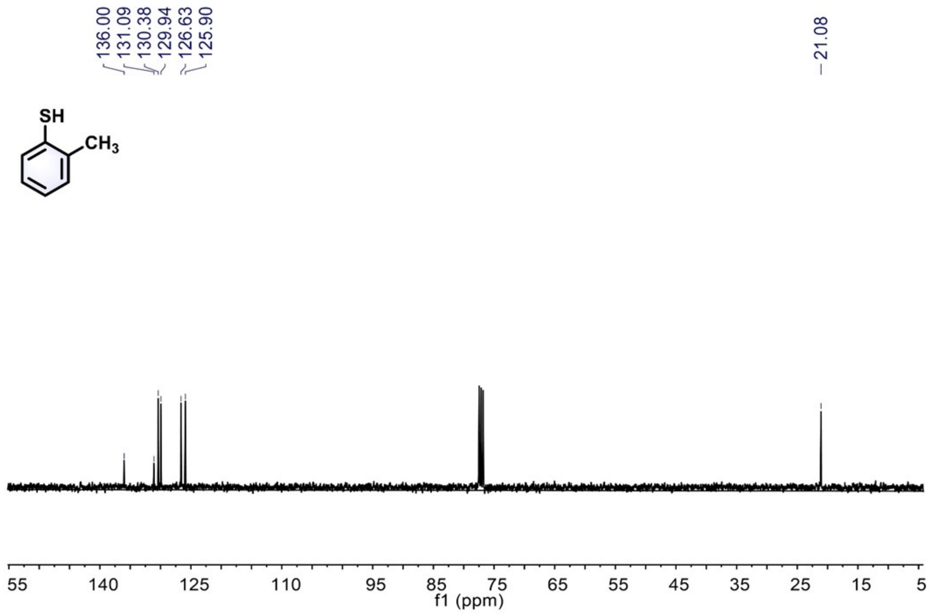

Figure S50. The ^{13}C NMR spectra of ***o*-CH₃** in $CDCl_3$. ^{13}C NMR (101 MHz, $CDCl_3$) δ 136.00 (s), 131.09 (s), 130.38 (s), 129.94 (s), 126.63 (s), 125.90 (s), 21.08 (s).

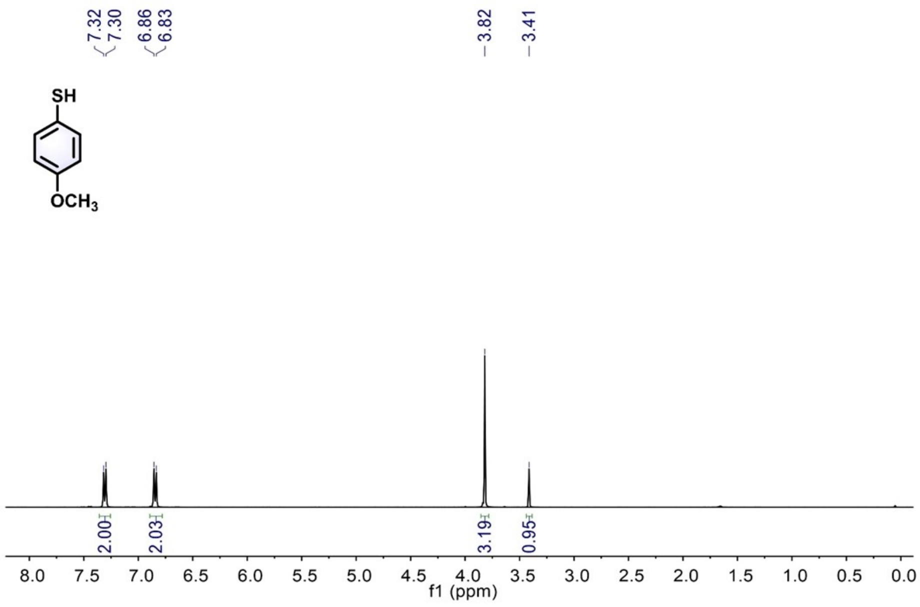

Figure S51. The ^{1}H NMR spectra of ***p*-MTP** in $CDCl_3$. ^{1}H NMR (400 MHz, $CDCl_3$) δ 7.31 (d, J = 8.7 Hz, 2H), 6.84 (d, J = 8.5 Hz, 2H), 3.82 (s, 3H), 3.41 (s, 1H).

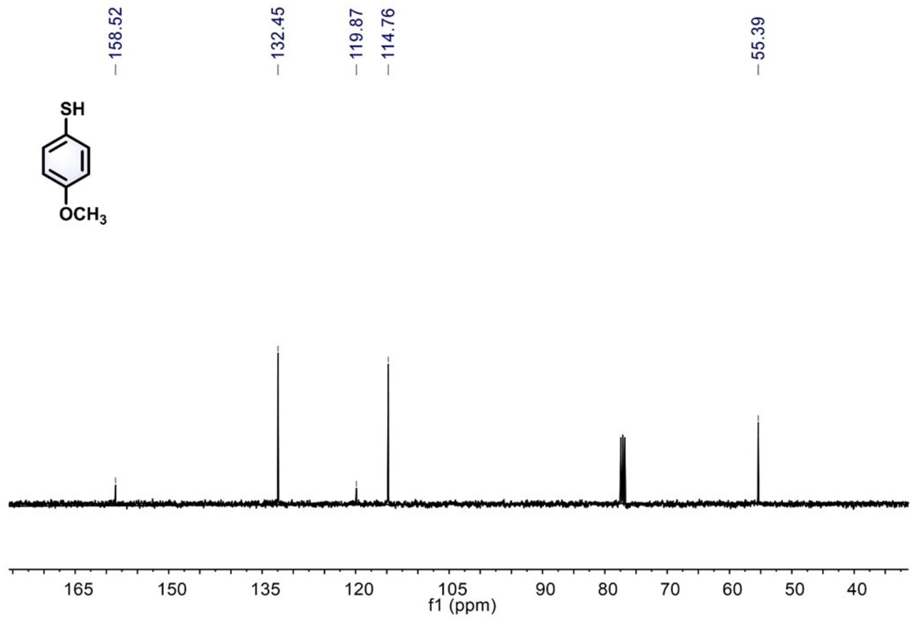

Figure S52. The ^{13}C NMR spectra of ***p*-MTP** in $CDCl_3$. ^{13}C NMR (101 MHz, $CDCl_3$) δ 158.52 (s), 132.45 (s), 119.87 (s), 114.76 (s), 55.39 (s).

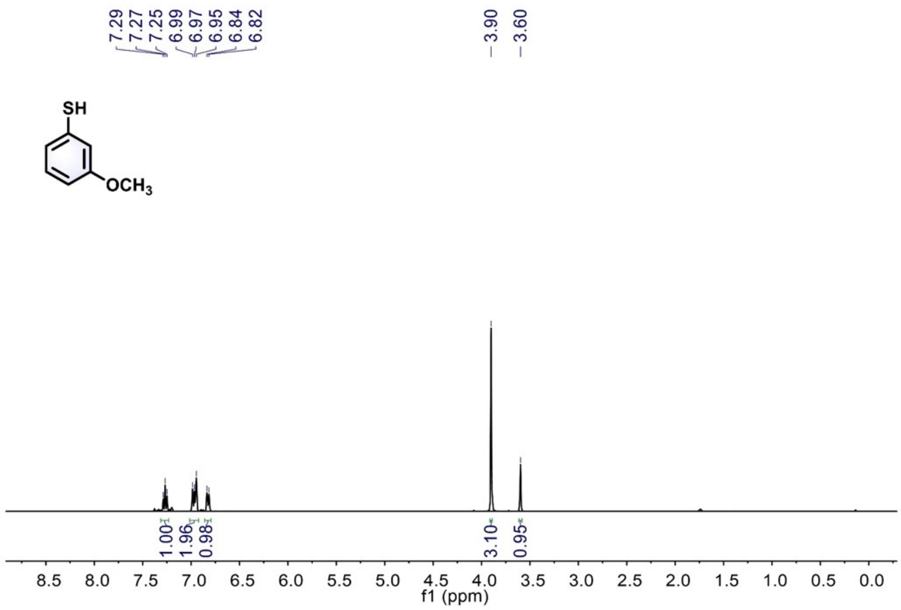

Figure S53. The ^{1}H NMR spectra of ***m*-MTP** in $CDCl_3$. ^{1}H NMR (400 MHz, $CDCl_3$) δ 7.27 (t, J = 8.0 Hz, 1H), 7.01 – 6.92 (m, 2H), 6.83 (d, J = 8.3 Hz, 1H), 3.90 (s, 3H), 3.60 (s, 1H).

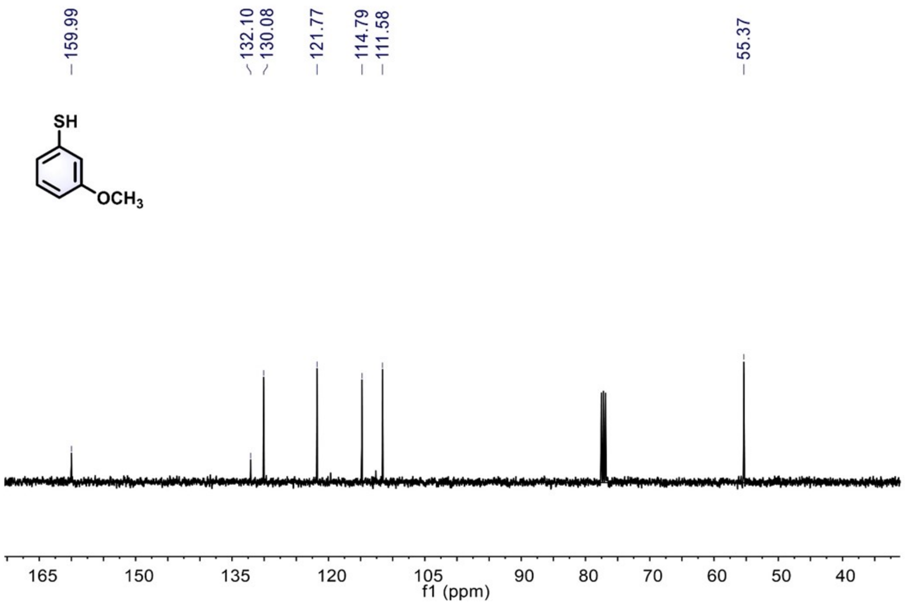

Figure S54. The ^{13}C NMR spectra of ***m*-MTP** in $CDCl_3$. ^{13}C NMR (101 MHz, $CDCl_3$) δ 159.99 (s), 132.10 (s), 130.08 (s), 121.77 (s), 114.79 (s), 111.58 (s), 55.37 (s).

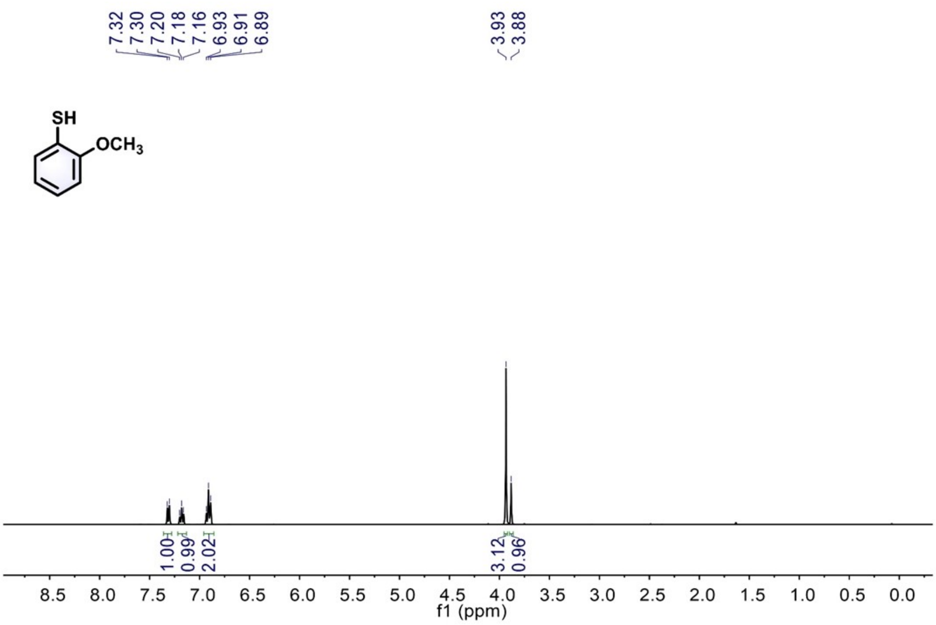

Figure S55. The ^{1}H NMR spectra of ***o*-MTP** in $CDCl_3$. ^{1}H NMR (400 MHz, $CDCl_3$) δ 7.31 (d, J = 8.5 Hz, 1H), 7.18 (t, J = 7.8 Hz, 1H), 6.91 (t, J = 8.5 Hz, 2H), 3.93 (s, 3H), 3.88 (s, 1H).

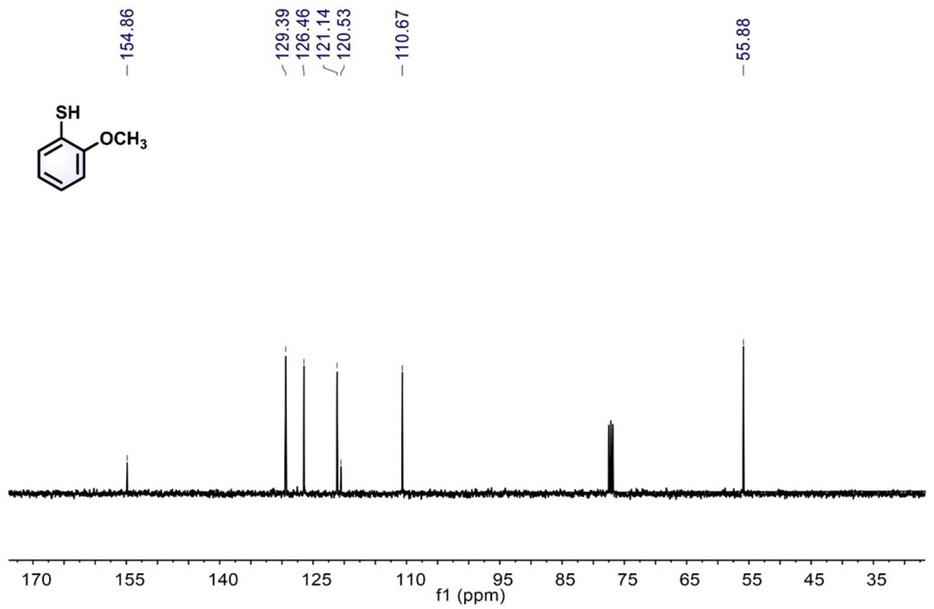

Figure S56. The ^{13}C NMR spectra of ***o*-MTP** in $CDCl_3$. ^{13}C NMR (101 MHz, $CDCl_3$) δ 154.86 (s), 129.39 (s), 126.46 (s), 121.14 (s), 120.53 (s), 110.67 (s), 55.88 (s).

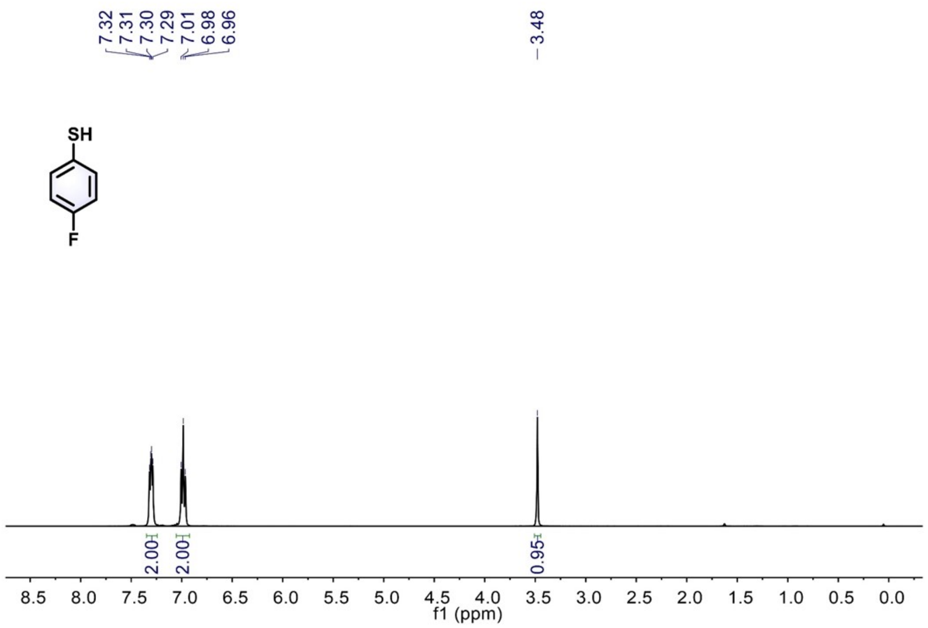

Figure S57. The ^{1}H NMR spectra of ***p*-FTP** in $CDCl_3$. ^{1}H NMR (400 MHz, $CDCl_3$) δ 7.30 (dd, J = 7.8, 5.2 Hz, 2H), 6.99 (t, J = 8.2 Hz, 2H), 3.48 (s, 1H).

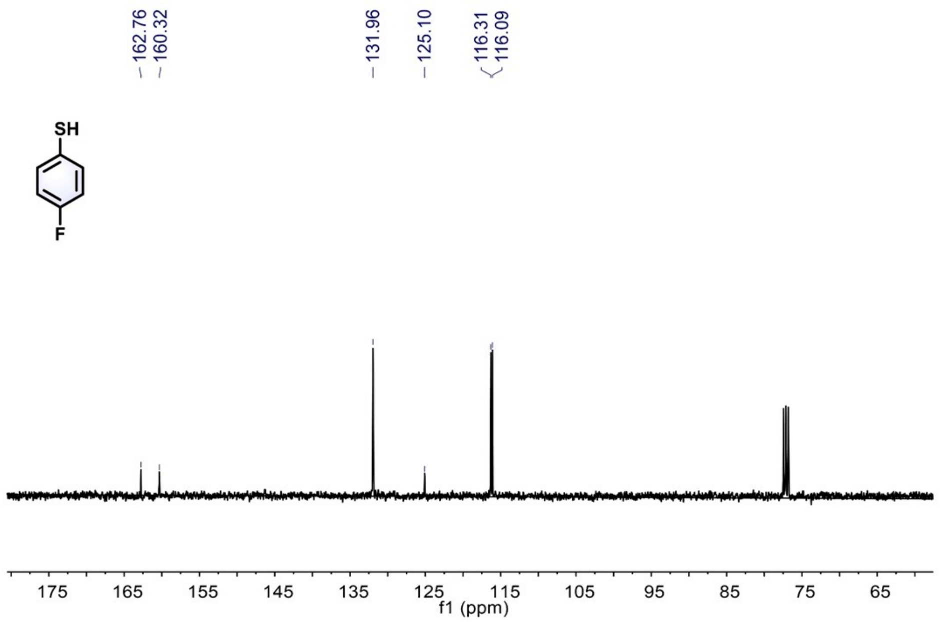

Figure S58. The ^{13}C NMR spectra of ***p*-FTP** in $CDCl_3$. ^{13}C NMR (101 MHz, $CDCl_3$) δ 162.76 (s), 160.32 (s), 131.96 (s), 125.10 (s), 116.31 (s), 116.09 (s).

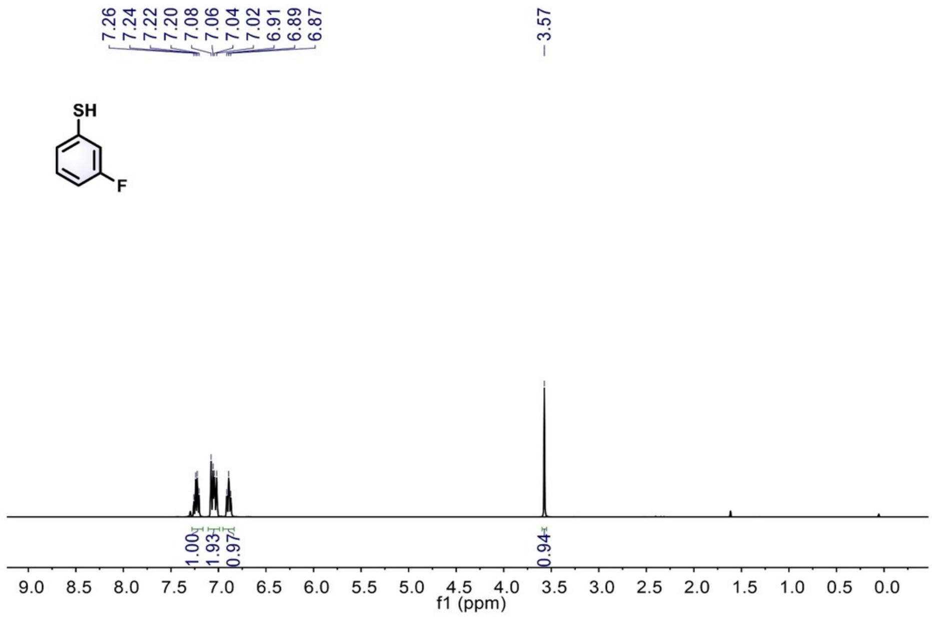

Figure S59. The ^{1}H NMR spectra of ***m*-FTP** in $CDCl_3$. ^{1}H NMR (400 MHz, $CDCl_3$) δ 7.23 (dd, J = 14.3, 7.8 Hz, 1H), 7.05 (dd, J = 14.7, 8.6 Hz, 2H), 6.89 (t, J = 8.4 Hz, 1H), 3.57 (s, 1H).

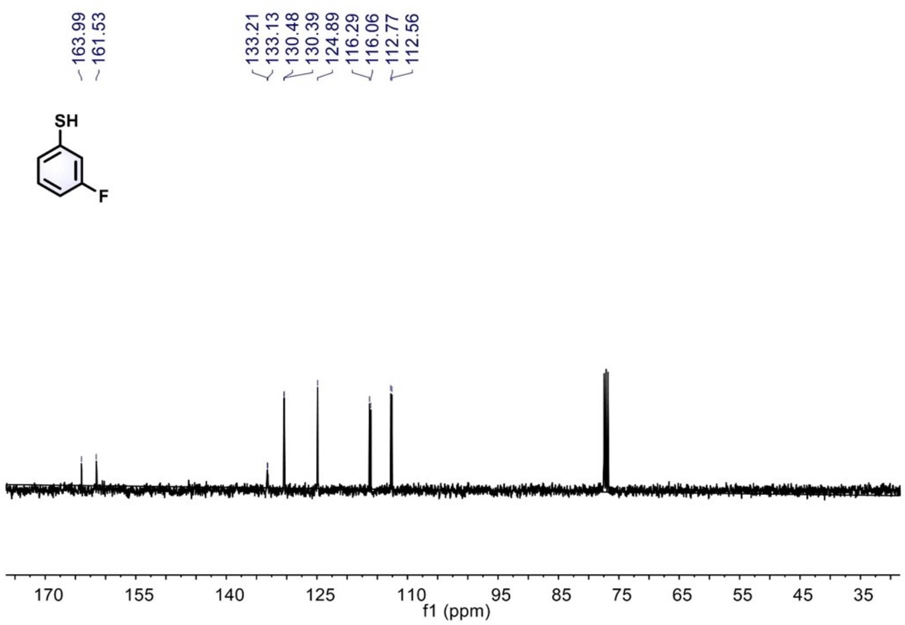

Figure S60. The ^{13}C NMR spectra of ***m*-FTP** in $CDCl_3$. ^{13}C NMR (101 MHz, $CDCl_3$) δ 163.99 (s), 161.53 (s), 133.17 (d, J = 8.2 Hz), 130.43 (d, J = 8.6 Hz), 124.89 (s), 116.29 (s), 116.06 (s), 112.77 (s), 112.56 (s).

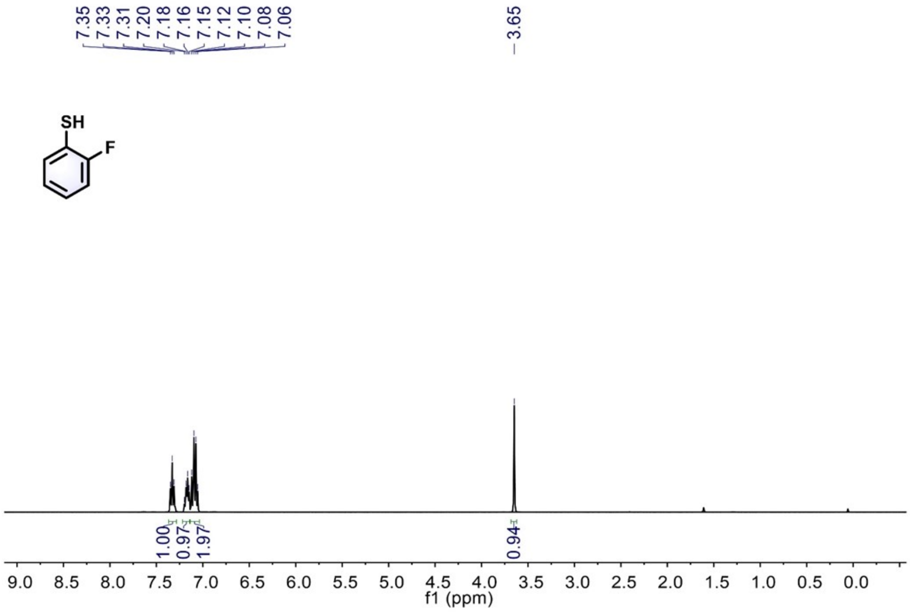

Figure S61. The ^{1}H NMR spectra of ***o*-FTP** in $CDCl_3$. ^{1}H NMR (400 MHz, $CDCl_3$) δ 7.33 (t, J = 7.7 Hz, 1H), 7.17 (dd, J = 13.7, 6.8 Hz, 1H), 7.14 – 7.04 (m, 2H), 3.65 (s, 1H).

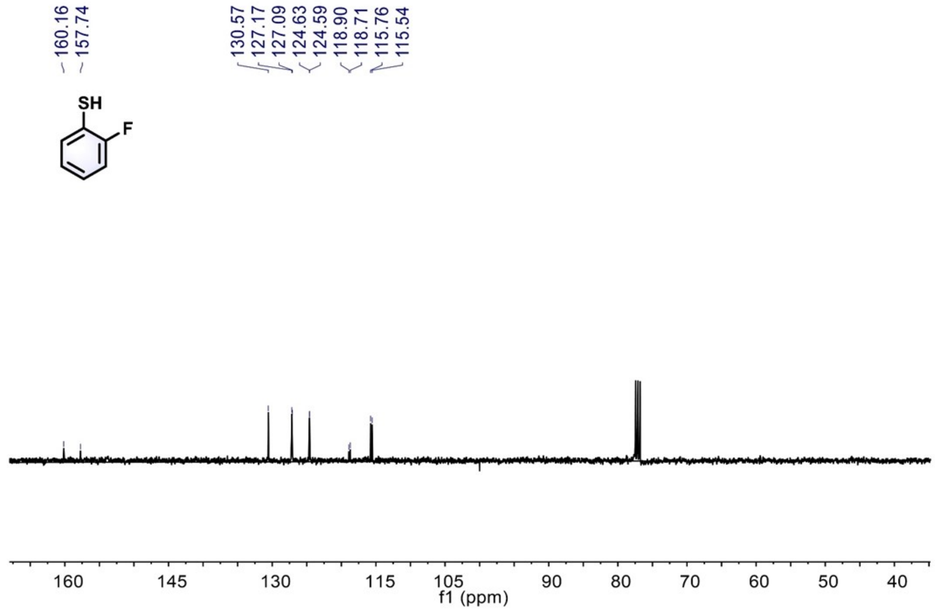

Figure S62. The ^{13}C NMR spectra of ***o*-FTP** in $CDCl_3$. ^{13}C NMR (101 MHz, $CDCl_3$) δ 160.16 (s), 157.74 (s), 130.57 (s), 127.13 (d, J = 7.5 Hz), 124.61 (d, J = 3.7 Hz), 118.81 (d, J = 19.9 Hz), 115.76 (s), 115.54 (s).

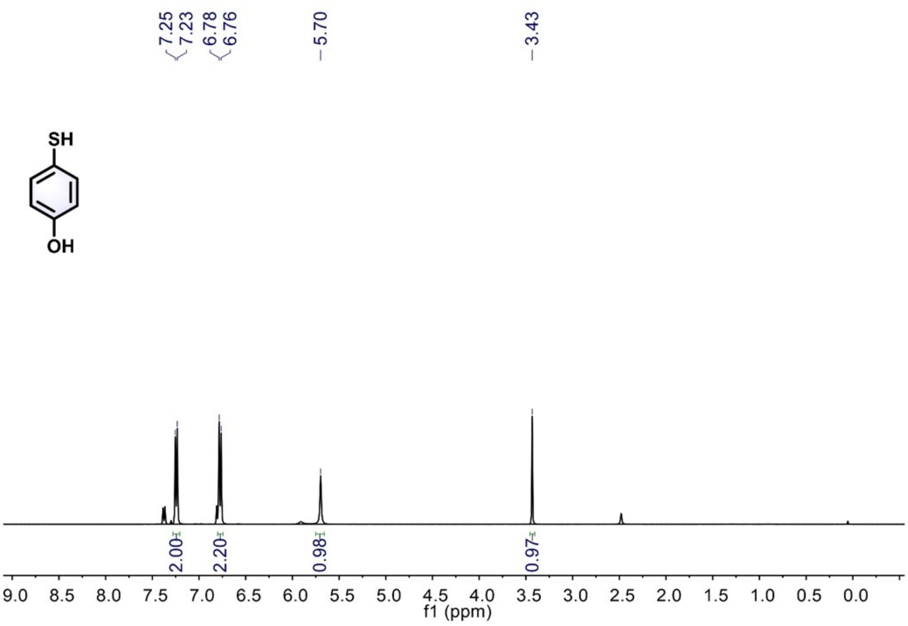

Figure S63. The ^{1}H NMR spectra of ***p*-OH** in $CDCl_3$. ^{1}H NMR (400 MHz, $CDCl_3$) δ 7.24 (d, J = 8.3 Hz, 2H), 6.77 (d, J = 8.2 Hz, 2H), 5.70 (s, 1H), 3.43 (s, 1H).

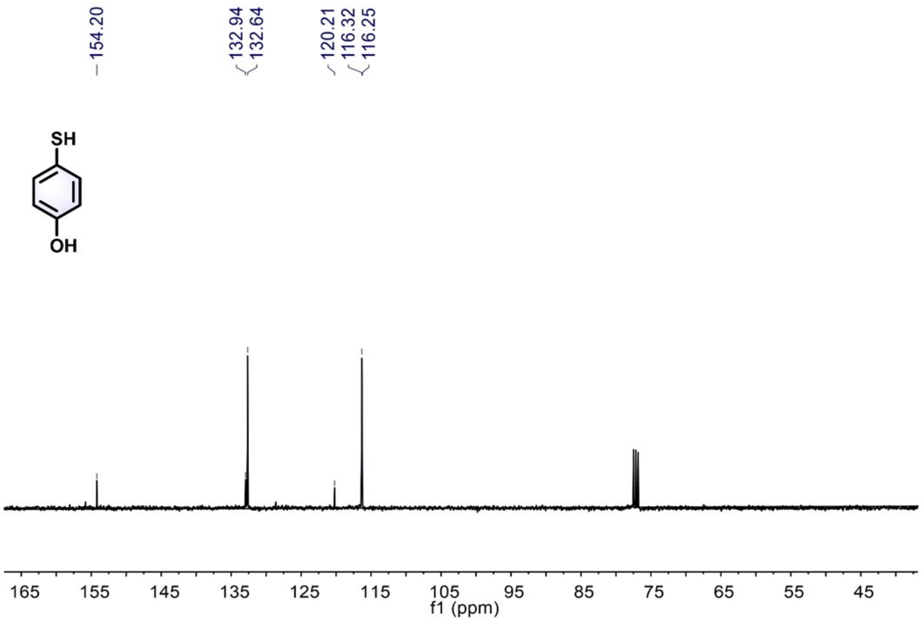

Figure S64. The ^{13}C NMR spectra of ***p*-OH** in $CDCl_3$. ^{13}C NMR (101 MHz, $CDCl_3$) δ 154.20 (s), 132.94 (s), 132.64 (s), 120.21 (s), 116.29 (d, J = 6.7 Hz).

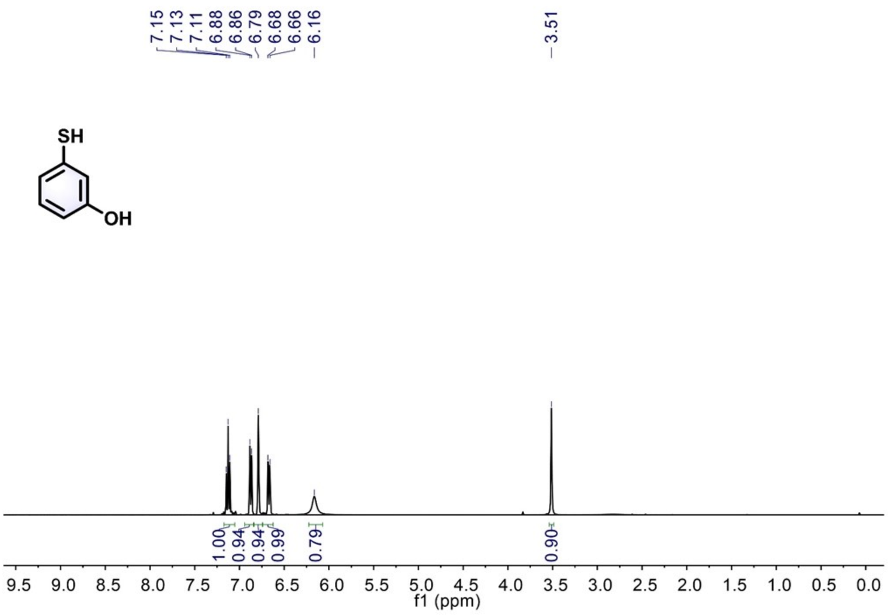

Figure S65. The ^{1}H NMR spectra of ***m*-OH** in $CDCl_3$. ^{1}H NMR (400 MHz, $CDCl_3$) δ 7.13 (t, J = 8.0 Hz, 1H), 6.87 (d, J = 7.7 Hz, 1H), 6.79 (s, 1H), 6.67 (d, J = 9.7 Hz, 1H), 6.16 (s, 1H), 3.51 (s, 1H).

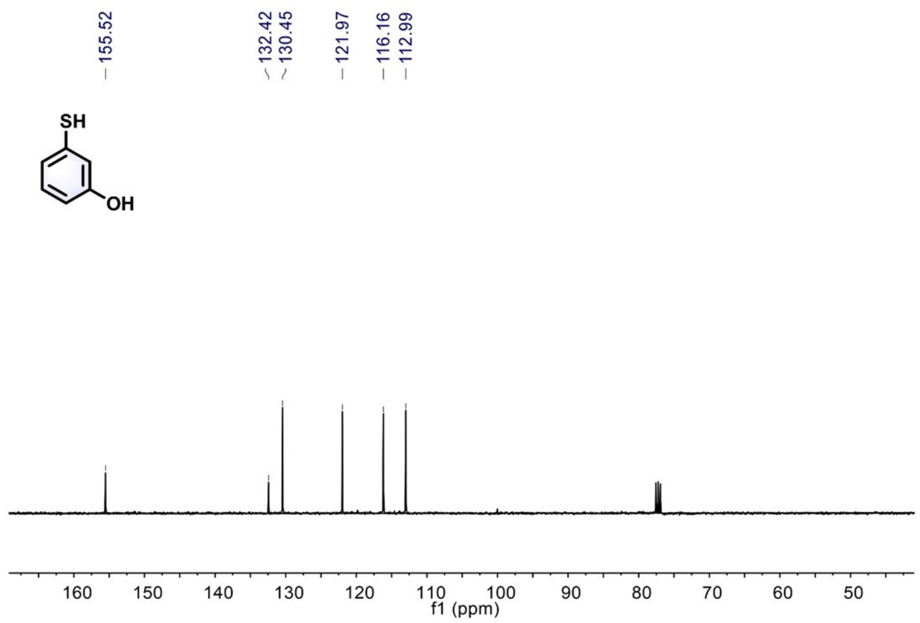

Figure S66. The ^{13}C NMR spectra of ***m*-OH** in $CDCl_3$. ^{13}C NMR (101 MHz, $CDCl_3$) δ 155.52 (s), 132.42 (s), 130.45 (s), 121.97 (s), 116.16 (s), 112.99 (s).

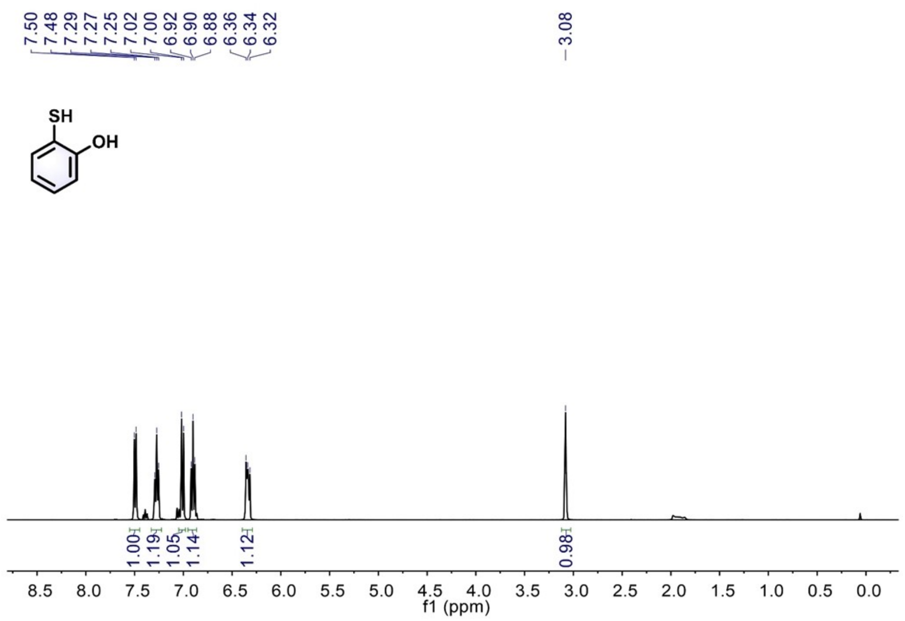

Figure S67. The ^{1}H NMR spectra of ***o*-OH** in $CDCl_3$. ^{1}H NMR (400 MHz, $CDCl_3$) δ 7.49 (d, J = 7.6 Hz, 1H), 7.27 (t, J = 7.7 Hz, 1H), 7.01 (d, J = 8.1 Hz, 1H), 6.90 (t, J = 7.6 Hz, 1H), 6.40 – 6.29 (m, 1H), 3.08 (s, 1H).

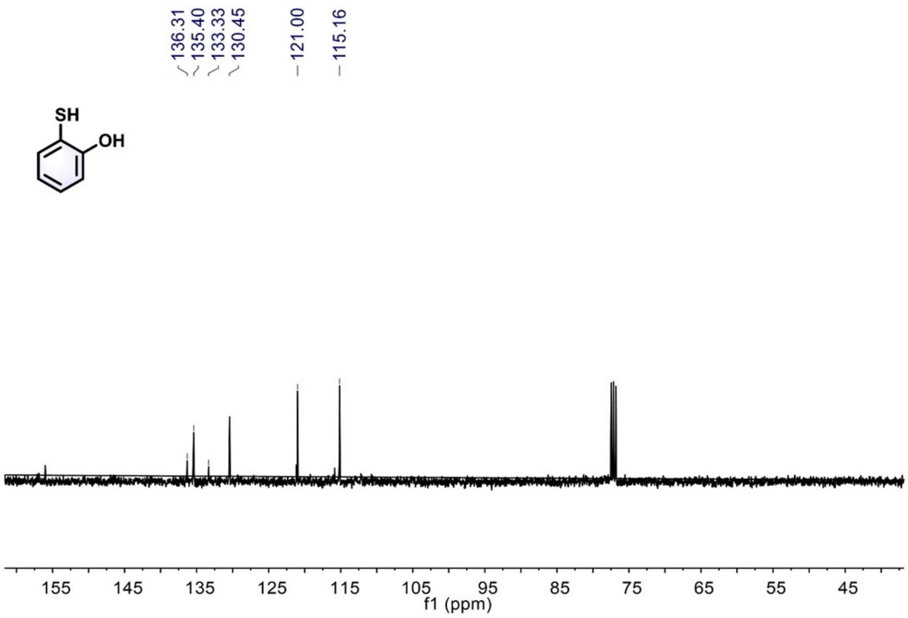

Figure S68. The ^{13}C NMR spectra of ***o*-OH** in $CDCl_3$. ^{13}C NMR (101 MHz, $CDCl_3$) δ 136.31 (s), 135.40 (s), 133.33 (s), 130.45 (s), 121.00 (s), 115.16 (s).

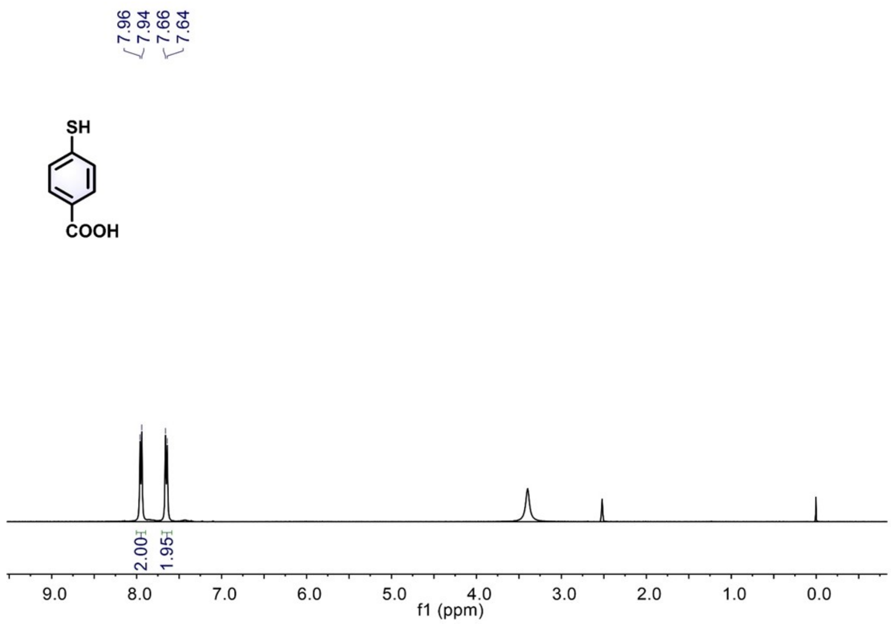

Figure S69. The ^1H NMR spectra of ***p*-COOH** in $CDCl_3$. ^1H NMR (400 MHz, DMSO) δ 7.95 (d, J = 8.2 Hz, 2H), 7.65 (d, J = 8.2 Hz, 2H).

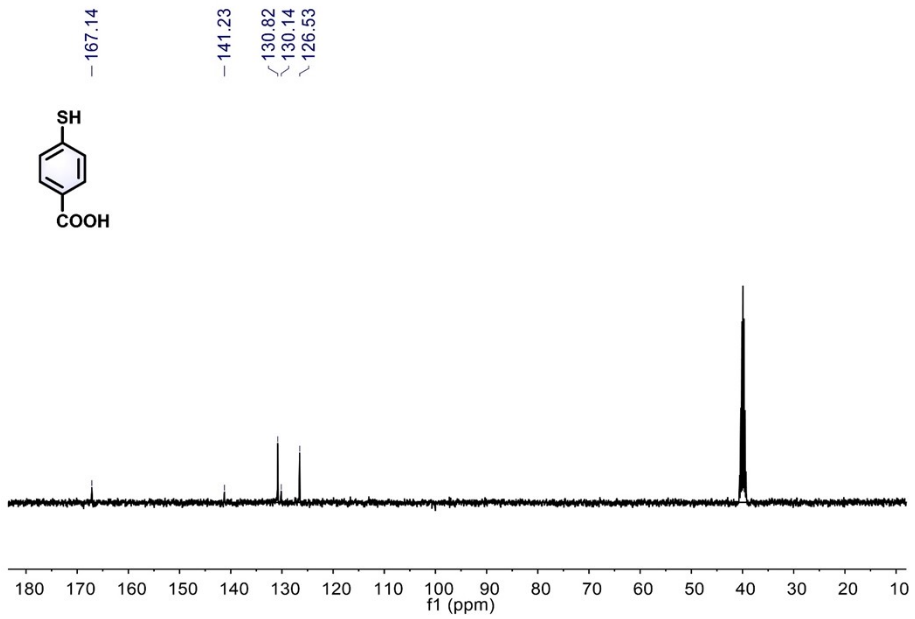

Figure S70. The ^{13}C NMR spectra of ***p*-COOH** in $CDCl_3$. ^{13}C NMR (101 MHz, DMSO) δ 167.14 (s), 141.23 (s), 130.82 (s), 130.14 (s), 126.53 (s).

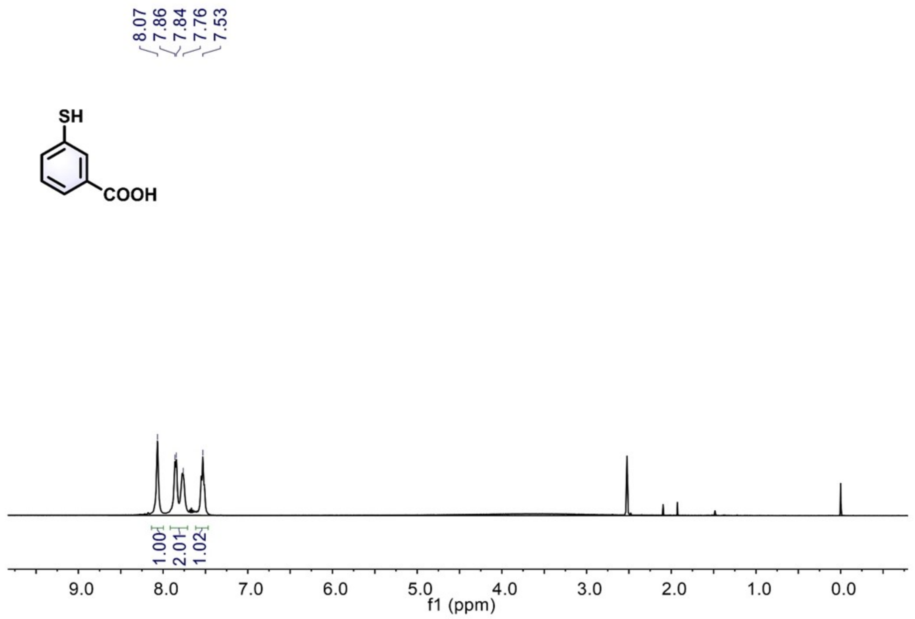

Figure S71. The ^{1}H NMR spectra of ***m*-COOH** in $CDCl_3$. ^{1}H NMR (400 MHz, DMSO) δ 8.07 (s, 1H), 7.92 – 7.71 (m, 2H), 7.53 (s, 1H).

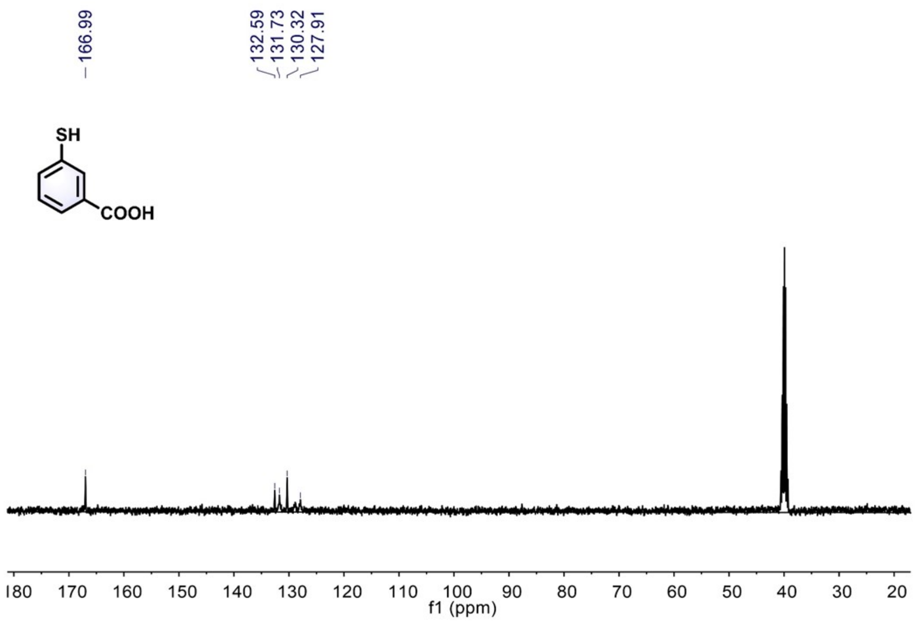

Figure S72. The ^{13}C NMR spectra of ***m*-COOH** in $CDCl_3$. ^{13}C NMR (101 MHz, DMSO) δ 166.99 (s), 132.59 (s), 131.73 (s), 130.32 (s), 127.91 (s).

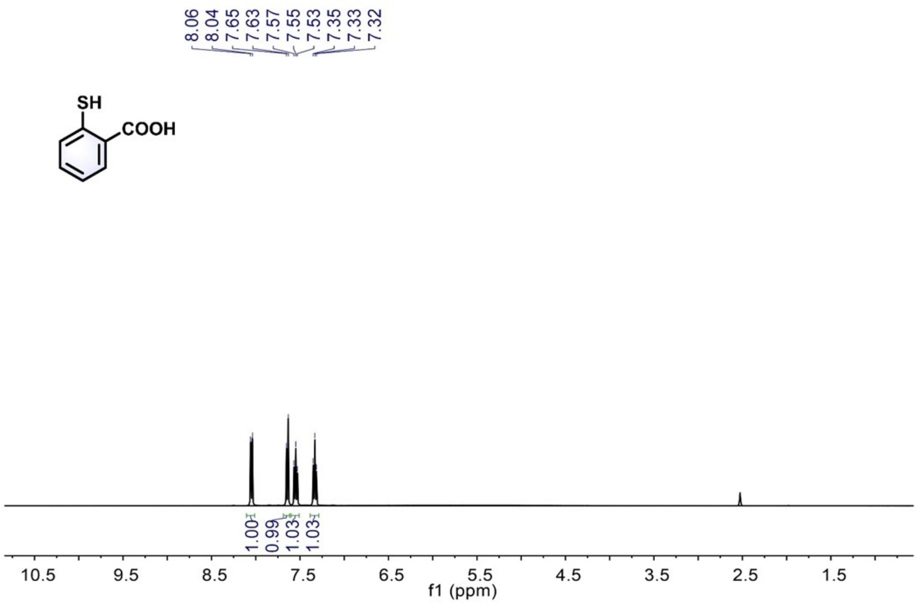

Figure S73. The ^{1}H NMR spectra of ***o*-COOH** in $CDCl_3$. ^{1}H NMR (400 MHz, DMSO) δ 8.05 (d, J = 9.1 Hz, 1H), 7.64 (d, J = 8.9 Hz, 1H), 7.55 (t, J = 7.7 Hz, 1H), 7.33 (t, J = 7.5 Hz, 1H).

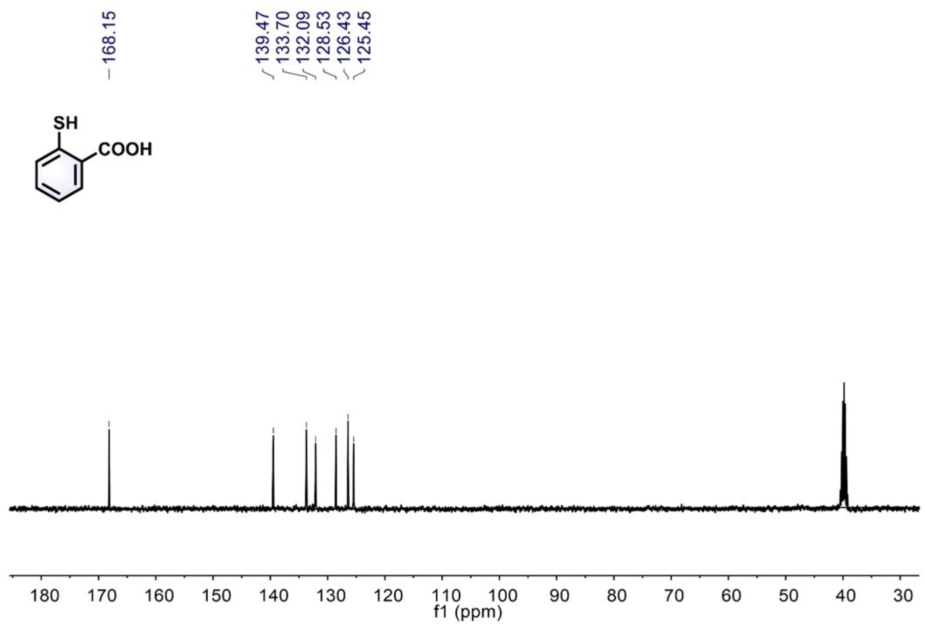

Figure S74. The ^{13}C NMR spectra of ***o*-COOH** in $CDCl_3$. ^{13}C NMR (101 MHz, DMSO) δ 168.15 (s), 139.47 (s), 133.70 (s), 132.09 (s), 128.53 (s), 126.43 (s), 125.45 (s).
